# Diet X Gene Interactions Control Femoral Bone Adaptation to Low Dietary Calcium

**DOI:** 10.1002/jbm4.10668

**Published:** 2022-08-19

**Authors:** Krittikan Chanpaisaeng, Perla C. Reyes‐Fernandez, Brian Dilkes, James C. Fleet

**Affiliations:** ^1^ Functional Ingredients and Food Innovation Research Group, National Center for Genetic Engineering and Biotechnology (BIOTEC), National Science and Technology Development Agency (NSTDA) Pathum Thani Thailand; ^2^ School of Health and Human Sciences, Department of Physical Therapy Indiana University–Purdue University Indianapolis Indianapolis IN USA; ^3^ Center for Plant Biology Purdue University West Lafayette IN USA; ^4^ Department of Biochemistry Purdue University West Lafayette IN USA; ^5^ Department of Nutritional Sciences and the Dell Pediatric Research Institute University of Texas Austin TX USA

**Keywords:** BONE ΜCT, GENETIC ANIMAL MODELS, NUTRITION

## Abstract

Genetics and dietary calcium (Ca) are each critical regulators of peak bone mass but it is unclear how genetics alters the physiologic response of bone to dietary Ca restriction (RCR). Here, we conducted genetic mapping in C57BL/6J × DBA/2J (BXD) recombinant inbred mouse lines to identify environmentally sensitive loci controlling whole‐bone mass (bone mineral density [BMD], bone mineral content [BMC]), distal trabecular bone, and cortical bone midshaft of the femur. Mice were fed adequate (basal) or low Ca diets from 4–12 weeks of age. Femurs were then examined by dual‐energy X‐ray absorptiometry (DXA) and micro‐computed tomography (μCT). Body size–corrected residuals were used for statistical analysis, genetic mapping, and to estimate narrow sense heritability (h^2^). Genetics had a strong impact on femoral traits (eg, bone volume fraction [BV/TV] basal Ca, h^2^ = 0.60) as well as their RCR (eg, BV/TV, h^2^ = 0.32). Quantitative trait locus (QTL) mapping identified up to six loci affecting each bone trait. A subset of loci was detected in both diet groups, providing replication of environmentally robust genetic effects. Several loci control multiple bone phenotypes suggesting the existence of genetic pleiotropy. QTL controlling the bone RCR did not overlap with basal diet QTL, demonstrating genetic independence of those traits. Candidate genes underlying select multi‐trait loci were prioritized by protein coding effects or gene expression differences in bone cells. These include candidate alleles in *Rictor* (chromosome [chr] 15) and *Egfl7* (chr 2) at loci affecting bone in the basal or low Ca groups and in *Msr1* (chr 8), *Apc*, and *Camk4* (chr 18) at loci affecting RCR. By carefully controlling dietary Ca and measuring traits in age‐matched mice we identified novel genetic loci determining bone mass/microarchitecture of the distal femur as well as their physiologic adaptation to inadequate dietary Ca intake. © 2022 The Authors. *JBMR Plus* published by Wiley Periodicals LLC on behalf of American Society for Bone and Mineral Research.

## Graphical abstract

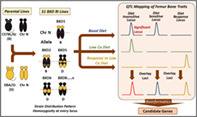

## Introduction

Bone strength is a strong predictor for osteoporotic fractures. In the clinical setting, bone mineral density (BMD) and bone mineral content (BMC) are the gold standards for evaluating the risk of osteoporotic fractures. However, bone strength is also determined by bone material properties, geometry, and microarchitecture.^(^ ^1^ ^)^ Fractures commonly occur at trabecular (Tb) bone‐rich sites such as femur ends and spine^(^ ^2^ ^)^ and reduced Tb volume fraction, number, and connectivity are strongly associated with susceptibility to structural failure or bone breakage.^(^ ^3^, ^4^, ^5^ ^)^ Because Tb has a high surface‐to‐volume ratio it is also very responsive to physiologic needs for maintaining serum calcium (Ca), eg, during lactation^(^ ^6^ ^)^ and in ovariectomized rats.^(^ ^7^ ^)^ Thus, in order to prevent osteoporotic fractures later in life, it is paramount to accumulate high peak bone mass as well as establish a highly connected Tb microarchitecture.

Bone phenotypes are under genetic regulation^(^ ^8^, ^9^ ^)^ and display continuous distributions within the population. Genome‐Wide association studies (GWASs) in humans^(^ ^9^ ^)^ and genetic mapping studies in mouse models^(^ ^8^ ^)^ have reported many chromosomal locations or quantitative trait loci (QTLs) that underlie normal variation of bone features. In addition, studies show that the genetic loci controlling Tb phenotypes are distinct from those that control BMD and BMC.^(^ ^10^, ^11^, ^12^, ^13^ ^)^ Collectively, these studies suggest there are unique genetic regulators for Tb microarchitecture that are hidden in the genetic analysis of BMD or are distinct from the genetic regulators controlling cortical bone phenotypes.

In addition to genetics, environmental factors like dietary Ca play an important role in bone development.^(^ ^14^ ^)^ Adequate dietary Ca improves peak bone mass by supplying Ca for bone mineralization but its effect is modified by genetics. For example, in experiments where subjects were fed diets with varying amounts of Ca (760 to ~2000 mg/d), adolescent African American girls had greater skeletal Ca retention, higher 1,25‐dihydroxyvitamin D_3_ (1,25(OH)_2_D_3_), and higher Ca absorption than Tanner stage–matched white girls.^(^ ^15^, ^16^ ^)^ Similarly, we have shown that both BMD and Tb phenotypes, as well as their response to low dietary Ca intake, are highly variable across 11 genetically diverse inbred mouse lines.^(^ ^17^ ^)^ Because a majority of the people in the United States and worldwide have inadequate Ca intake,^(^ ^18^, ^19^ ^)^ identifying genetic variants that regulate the differential responses of individuals to low Ca intake is an integral step toward developing personalized dietary interventions that promote optimal bone mass. Toward this end, we conducted a genetic mapping in 51 C57BL/6J × DBA/2J (BXD) recombinant inbred (RI) mouse lines and identified candidate genes underlying the genetic response of BMD and BMC to dietary Ca restriction during growth.^(^ ^20^ ^)^ However, no studies have examined how genetics and diet interact to affect the development of bone structure.

We have extended our previous work by investigating the genetic factors that control cortical (Ct) and Tb phenotypes. By using a large diverse population of isogenic, recombinant inbred mouse lines, we were able to control both the genetics and the dietary environment that influences the development of Tb mass and its microarchitecture, as well as cross‐sectional Ct geometry. Thus, this study is the first to systematically test whether gene × diet (G×D) interactions influence these bone phenotypes and to identify loci controlling the physiologic response of bone to inadequate dietary Ca intake.

## Materials and Methods

### Experimental design

We report an extension of a study we conducted to assess the impact of genetics on calcium and vitamin D metabolism.^(^ ^20^, ^21^ ^)^ We used a population of 51 BXD recombinant inbred (RI) mouse lines for this experiment. BXD RI lines were derived by inbreeding F2 mice from a cross of the C57BL/6J (B6) and DBA/2J (DBA) inbred mouse lines until all of the alleles were fixed to homozygosity.^(^ ^22^ ^)^ Like F2 mice, each RI line has a unique recombination pattern of alleles from the parent lines. However, because the alleles are fixed in each BXD line, the genetic features can be replicated and studied in multiple environments. This permits the examination of gene × environment interactions.

Four‐week‐old male mice from the 51 lines, as well as mice from the two parental lines, were obtained from The Jackson Laboratory (Bar Harbor, ME, USA). Upon arrival, 16 mice from each line, and 8 mice from BXD36, were randomly assigned to either a 0.5% (basal) Ca or 0.25% (low) Ca diet (AIN93G base with 200 IU vitamin D_3_/kg diet; Research Diets, New Brunswick, NJ, USA) (*n* = 8–9 per diet group for all lines except BXD36 where *n* = 4). The total of 809 mice were randomized to dietary groups; data for 787 mice were available for analysis (see Table S1). The dietary Ca levels were chosen to meet the National Research Council (NRC) rodent dietary Ca requirement (0.5% Ca) or to model the low level of dietary Ca intake seen in the US population (0.25% Ca).^(^ ^18^ ^)^ Mice were group‐housed (2–4 mice/cage) at the Purdue University animal facilities in conventional shoebox cages, maintained in rooms with ultraviolet (UV)‐blocking filters over lights and a 12‐hour light/dark cycle, and provided food and distilled water *ad libitum*. At 12 weeks of age, at a point where others have shown that mice reach peak trabecular bone mass,^(^ ^23^ ^)^ the mice were fasted overnight, after which the right femurs were harvested and prepared for analysis as described.^(^ ^20^ ^)^ Investigators were blinded to genotype and dietary treatment, animal handling, bone sample collection, and endpoint measurements. All animal experiments complied with the Animals in Research: Reporting In Vivo Experiments (ARRIVE) guidelines and the Purdue Animal Care and Use Committee approved the experimental protocol.

### Dual X‐ray absorptiometry scan

After removal of muscle from the fixed femurs, femur length was recorded using a digital caliper (Mitutoyo America Corporation, Aurora, IL, USA). BMC (g) and BMD (g/cm^2^) were determined using a PIXImus II densitometer (Lunar, GE Healthcare, Madison, WI, USA). Scans were conducted in air on 10 bones at a time placed on the company‐supplied plastic imaging plate (the isotropic pixel dimension is 0.18 mm, alternating dual energy levels = 80 and 40 kV, current = 400 mA, fixed threshold of 1320). Samples from each genetic line and each diet group were randomized to the scans and to one of 10 uniformly‐spaced positions in the center 70% of the scan area to minimize potential scan position effects. Regions of interest (ROIs) were manually set for each bone and analysis was performed by one investigator. The coefficient of variation for measurements of femurs is 3% to 5%.^(^ ^24^ ^)^

### Micro‐computed tomography evaluation

Femurs were analyzed using micro‐computed tomography (μCT) (μCT 40; Scanco Medical AG, Bassersdorf, Switzerland) with scanning parameter settings reported elsewhere.^(^ ^17^ ^)^ Briefly, femurs were scanned at the isotropic voxel size of 16 μm using an energy level of 55 kVp, an integration time of 300 ms, and an X‐ray tube current of 145 μA.

The trabecular bone scan captured 1.664 mm (104 slices) proximal to the apex of distal condyle. The ROI for quantification was 0.896 mm (56 slices) proximal to the first slice containing no evidence of distal growth plate. The ROI for Ct bone was defined as 0.48 mm (30 slices) at the mid‐section of diaphysis to ensure that the third trochanter was not included (see Fig. S1 for images showing the quantification area).

Using Scanco evaluation software (version 6), each femur was assessed for both trabecular and cortical bone parameters. For trabecular bone, we measured bone volume fraction (BV/TV), trabecular number (Tb.N, mm^−1^), trabecular thickness (Tb.Th, mm), trabecular separation (Tb.Sp, mm), connectivity density (Conn.D, 1/mm^3^), structure model index (SMI), and trabecular bone tissue mineral density (Tb.TMD) as recommended.^(^ ^25^ ^)^ We manually contoured trabecular bone every 10 slices with the outline two to three pixels away from the cortical bone, and the intermediate slices were interpolated with the contouring algorithm in the software to create a volume of interest. Morphometric parameters were evaluated using a Gaussian filter = 0.8 and a threshold of 220 in the 1/1000 unit (474.3 mg hydroxyapatite [HA]/cm^3^). For cortical bone we measured the cross‐sectional parameters of Ct bone geometry including cortical thickness (Ct.Th, mm^2^), cortical area (Ct.Ar, mm^2^), and total area (Tt.Ar, mm^2^), defined the cortical area fraction (Ct.Ar/Tt.Ar, %) and also the polar moment of inertia (*J*, mm^4^), moment of inertia around the shorter axis divided by maximum distance from the centroid perpendicular to the Imax direction (Imax/Cmax, mm^3^), and moment of inertia around the longer axis divided by maximum distance from the centroid perpendicular to the Imin direction (Imin/Cmin, mm^3^). After contouring, the Ct parameters were evaluated using a Gaussian filter = 0.8 and a threshold of 310 in the 1/1000 unit (505.7 mg HA/cm^3^).

### Statistical analysis

Statistical analysis was conducted using SAS Enterprise Guide 6.1 (SAS Institute Inc., Cary, NC, USA). Data points with a *Z*‐score in the extreme 2.5% of either end of a line/diet group distribution were removed as outliers. In addition, if a mouse died prior to the end of the experiment or if the bone was damaged during sample preparation, the femur was not analyzed. Table S1 reports the number of mice in each genotype and dietary treatment group for each μCT parameter after outlier removal. In addition to the data obtained from each mouse on each diet, a parameter reflecting the response to dietary Ca restriction (RCR) was calculated as the percent difference between the phenotypic value for an individual (i) fed the low Ca diet (x) and the line (j) mean for the phenotype value from the basal Ca diet (y), standardized to the line mean for the phenotypic value from the basal Ca diet and multiplied by 100, ie, $\left[\left(x_{\mathrm{ij}}-{\bar{y}}_{j}\right)∕{\bar{y}}_{j}\right]*100$.^(^ ^17^ ^)^ All raw line mean data are provided in Table S2. For each phenotype, the covariate effect of body weight (BW) and/or femur length (FL) was determined by Pearson's correlation and, when significant, removed by linear regression.^(^ ^26^ ^)^ When the body size (BS)‐corrected residuals were not normally distributed, square root, natural log, or cube root transformations were applied as needed (Table S3). RCR traits included negative values. As a result, prior to transformation a constant value was added to each trait value so that no negative data points existed (Table S3).

Line means of BS‐corrected residuals for the 51 BXD RI lines under each dietary condition (basal, low Ca and RCR) were used for genetic mapping (Table S4). To reflect the variation among lines, *Z*‐scores were calculated from the line means of BS‐corrected residuals of the 51 BXD RI lines and their parental lines. We assessed the narrow‐sense heritability (h^2^) of each phenotype using the *r* ^2^ from a one‐way analysis of variance (ANOVA) (main effect = genotype); this was conducted separately for each diet group as well as for their RCR. Two‐way ANOVA of BS‐corrected residuals was used to test the main effects and interaction effects (ie, genotype‐by‐diet, G×D) on each phenotype. G×D interactions were also determined by conducting one‐way ANOVA to assess the impact of genetics on the RCR. Using 51 BXD RI lines allowed us to detect QTL accounting for 15% of the variance observed in the population with the power ~0.80 and α = 0.05.^(^ ^27^ ^)^

### QTL mapping

BXD genetic markers were downloaded from the GeneNetwork (http://gn1.genenetwork.org/webqtl/main.py?FormID=sharinginfo&GN_AccessionID=600&InfoPageName=BXDGeno). For the 198 BXD strains, the file contains 7321 markers and provides approximate locations for 10,300 recombinations (average of 52 per strain) (http://www.genenetwork.org/webqtl/main.py?FormID=sharinginfo&GN_AccessionId=600). Genetic marker locations are reported in base pairs (bp) based on the mouse genome build GRCm38/mm10. The Mouse Map Converter (MMC) tool on the Jackson Lab Center for Genome Dynamics (http://churchill-lab.jax.org/mousemapconverter/) was used to convert mm10 coordinates to sex‐average centiMorgan (cM) values that were then used for QTL mapping analysis. Many of the original markers were not informative because we used only 51 of the 198 BXD lines. After excluding genotype data of lines outside the 51 lines, we used the findDupMarkers and drop.marker functions available in the R/qtl package (https://cran.r-project.org/) to remove markers with duplicate genetic locations or perfectly correlated genotypes. The final genetic map for the 51 lines contained 2244 markers (available on request).

Composite interval mapping (CIM) was conducted on BS‐corrected line means (*n* = 51) using Windows QTL Cartographer v2.5_011 (https://brcwebportal.cos.ncsu.edu/qtlcart/WQTLCart.htm). Forward selection identified 5 significant background markers. CIM was carried out using a Haldane map function, 2 cM walking speed, and a 10 cM window. Phenotypes for each diet (0.5% or 0.25% Ca) group and the RCR were mapped separately. For each analysis, permutations (*n* = 1000) were used to determine significance threshold in a logarithm (base 10) of odds (LOD) unit. A peak with LOD ≥ the computed permutation threshold was considered significant. Because of our interest in pleiotropy, we also tracked peaks with LOD ≥2, a level that many reports use was to identify putative QTL.^(^ ^28^ ^)^

For each significant or putative QTL, the 1.5‐LOD support region was used for candidate gene identification.^(^ ^29^ ^)^ This region is considered as an equivalent to 95% confidence interval (CI).^(^ ^30^, ^31^ ^)^ The downstream and upstream boundaries of this region in cM were converted to mm10 base pair positions (GRCm38) using the Mouse Map Converter tool.^(^ ^32^ ^)^

### Prioritization of QTL candidate regions

We identified many loci where multiple phenotype QTL co‐localized. We grouped these co‐localized QTL into a single locus and assigned an identification (loci ID). A comprehensive list of these loci with the assigned loci ID is shown in Table S5.

Using this list of loci, we then selected several loci for further investigation. We prioritized loci based on the following criteria: (i) A significant QTL for a single trait (high Ca, low Ca) with an LOD >5; (ii) A significant QTL observed in an RCR phenotype with and LOD >5; (iii) significant (or one significant with one putative) QTL observed for the same phenotype in both the basal and low Ca groups and with a matched parental influence; (iv) multiple QTL observed within the same condition (≥3 basal, low Ca or ≥2 RCR) or within the low Ca group and RCR with a matched parental influence; and (v) a locus with five or more significant or putative QTL. We gave a score of 1 for each criterion that was met. These criteria are not mutually exclusive, eg, a single locus may earn a score for multiple of the criteria. The loci with a score of 3 or greater were considered as *high‐priority* loci that were then considered for in‐depth bioinformatics analysis.

### Bioinformatic characterization of loci

**Fig. 1:**
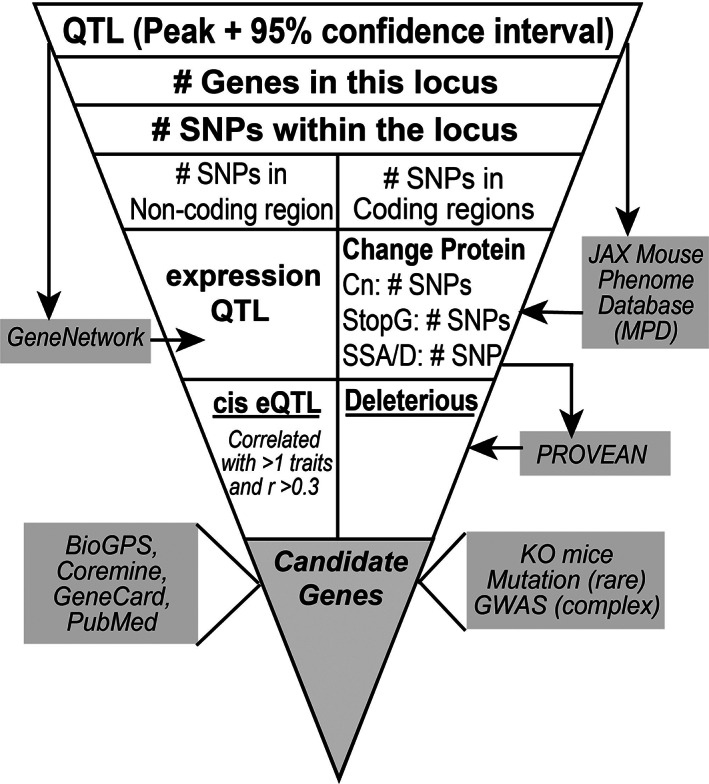
The bioinformatic analysis pipeline used to characterize high priority loci and to identify candidate genes. Abbreviations: Cn = nonsynonymous amino acid substitutions; *cis* eQTL = expression QTL acting in *cis*; SSA/D = splice site addition/deletion; StopL/G = loss or gain of a stop codon.

The process we used to evaluate high‐priority loci is summarized in Fig. 1. For each locus, single nucleotide polymorphisms (SNPs) between B6 and DBA mice were obtained using the Mouse Phenome Database (MPD, https://phenome.jax.org/snp/retrievals)^(^ ^33^ ^)^ with the Sanger data set that contains data on >89 million SNP and indels from 37 inbred strains of mice.^(^ ^34^ ^)^ MPD annotations (NCBI dbSNP 138) were used to categorize polymorphisms by gene attribute; ie, intronic and noncoding, insertions/deletions, messenger RNA (mRNA) untranslated region (5′ and 3′ UTR), and exon‐associated (ie, synonymous and nonsynonymous codons, stop codons, splice sites, or frameshift mutations).

Genes with SNPs that lead to gain or loss of a stop codon, cause a frameshift, and/or alter a splice site were automatically considered candidate genes. Effects of nonsynonymous amino acid changes were examined for potential functional effects using PROVEAN v1.1 (score < −2.5; https://www.jcvi.org/research/provean)^(^ ^35^ ^)^ and SIFT v4.0.3 (score <0.05; https://sift.bii.a-star.edu.sg/).^(^ ^36^ ^)^ Genes with deleterious/damaging SNPs identified by at least one tool were considered as candidate genes.

Local (*cis*) expression QTL (eQTL) analysis was conducted in silico using publicly available microarray data from the femur mRNA of the BXD panel (GN accession: GN414) that is available at the GeneNetwork (http://gn1.genenetwork.org/webqtl/main.py). Searches were conducted on the 1.5‐LOD support interval of each locus. We defined the LRS threshold as >13.6 (LOD = ~3) to capture both significant and putative eQTL. Of these eQTL, we examined the correlation between the abundance of a gene transcript within the *cis* eQTL and BS‐corrected line means of each phenotype mapped to the locus. Genes with significant eQTL whose SNP is within the transcribed region of the regulated gene or within 1 Mb of the transcribed region (ie, cis eQTL), and whose expression level correlated with our phenotypes (Pearson's correlations, *p* < 0.05) were considered as candidate genes.

We assessed whether candidate genes are likely to have function‐altering polymorphisms influencing bone microarchitecture by searching the literature and public databases for information that links the candidate genes to bone biology. The list of criteria included: (i) a significant association with bone phenotypes in GWAS; (ii) a functional role in bone from gene knockout mice (from the International Mouse Phenotyping Consortium [IMPC] and the International Mouse Strain Resource [IMSR] databases); (iii) evidence from animal or cell studies identifying a mechanistic role for the candidate gene in bone cell biology or other pathways known to influence bone phenotypes (from Coremine; https://www.coremine.com/medical/); and (iv) links to gene ontology (GO) terms related to bone (from the Mouse Genome Informatics database [MGI]; http://www.informatics.jax.org/); and (v) high expression in bone cells (BioGPS; http://biogps.org/).

## Results

### The effect of genetics (G), dietary Ca intake (D), and GXD interactions on Tb phenotypes and their heritability

All mice were healthy throughout the study. Body weight (BW) and femur length (FL) were significantly different among the lines (*p* < 0.0001), but they were not affected by dietary Ca intake (*p* = 0.36 and 0.18, respectively). Because there is variation in BW and FL in the BXD population, all phenotypic data were examined for the effect of body size and covariate adjustments were made when necessary (see Materials and Methods, Table S2). Body size (BS)‐corrected line means for all phenotypes on each diet and RCR are provided in Table S4. Within and across each group (basal Ca, low Ca and RCR), many traits were highly correlated to one another (*p* < 0.0001, Table S6).

**Fig. 2:**
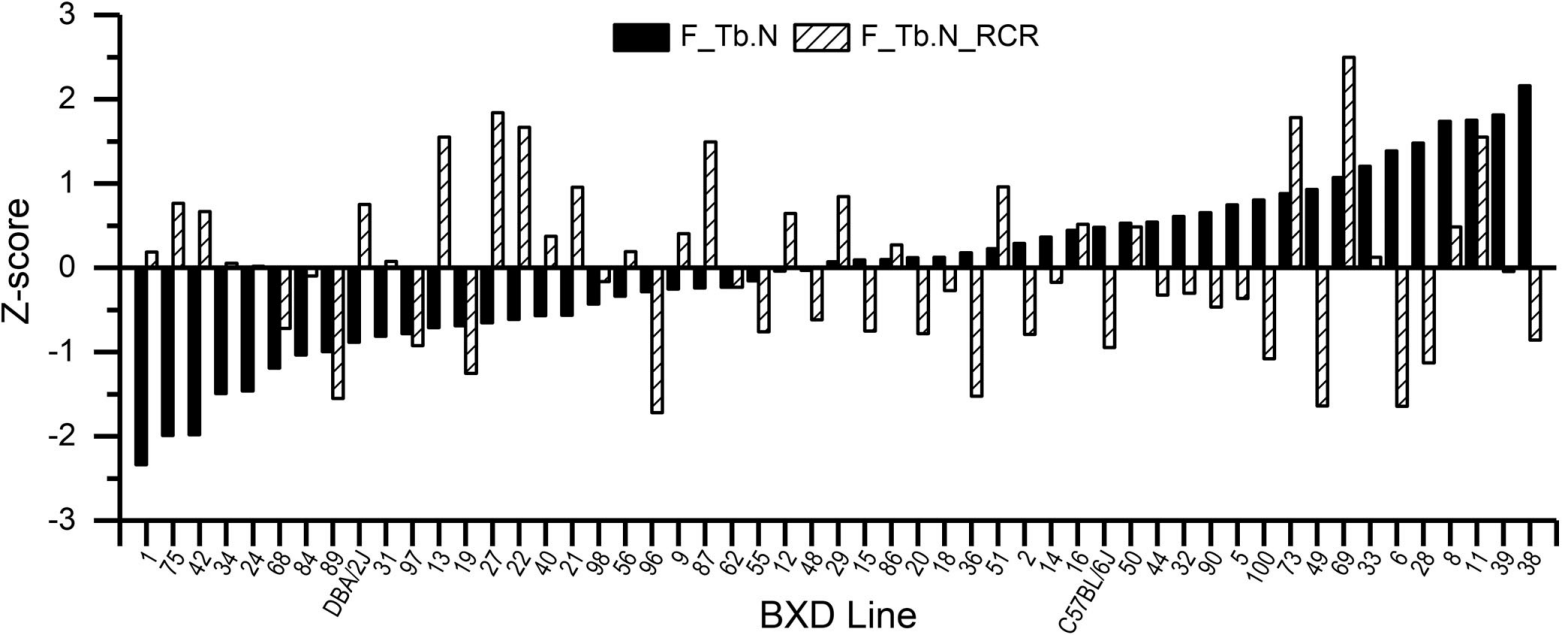
Distinct variation affects femur trabecular bone number (F_Tb.N) on the basal diet and the response of F_Tb.N to dietary Ca restriction (RCR). Data were plotted for 51 BXD RI lines and the two parent mouse lines (C57BL/6J, DBA/2J). *Z*‐scores of body size corrected Tb.N from the basal Ca diet (filled bars) and Tb.N RCR values (hatched bars) are presented. Lines are ordered from the smallest to largest *Z*‐score of the basal Tb.N group. RCR = response of bone to dietary Ca restriction.

Dietary Ca intake had a significant impact on BMD, BMC, Ct.Ar, Ct.Ar/Tt.Ar, Ct.Th, Imax/Cmax, and all Tb phenotypes (*p* < 0.05) except Tb.TMD (*p* = 0.35). There was significant variation across the lines for all of the Tb traits in the basal diet group and for their corresponding RCR (Fig. 2, Table S4). Genetics had a strong effect (*p* < 0.0001) on all femoral phenotypes with heritability estimates (h^2^) ranging from 0.40–0.80 in the basal diet group (with the highest and lowest values observed in Ct.Ar/Tt.Ar and BMC, respectively), and from 0.46–0.83 in the low Ca diet group (highest in Ct.Ar/Tt.Ar and lowest in BMC, SMI, and Tb.TMD) (Table S7). The variability of the basal phenotypes was different from the variability of the RCR phenotypes (Fig. 2). ANOVA identified a significant G×D interaction effect (*p* < 0.05) for BV/TV, Tb.Th, and Tb.TMD. The RCR parameters of all phenotypes were also affected by genetics (*p* < 0.001), and their heritability estimates ranged from 0.23 to 0.41 (Table S7).

### Genetic mapping

**Fig. 3:**
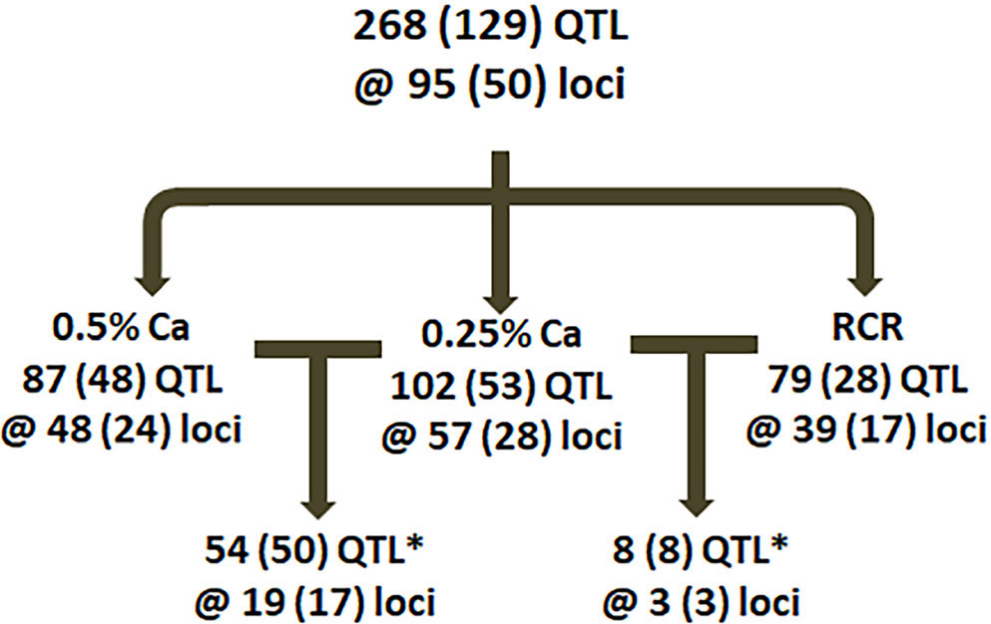
A summary of total and significant QTL affecting femur μCT parameters. Loci are provided for each dietary calcium (Ca) group and for the RCR parameter. Values are presented at total QTL and significant loci (ie, those that met the significance threshold). *Overlap between loci in groups. Note: Only QTL where the same trait was seen at the same loci under both conditions are reported. The significant overlap count requires that at least one of the two QTL for a phenotype be significant. Divide the overlap number by 2 to calculate the number of phenotype QTL that are seen in a comparison (eg, 54 high/low diet Ca QTL represent 27 phenotypes at 19 loci).

**Fig. 4:**
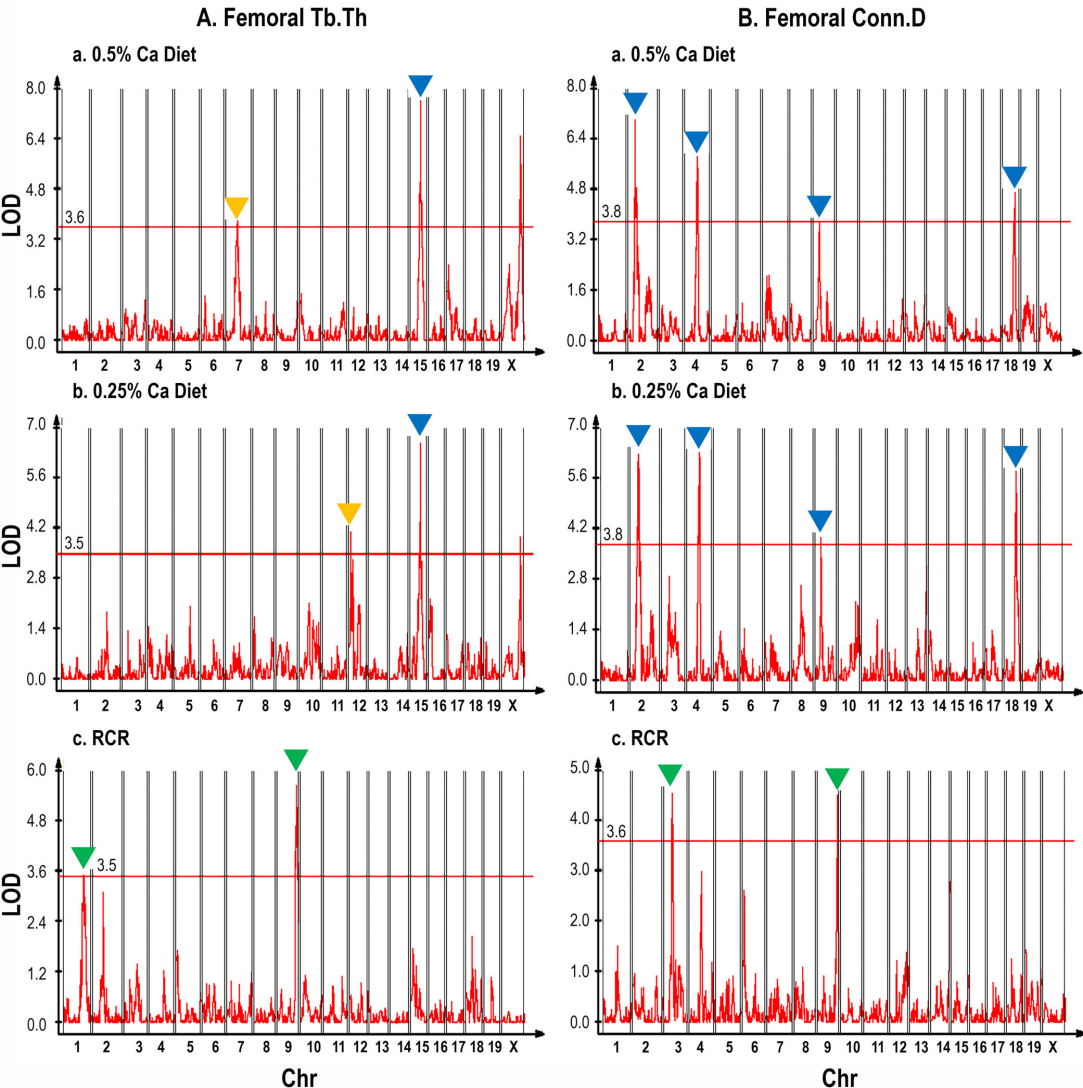
Composite interval maps for femoral (*A*) Tb.Th, and (*B*) Conn.D. For each phenotype, the genetic maps of basal, low Ca, and RCR phenotypes are presented in a, b, and c, respectively. Blue arrowheads point to loci that control the phenotype in both basal and low Ca environments. Yellow arrowheads point to loci that are present in one dietary group but not the other. Green arrowheads point to loci controlling the RCR phenotype. Conn.D = connectivity density; Tb.Th = trabecular thickness.

**Fig. 5:**
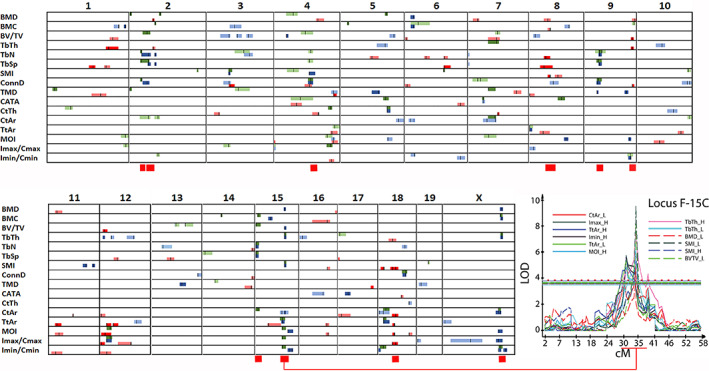
A summary of QTL identified for all 16 femoral bone traits in the 0.5% Ca or 0.2% Ca diet groups or in RCR. The confidence interval for each QTL is represented as a colored bar (green = 0.5% Ca, blue = 0.2% Ca, and red = RCR) and the peak location of each QTL is shown as a vertical black line within the bar. Putative QTL are shown as a lighter shade of each group color. The 10 high priority multi‐trait loci chosen for bioinformatic analysis are identified by red bars at the bottom of a chromosome. The inset shows the line plot for the individual phenotype QTLs in the F‐15C locus. RCR = response of bone to dietary Ca restriction.

Across 16 traits and three conditions we measured, our QTL mapping analysis identified a total of 95 loci representing 268 significant and putative QTL (Figs. 3 and S2, Table S5). This includes 48 significant and 39 putative QTL in the basal diet group phenotypes, 53 significant and 49 putative QTL in the low Ca diet group phenotypes, and 28 significant and 51 putative QTL for the RCR phenotypes (Fig. 3). At 19 loci, we found significant or putative QTL in both the low (L) and basal (H) diet groups for a phenotype (27 total H/L QTL; 18 H/L loci where both QTL were significant; 7 H/L QTL where one was significant and the other putative; 2 H/L QTL where both were putative) (Fig. 3). These 27 joint H/L QTL reflect independent validation of loci that are insensitive to the dietary intervention. It was less common to find a phenotype QTL for RCR and low Ca diet groups at the same loci (8 QTL at 4 loci). This shows that the loci controlling phenotypes on the basal or low Ca diets are genetically distinct from those controlling the bone response to low Ca intake. For example, in the mapping results for Tb.Th (Fig. 4A.a. and 4A.b.) and Conn.D (Fig. 4B.a. and 4B.b.) the QTL from the basal and low Ca groups overlap, whereas the QTL for RCR (Fig. 4A.c., 4B.c.) and the individual diet groups do not. The location for all of the putative and significant QTL we identified are summarized in Fig. 5. This reveals that there are several loci where multiple QTL converge, most notably for the basal diet phenotype QTL on Chr 15 and X and for RCR QTL on Chr 8 and 18.

### Prioritization of mapped loci

We prioritized loci for bioinformatic follow‐up analysis and narrowed our interest to 10 multi‐trait loci (Table 1 and Fig. S3 for combined multi‐trait maps of these 10 loci). These 10 loci included mostly significant QTL (64/75 QTL, all >50% significant, none less than 3 significant QTL) with high LOD (38 > 5). Seven of the 10 loci contained multiple phenotypes that had QTL in both the basal and low Ca diet groups: F‐2B, F‐2C, F‐4E, F‐9A, F‐15A, F‐15C, and F‐20C (F = femur, # = Chromosome, Letter = ID when multiple loci where on a chromosome). The F‐15C locus had the highest number of overlapping QTL (*n* = 13 total and 10 significant) and included three traits with QTL from both diet groups (ie, Tt.Ar, Tb.Th, and SMI, all driven by the B6 allele) (Fig. 5). The three other high‐priority loci (F‐8C, F‐9D, and F‐18C) contained QTL that control ≥3 RCR phenotypes; eg, F‐18C includes RCR QTL for Imax/Cmax, Tb.Th, Tt.Ar, *J*, CtAr, and SMI all driven by the DBA allele.

**Table 1:** Selected Candidate Genes in High Priority Loci

Loci ID	Chr	1.5‐LOD CI^a^ (Mb)	QTL^e^ information	DXA parameters		Trabecular parameters							Cortical parameters							Candidate genes^d^ (type)
				BMD	BMC	BV/TV	Tb.Th	Tb.N	Tb.Sp	Conn.D	SMI	Tb.TMD	Ct.Ar	Tt.Ar	Ct.Ar/Tt.Ar	Ct.Th	*J*	*Imax/Cmax*	*Imin/Cmin*	
F‐2B	2	26.56–31.01	Trait condition					Basal/Low	Basal	Basal										Egfl7 (Cn)
			Allelic effect^b^					B6	DBA	B6										
			LOD^c^					4.49, 4.52	5.3	7.01										
F‐2C	2	33.04–49.66	Trait condition			Basal		Low	Basal, Low	Low			Basal							Traf1 (eQTL)
			Allelic effect			B6		B6	DBA	B6			B6							
			LOD			4.22		5.06	5.86, 5.90	6.29			2.31							
F‐4E	4	81.54–92.00	Trait condition			Basal		Basal	Basal	Basal, Low	Low									Cdkn2b (eQTL)
			Allelic effect			B6		B6	DBA	B6	DBA									
			LOD			3.07		2.83	2.86	6.33, 5.85	4.95									
F‐8C	8	26.98–49.66	Trait condition	RCR	RCR			RCR	RCR		RCR			RCR						Msr1 (Cn)
			Allelic effect	DBA	DBA			DBA	B6		B6			DBA						
			LOD	2.26	3.2			5.12	4.65		3.97			2.34						
F‐9A	9	29.82–40.50	Trait condition					Basal, Low	Basal, Low	Basal, Low		Low								Ets1 (eQTL)
			Allelic effect					DBA	B6	DBA		B6								
			LOD					4.06, 4.79	4.61, 5.47	3.81, 3.95		9.43								
F‐9D	9	112.62–116.61	Trait condition	RCR		RCR	RCR			RCR										Ppp2r3a (eQTL)
			Allelic effect	DBA		DBA	DBA			DBA										
			LOD	2.25		4.2	5.64			4.51										
F‐15A	15	0.53–12.11	Trait condition		Basal	Basal		Basal/ Low	Basal/ Low				Basal							Rictor (eQTL)
			Allelic effect		B6	B6		B6	DBA				B6							
			LOD		5.43	3.98		4.70, 5.55	4.23, 6.45				4.93							
F‐15C	15	59.80–72.53	Trait condition	Low		Low	Basal/ Low				Basal/Low		Low	Basal/Low			Basal	Basal	Basal	Fam135b (Cn)
			Allelic effect	B6		B6	B6				DBA		B6	B6			B6	B6	B6	
			LOD	3.93		8.17	7.61, 6.58				6.06, 9.56		5.08	5.74, 5.48			4.95	5.81	5.66	
F‐18C	18	29.98–47.08	Trait condition				RCR				RCR		RCR	RCR			RCR	RCR		Apc (Cn)
			Allelic effect				B6				DBA		B6	B6			B6	B6		Camk4 (eQTL)
			LOD				2.05				4.83		5.13	5.37			4.85	5.91		
S‐20C	X	51.90–69.44	Trait condition	Low	Basal, Low		Basal, Low						Basal, Low				Low	Low	Basal, Low	Vgll1 (Cn)
			Allelic effect	B6	B6		B6						B6				B6	B6	B6	
			LOD	4.5	9.74, 5.39		6.51, 3.96						8.26, 7.04				6.16	3.62	8.05, 9.8	

^a^ 1.5‐LOD confidence intervals in megabase location (Build GRCm38/mm10).

^b^ Parental influence: B6 = C57BL/6J line, DBA = DBA/2J line.

^c^ Phenotype‐specific LOD score for each locus.

^d^ This list does not include predicted genes.

^e^ Basal = values from the adequate calcium group; BV/TV = bone volume fraction; Cf = a mutation involving the deletion or insertion of one or more bases; Chr = chromosome; Cn = genes with polymorphisms scored as potentially deleterious nonsynonymous amino acid substitutions; Conn.D = connectivity density (1/mm3); Ct.Ar = cortical area (mm^2^); Ct.Ar/Tt.Ar = cortical area fraction; Imin/Cmin = moment of inertia around the longer axis divided by maximum distance perpendicular to the Imin direction (mm^3^); Imax/Cmax = moment of inertia around the shorter axis divided by maximum distance perpendicular to the Imax direction (mm3); *J* = cortical polar moment of inertia (mm^4^); Low = values from low dietary calcium group; Mb = megabase; RCR = the response to dietary calcium restriction; SMI = structure model index; SSA = splice‐acceptor‐variant; SSD = splice‐donor‐variant; StopG = genes with polymorphisms that cause a premature stop codon; Tb.N = trabecular number (mm‐1); Tb.Sp = trabecular separation (mm); Tb.Th = trabecular thickness (mm); Tb.TMD = trabecular tissue mineral density (mg of hydroxyapatite/cm^3^); Tt.Ar = total area (mm^2^).

### Bioinformatic analysis of high‐priority loci

**Fig. 6:**
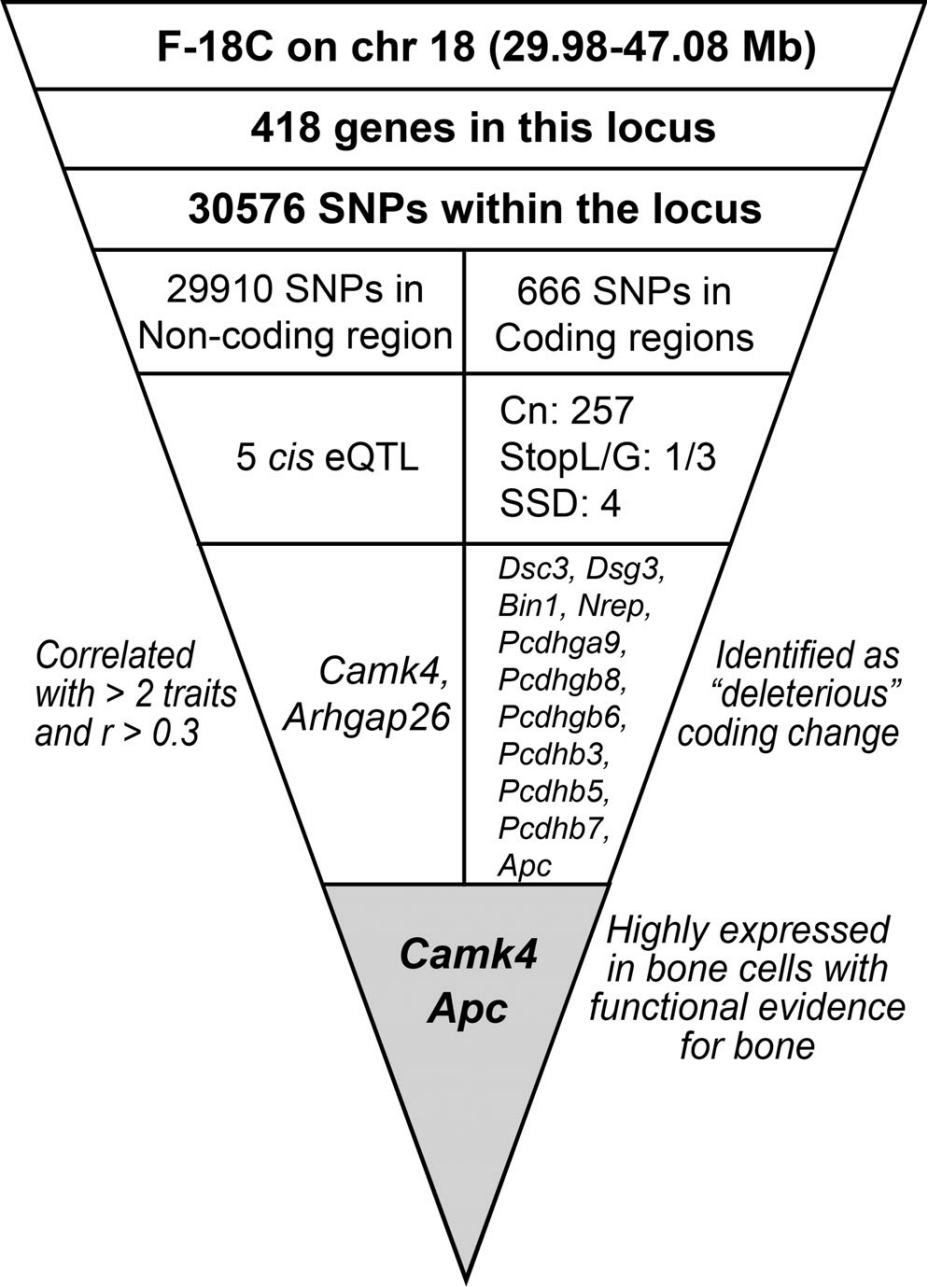
Identification of candidate genes in the high‐priority locus F‐18C. This locus controls six RCR phenotypes including Tt.Ar, Ct.Ar, J, Imax/Cmax, SMI, and Tb.Th. *cis* eQTL = expression QTL acting in *cis*Cn = nonsynonymous amino acid substitutions; SNPs = single nucleotide polymorphisms; SSD = splice site deletion; StopL/G = loss or gain of a stop codon.

The 10 high‐priority loci were subjected to in‐depth, bioinformatics analysis. The number of genome features, genes, functional polymorphisms, and eQTL at each locus is summarized for each of these loci in Table S8. We systematically identified candidate genes in each locus, and we will present F‐18C as an example of this process (Fig. 6). This locus includes QTL for six different RCR phenotypes. The 1.5‐LOD confidence interval for this locus (chr 18, 29.98–47.08 Mb) encompasses 422 genes and 30,571 polymorphisms. In the coding sequence, there are 666 SNPs: 1 SNP causes the loss of a stop codon, 3 SNPs lead to truncated proteins due to the gain of a stop codon, 4 SNPs disrupt splice sites, and 257 SNPs cause nonsynonymous amino acid changes. Nonsynonymous SNPs in 11 genes were predicted to have amino acid changes that are deleterious to protein function (Table 1 and Fig. 6). A candidate gene with a nonsynonymous SNP that we identified from this bioinformatic pipeline is *Apc* (adenomatosis polyposis coli), a negative regulator of Wnt signaling. The bulk of polymorphisms in the F‐18C region affect noncoding sequences. Using published bone gene expression data from The GeneNetwork, we identified eQTL affecting 10 genes at the F‐18C locus. Only one of these, *Camk4*, was a possible *cis* eQTL (LOD = 4.04, distance from gene = 1.05 Mb, mRNA level correlated with the RCR phenotypes that mapped to the locus). By data‐mining the literature, *Apc* and *Camk4* had biological links to bone and were considered as candidate genes for the F‐18C locus.

Following a similar systematic workflow, we identified candidate genes for the other high‐priority loci as summarized in Table 1 and Tables S9 and S10. The supporting evidence that link these candidate genes to bone biology are given in Table S11.

## Discussion

Many studies have used forward genetics approaches to identify loci controlling bone phenotypes^(^ ^8^, ^37^, ^38^ ^)^ including a number of studies in the BXD RI lines.^(^ ^11^, ^13^, ^39^, ^40^, ^41^, ^42^, ^43^ ^)^ However, only our research has examined how genetics interacts with dietary Ca intake to influence Ca and bone metabolism.^(^ ^17^, ^20^, ^44^ ^)^ As such, there are several novel aspects of our work. First, we clearly demonstrate that the penetrance of loci controlling bone mass and μCT phenotypes can be sensitive to the dietary Ca environment. Of the 48 significant QTL we identified in mice fed the basal Ca diet, 23 of these were not observed in mice fed the low Ca diet. The second novel aspect of our research is that we mapped the physiologic response of bone mass and μCT phenotypes to low Ca intake (ie, RCR phenotypes). Of the 28 significant RCR QTL we identified, 24 were not found in either the low dietary Ca group or the basal dietary Ca group. Thus, the genetics controlling the response to dietary restriction is distinct from the genetics controlling the basal bone phenotypes. Our observations of G×D interactions on bone have significant implications for human GWASs where dietary intake is not considered in the search for loci controlling bone biology.

An additional important aspect of our results is that we identified QTL for multiple traits that co‐localized to the same loci (Table S5). A recent study by Watanabe and colleagues^(^ ^45^ ^)^ found that 90% of GWAS loci control multiple traits. Such pleiotropy is more common for traits that are highly correlated, like bone phenotypes (see Table S6).^(^ ^46^ ^)^ The 268 QTL we identified across the 16 traits and three conditions we examined were localized to just 95 loci; 42 of these loci contained three or more different QTL and 17 loci contained five or more QTL. We believe that co‐localization of multiple QTL indicates that the QTL within the loci are less likely to be false positives, even those loci containing QTL with putative LOD scores. Because of this, we selected multi‐trait loci for additional bioinformatic analysis. In the following paragraphs, we discuss strong candidate genes from several of these loci.

The first set of loci we considered were those that included multiple QTL for traits from mice fed the basal diet and included some traits with QTL for both diets. The F‐15A locus included seven significant QTL (Tb.N and Tb.Sp on both diets, and BV/TV, BMC, and Ct.Ar on the adequate Ca diet, Table 1) where higher levels of Tb.N, BV/TV, BMC, and Ct.Ar were driven by the B6 allele. At this locus, an eQTL affecting the *Rictor* mRNA fit the criterion for a *cis* eQTL (max LOD 0.037 Mb from gene). Rictor is a core subunit of the mammalian/mechanistic target of rapamycin complex 2 (mTORC2). Higher *Rictor* mRNA levels were driven by the B6 allele and *Rictor* mRNA levels were correlated with the F‐15A locus bone phenotypes (positive for all but Tb.Sp), suggesting that Rictor promotes accrual of bone mass. Consistent with this, several lines of evidence show that Rictor can promote osteoblast differentiation and optimal bone accrual in vitro^(^ ^47^, ^48^, ^49^ ^)^ and in vivo.^(^ ^49^ ^)^ In addition, mice with osteoclast‐specific *Rictor* ablation have reduced osteoclast formation and increased bone mass, indicating that Rictor is also a negative regulator of osteoclast biology.^(^ ^50^ ^)^ Rictor may also have indirect effects on bone. Adipose‐specific *Rictor* knockout increased body size, including higher bone size and BMC, likely in part due to elevated insulin‐like growth factor 1 (IGF‐1) and insulin‐like growth factor binding protein 3 (IGFBP3) levels.^(^ ^51^ ^)^ We speculated that there might be a counteractive effect of the expression of *Rictor* in adipocytes (high expression suppresses osteoblast activity) and osteoblasts; thus, when there is no *Rictor* expression in adipocytes, Rictor expression in osteoblasts leads to a greater positive effect in bone. We hypothesize that in mice fed high calcium diets the B6 allele in the *Rictor* gene increases its expression in osteoblasts, which promotes bone growth by activating osteoblast commitment and differentiation.

Another locus with co‐localized QTL controlling multiple basal traits is F‐2B (Tb.N [basal and low Ca], Tb.Sp [basal], and Conn.D [basal]; Table 1). Within its 1.5‐LOD CI there is a SNP causing a nonsynonymous amino acid substitution predicted to have a deleterious impact on epidermal growth factor‐like domain‐containing protein 7 (EGFL7), a potent angiogenic factor.^(^ ^52^, ^53^ ^)^ Angiogenesis and endothelial cell function have an important role in promoting osteoblast activity and bone formation.^(^ ^54^ ^)^ In an ex vivo angiogenesis assay recombinant EGFL7 increased blood vessel growth from metatarsal explants by promoting endothelial activity through ERK, STAT3, and integrin signaling.^(^ ^53^ ^)^ We hypothesize that the B6 allele in the *Egfl7* gene encodes a more functional Egfl7 protein as compared to the DBA allele, thus promoting bone angiogenesis and bone growth.

The locus F‐9A also included significant QTL for both basal and low Ca groups of Tb.N, Tb.Sp, and Conn.D that suggest the B6 allele at this locus negatively regulates biology controlling high trabecular bone mass. Co‐localizing with F‐9A is a *cis* eQTL controlling the mRNA level for *Ets1*, a transcription factor that has two bone‐relevant functions. First, it is a co‐regulator of the gene for *Cyp24a1*, an enzyme that degrades 1,25(OH)_2_D.^(^ ^55^ ^)^ We previously mapped a strong, dietary Ca‐insensitive loci for serum 1,25(OH)_2_D to this locus.^(^ ^21^ ^)^ Higher serum 1,25(OH)_2_D levels were driven by the B6 allele and serum 1,25(OH)_2_D levels were negatively correlated with renal *Ets1* mRNA levels. This suggests the B6 allele reduces *Ets1* expression in kidney to reduce 1,25(OH)_2_D degradation and increase serum 1,25(OH)_2_D. *RankL* gene expression is upregulated by 1,25(OH)_2_D^(^ ^56^ ^)^ so the increase in serum 1,25(OH)_2_D resulting from the *Ets1 cis* eQTL could enhance osteoclast differentiation through the Rank/RankL system to reduce bone mass. Second, ETS1 regulates *Runx2* gene transcription and this contributes to osteoblast proliferation and differentiation.^(^ ^57^, ^58^, ^59^ ^)^ *Ets1* mRNA levels were positively correlated to Tb.N. and ConnD, suggesting the B6 allele reduces ETS1 expression, which blunts osteoblast function and reduces bone mass.

The two loci with the greatest number of co‐localized basal phenotype QTL were F‐15C (9 phenotypes, 3 in both diet groups) and F‐20C (7 phenotypes, 4 in both diet groups). As such, these two loci are some of the strongest bone regulatory loci we observed. For F‐20C there are no *cis* eQTL and three predicted deleterious nonsynonymous variants, including those affecting the *Vgll1* gene. Vgll1 is a member of a protein family whose function is to modify gene transcription by competing with Yes1 Associated Transcriptional Regulator (YAP) transcription factors for binding to TEA domain transcription factors (TEADs).^(^ ^60^ ^)^ In bone, Yap1 drives osteoclastogenesis^(^ ^61^ ^)^ and works with Snail/Slug and Taz to control bone marrow stromal cell self‐renewal.^(^ ^62^ ^)^ Although research shows that Vgll4 can promote osteoblast differentiation by antagonizing TEADs‐mediated inhibition of Runx2 transcription,^(^ ^63^ ^)^ no research has examined the role of Vgll1 in bone cell biology. For F‐15C there are no *cis* eQTL and only one nonsynonymous variant in the coding region for the *Fam135b* gene. The *Fam135b* gene is constitutively expressed across tissues/cells and encodes a protein that promotes growth, migration, and invasion of cancer cells.^(^ ^64^ ^)^ However, Fam135b has not been studied in the context of bone biology. The strong impact of the F‐15C and F‐20C loci on bone, and the modest experimental links of the candidate genes to bone biology, suggest that follow‐up studies on these loci will reveal new aspects of bone biology.

The second set of candidate genes we identified includes those from loci F‐8C and F‐18C that determine the response of bone phenotypes to dietary Ca restriction (ie, the RCR phenotype). Within F‐8C (controlling the RCR for BMD, BMC, Tb.N, Tb.Sp, SMI, and Tt.Ar), we identified a variant causing a deleterious nonsynonymous amino acid change in the macrophage scavenger receptor 1 (*Msr1*) gene (B6 = asparagine; DBA = histidine). *Msr1* expression is high in osteoclasts, preosteoclast‐like RAW264.7 cells, and mature osteoblasts.^(^ ^65^ ^)^ *Msr1* knockout mice have fewer multinucleated osteoclasts, reduced bone resorption, and greater BMD.^(^ ^66^, ^67^ ^)^ Activation of Msr1 by acetylated low‐density lipoprotein (LDL) also promotes osteoclast differentiation by inducing RANK expression^(^ ^66^ ^)^ through the production and secretion of pro‐osteogenic cytokines by M2‐like macrophages.^(^ ^68^ ^)^ We hypothesize that the DBA allele in the *Msr1* gene encodes a less functional Msr1 protein as compared to the B6 allele. As a result, under low dietary Ca intake this less functional Msr1 protein would protect bone by blunting osteoclastic differentiation in response to pro‐resorbing signals.

The F‐18C locus controls the RCR phenotypes for the cortical bone phenotypes Imax/Cmax, *J*, Tt.Ar, and Ct.Ar, as well as the trabecular bone phenotypes SMI and Tb.Th. We identified polymorphisms affecting two promising candidate genes: one causing an arginine (B6) to leucine (DBA) substitution at amino acid residue 1895 within an unstructured region of adenomatosis polyposis coli (*Apc*) and an eQTL controlling *Camk4* mRNA levels. Apc is a negative regulator of the pro‐proliferative Wnt signaling that is critical for skeletal development in mice^(^ ^69^, ^70^ ^)^ and humans.^(^ ^71^ ^)^ In addition, human genetic studies have reported an association between variants in *Apc* and femur or spine BMD.^(^ ^72^, ^73^ ^)^ Because the B6 allele was associated with the ability to retain more bone under low dietary Ca intake, we hypothesize that the B6 allele in the *Apc* gene encodes a hypofunctional protein that protects bone by promoting osteoblast expansion and differentiation. Camk4 is a serine‐threonine protein kinase that is activated by increased intracellular Ca^(^ ^74^ ^)^ and that contributes to RANKL‐induced osteoclastogenesis and bone loss.^(^ ^75^ ^)^ We found that the Camk4 *cis* eQTL is driven by the DBA allele and Camk4 mRNA levels are negatively correlated with the RCR for bone mass/strength parameters (eg, Tb.Th, Ct.Ar, Tt.Ar). This suggests higher Camk4 mRNA levels may mediate an osteoclast driven increase in bone loss during dietary Ca restriction.

Several groups have previously used BXD mice to study the genetics controlling femur bone mass^(^ ^8^, ^13^ ^)^ or bone strength.^(^ ^43^ ^)^ The largest of these studies was conducted by Lu and colleagues^(^ ^13^ ^)^ who used 61 BXD RI lines in a mapping study to identify QTL for femoral and tibial μCT phenotypes in female and male mice aged 50 to 375 days. In contrast to our findings, Lu and colleagues^(^ ^13^ ^)^ did not find any QTL in males. We believe several factors make our study more reliable than the Lu and colleagues^(^ ^13^ ^)^ study, including: (i) strict control of environmental conditions and diet, (ii) careful balancing of RI lines across mouse shipments, (iii) use of a body size correction^(^ ^26^ ^)^ to account for size differences among the BXD lines, (iv) better line estimates due to more replicates within lines (mean 7.72 ± 0.78, median = 8 versus mean [males] 4.64 ± 2.67, median = 4), and (v) use of a very narrow harvest age window (mean = 85 days; range 81–91 days versus mean [males] = 96 days; range 50–375 days). Although Lu and colleagues^(^ ^13^ ^)^ attempted to statistically adjust for age in their analysis, trabecular bone mass (BV/TV) in mouse long bones increases 30% from 2 to 4 months of age and then drops by more than 60% by 12 months of age,^(^ ^76^ ^)^ so this correction may not have been effective.

Although we believe these differences, and the ability to replicate some robust QTL in two different dietary groups, makes our study stronger than other published BXD studies, some limitations should be noted. First, the BXD RI panel was derived from only two founders so it captures only ~20% of the genetic diversity that exists in the mouse genome.^(^ ^22^ ^)^ Second, though we used a large number of RI lines, the QTL intervals from our analysis are still relatively wide and encompass 100–200 genes. Finally, we studied only male mice so our study may not capture loci present in females. In fact, only the F‐12B QTL from our male mice (interval: 16.03–28.06 Mb, controlling Imax/Cmax and *J*) overlapped with any the 16 loci previously reported in BXD mice for female bone μCT traits (*Fcvf12*, interval: 15.0–27.0 Mb, controlling femur cortical volume).^(^ ^13^ ^)^ A recent study by Al‐Barghouthi and colleagues^(^ ^38^ ^)^ using diversity outbred (DO) mice overcomes some of these weaknesses by capturing >90% of genetic diversity in the mouse genome, having more narrow linkage blocks, and using male and female mice. However, this study only evaluated basal bone phenotypes and could not put isogenic mice in two dietary environments (ie, to reveal RCR traits). Thus, our study is complementary to the study by Al‐Barghouthi and colleagues.^(^ ^38^ ^)^

In summary, our study provides a unique view of the genetics controlling femur phenotypes. In addition to the loci we identify that were replicated under two dietary conditions, our study is the first to examine genetic variation controlling the response of bone mass and microarchitecture to the environmental stress of low dietary Ca intake. Our study confirmed the existence of G×D interactions influencing whole bone, Ct, and Tb phenotypes. Importantly, our mapping experiment revealed that the genetic variation controlling the RCR phenotypes is independent from that controlling basal phenotypes. This has expanded our understanding of the genetic regulation on femur phenotypes, particularly during low Ca intake. By coupling our QTL analysis with bioinformatics characterization, we uncovered several novel candidate genes for bone phenotypes, including several that are unique for the bone RCR. The genetic loci and candidate genes identified in this study serve as a foundation for future research to identify novel pathways and genes underlying the development of bone as well as the adaptation of bone to Ca insufficiency.

## Conflicts of interest

The authors have no conflicts to declare.

## Author contributions

**Krittikan Chanpaisaeng:** Data curation; formal analysis; investigation; validation; visualization; writing – original draft; writing – review and editing. **Perla C. Reyes‐Fernandez:** Data curation; investigation; writing – review and editing. **Brian Dilkes:** Formal analysis; validation; visualization; writing – original draft; writing – review and editing. **James C. Fleet:** Conceptualization; data curation; funding acquisition; investigation; project administration; validation; visualization; writing – original draft; writing – review and editing.

### Peer review

The peer review history for this article is available at https://publons.com/publon/10.1002/jbm4.10668.

## Supporting information


**Fig. S1.** Scout scans of mouse femur showing the region of interest for quantification in the proximal femur (left image, for trabecular bone) and femur midshaft (right image, for cortical bone).
**Fig. S2.** QTL plots for femur BMD, BMC, microCT phenotypes, and for basal body weight and femur length. Values in parentheses are the transformations and the covariate corrections used for the phenotype.
**Fig. S3.** QTL plots for high priority loci characterized by overlapping phenotype loci suggest pleiotropy.

**Table S1.** BXD Recombinant Inbred Mouse Lines and Number of Mice Used for Analysis
**Table S2.** Raw Line Means for Femoral Trabecular and Cortical Bone Traits and Their Responses to Ca Restriction (RCR) in 51 BXD RI Strains and Their Parental Strains
**Table S3.** Transformation and Covariate Correction for Each Femoral Bone Trait
**Table S4.** Body Size‐Corrected and Transformed Line Means for Femoral Trabecular and Cortical Bone Traits and Their Responses to Ca Restriction (RCR) in 51 BXD RI Strains and Their Parental Strains
**Table S5.** QTLs Influencing Femoral Bone Traits in Male BXD RI Mice
**Table S6.** Pearson Correlation Coefficients of Body Size‐corrected Line Means of Femoral Bone Traits in Each Diet Group and in RCR.
**Table S7.** Heritability Estimates (h^2^) for Femoral Bone Traits for Each Dietary Environment and Under Calcium Restriction
**Table S8.** Summary of Polymorphisms in High‐priority Loci of Distal Femur
**Table S9.** Candidate Genes with Predicted Deleterious Amino Acid Change
**Table S10.** Cis eQTL under the High Priority Loci of Femur BXD QTL
**Table S11.** Evidence Supporting Potential Function of Select Candidate Genes.

## Data Availability

Unadjusted and covariate adjusted values for all phenotypes of all BXD RI lines have been provided Table S2 and S3. Individual animal level data are available upon request. BXD genotyping data is available at http://gn1.genenetwork.org/webqtl/main.py?FormID=sharinginfo&GN_AccessionId=600 InfoPageNameBXDGeno. QTL results are available in Table S5 and Fig. S2.
