# Supplementary material for: Diet X Gene Interactions Control Femoral Bone Adaptation to Low Dietary Calcium
Source: JBMR Plus. 2022 Aug 19;6(9):e10668. doi: 10.1002/jbm4.10668 (PMC9465001; doi:10.1002/jbm4.10668)
Supplement: Supplementary file 1 — Fig. S1. Scout scans of mouse femur showing the region of interest for quantification in the proximal femur (left image, for trabecular bone) and femur midshaft (right image, for cortical bone). Fig. S2. QTL plots for femur BMD, BMC, microCT phenotypes, and for basal body weight and femur length. Values in parentheses are the transformations and the covariate corrections used for the phenotype. Fig. S3. QTL plots for high priority loci characterized by overlapping phenotype loci suggest pleiotropy. [file JBM4-6-e10668-s001.docx]

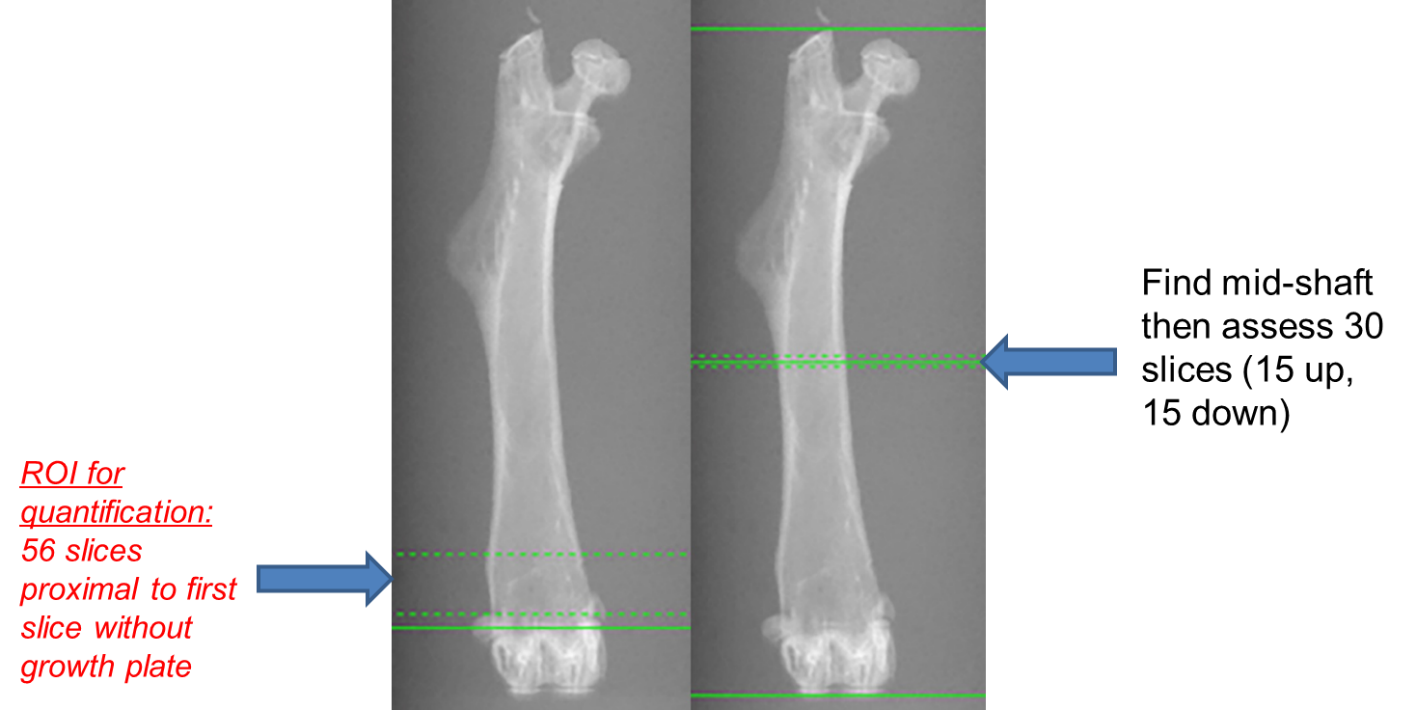


**Supplemental Figure S1.** Scout scans of mouse femur showing the region of interest for quantification in the proximal femur (left image, for trabecular bone) and femur midshaft (right image, for cortical bone).

**A. FL** (0.5% Ca group, unadjusted) **BW**
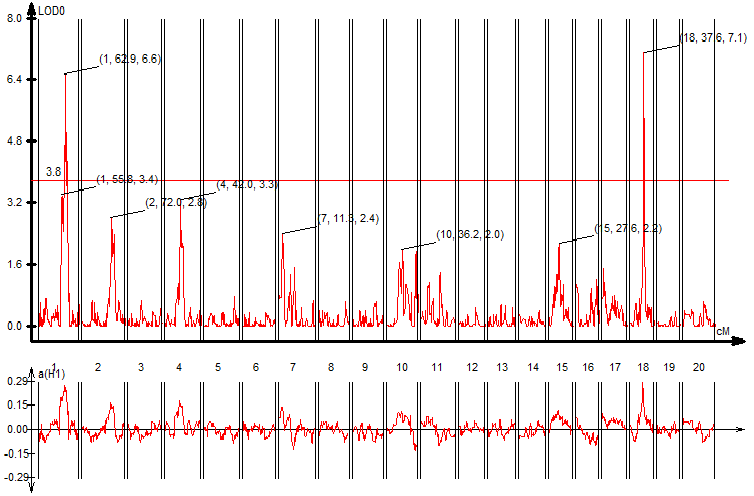
 (0.5% Ca group, unadjusted)


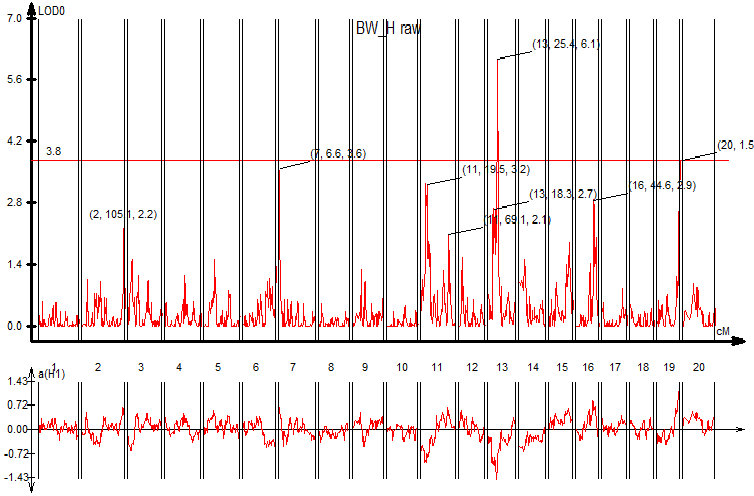


**B. BMD**

0.5% Ca (Log; BW, FL) 0.25% Ca (Log; BW, FL) RCR (none; BW, FL)

**
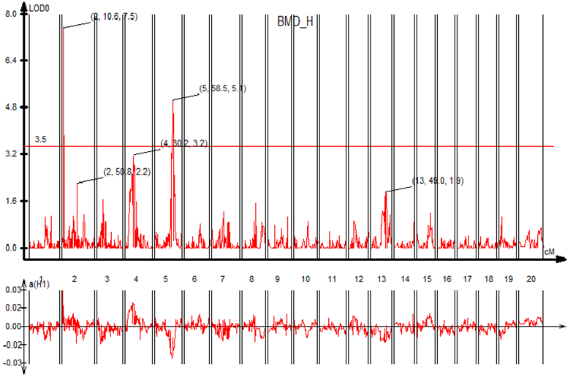

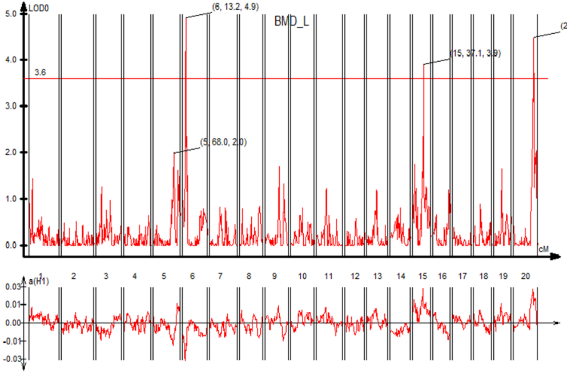

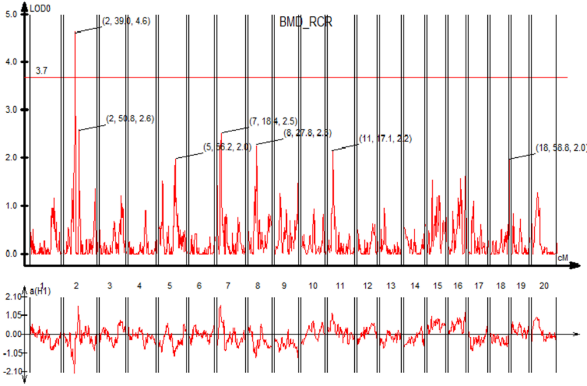
**

**C. BMC**

0.5% Ca (Log; BW, FL) 0.25% Ca (Log; BW, FL) RCR (none; BW, FL)

**
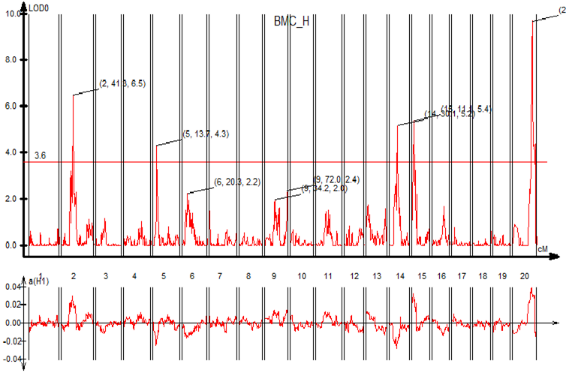

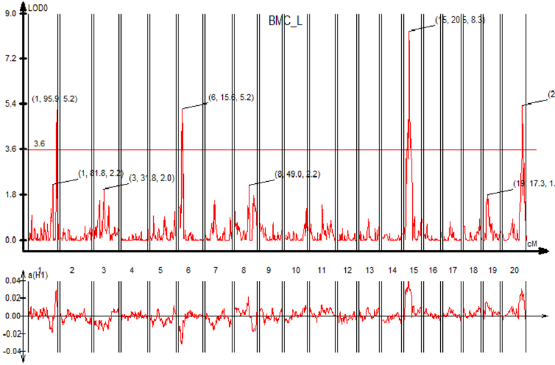

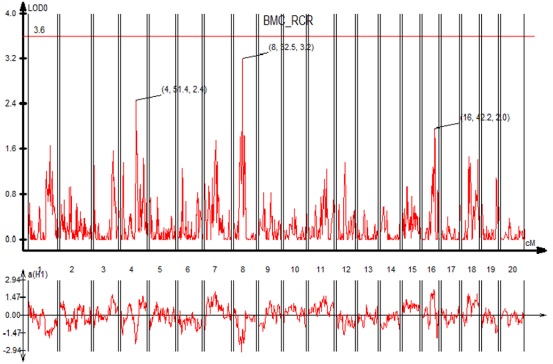
**

**D. Ct.Ar/Tt.Ar**

0.5% Ca (none; BW, FL) 0.25% Ca (none; BW, FL) RCR (none; BW, FL)


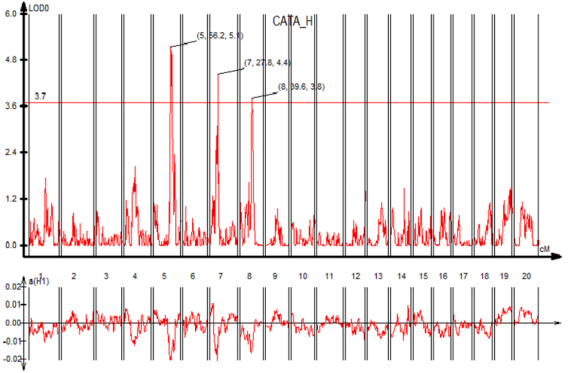

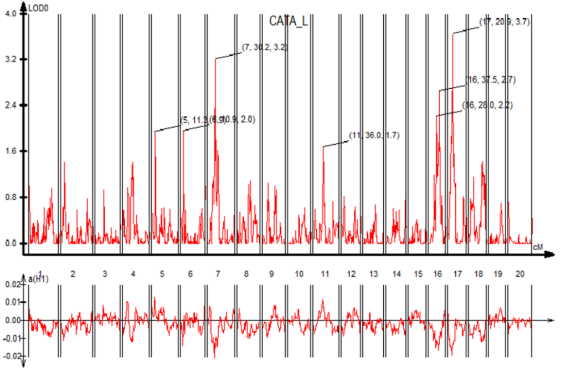

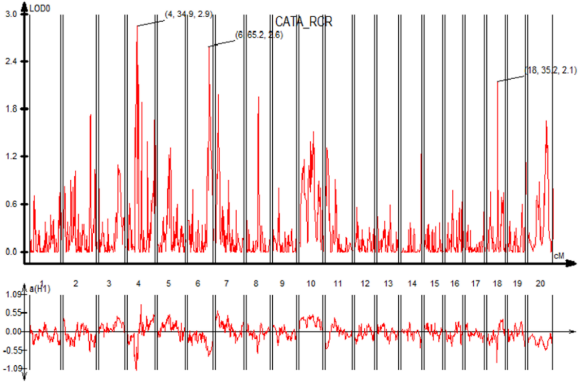


**E. Ct.Th**

0.5% Ca (none; BW, FL) 0.25% Ca (none; BW, FL) RCR (none; BW, FL)


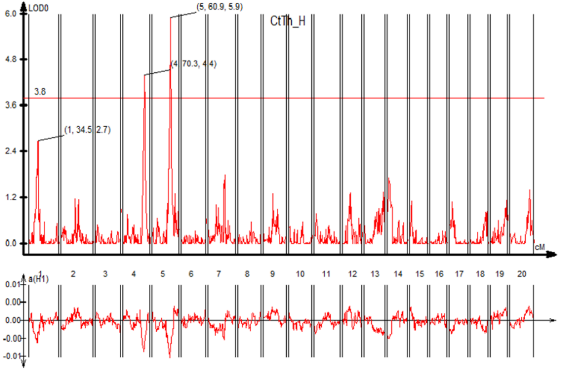

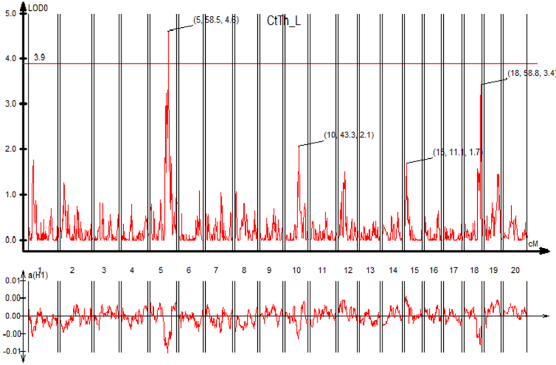

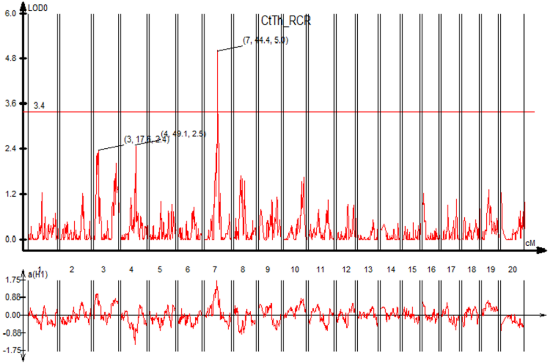


**F. Ct.Ar**

0.5% Ca (none; BW, FL) 0.25% Ca (none; BW, FL) RCR (none; BW, FL)


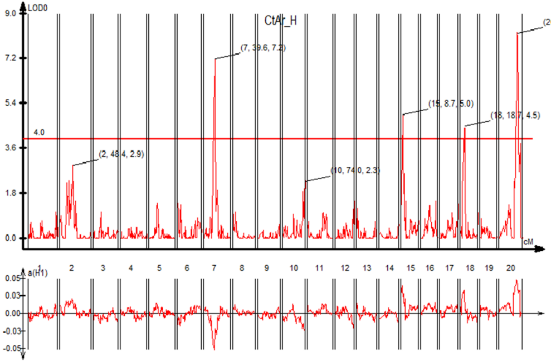

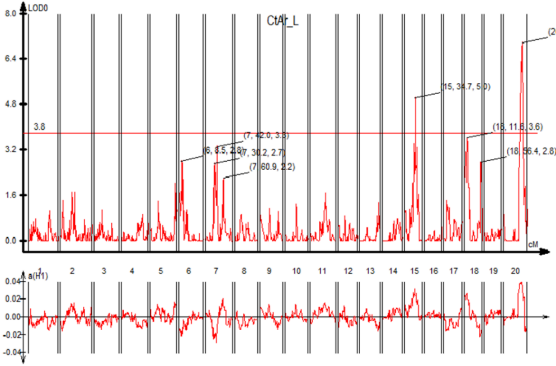

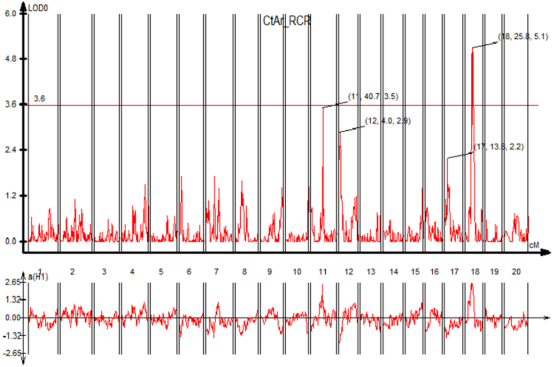


**G. Tt.Ar**

0.5% Ca (none; BW, FL) 0.25% Ca (none; BW, FL) RCR (none; BW, FL)


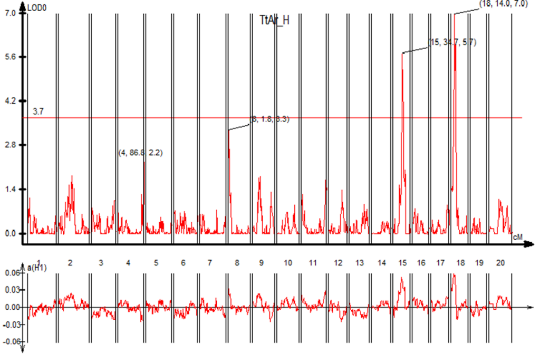

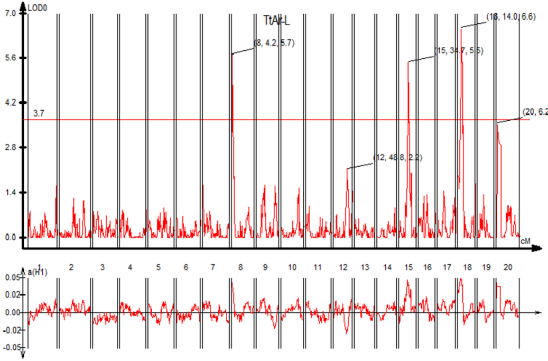

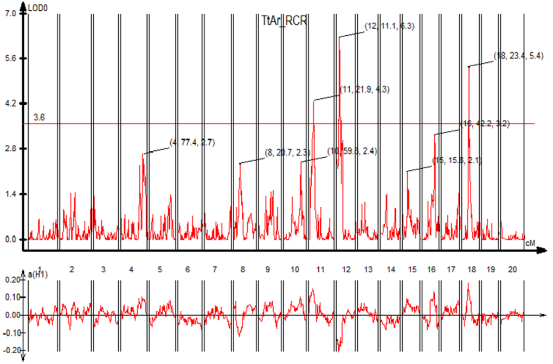


**H. MOI**

0.5% Ca (Log; BW, FL) 0.25% Ca (Log; BW, FL) RCR (SQRT(y+25); BW, FL)


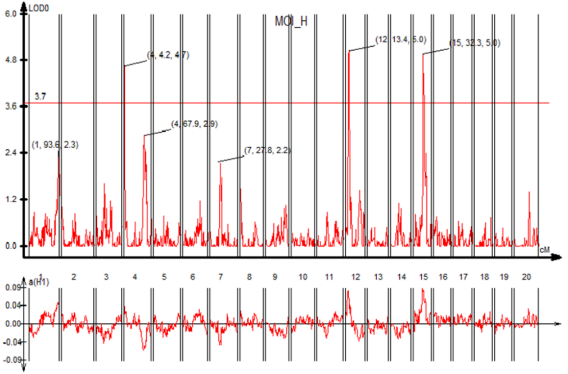

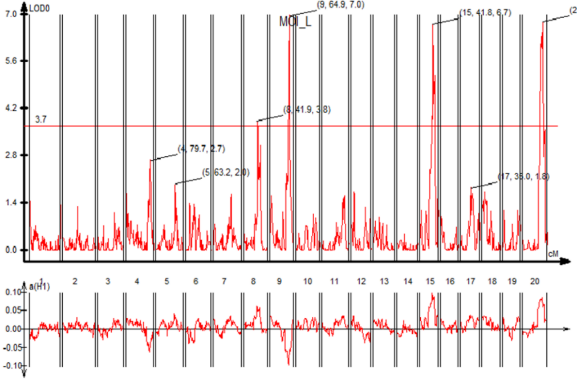

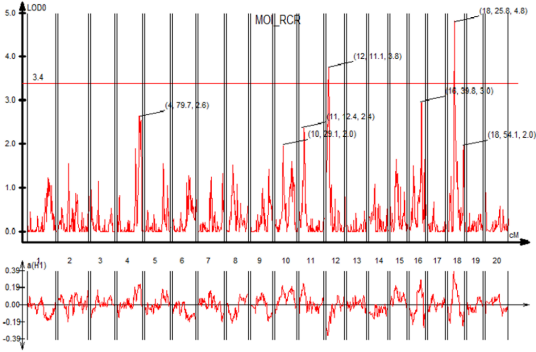


**I. Imax/Cmax**

0.5% Ca (Log; BW, FL) 0.25% Ca (Log; BW, FL) RCR (SQRT(y+30); BW, FL)


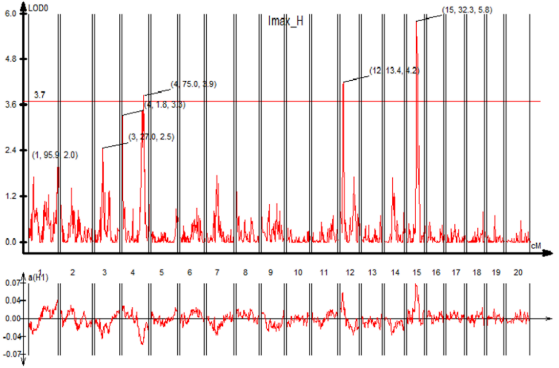

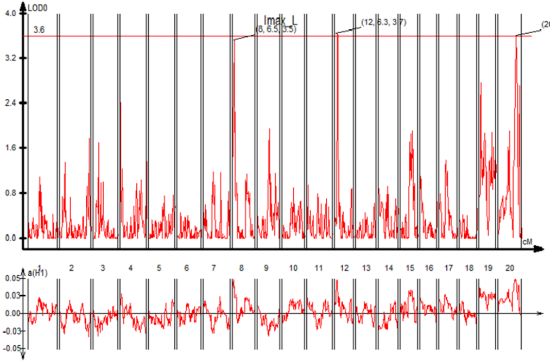

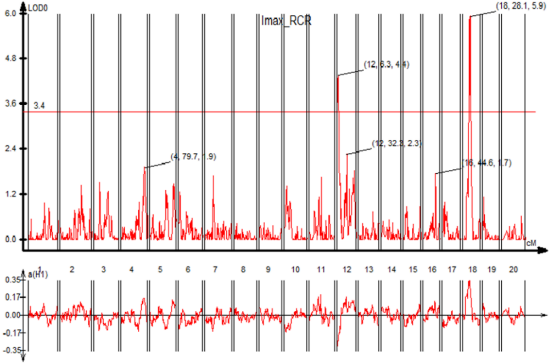


**J. Imin/Cmin**

0.5% Ca (Log; BW, FL) 0.25% Ca (Log; BW, FL) RCR (none; BW, FL)


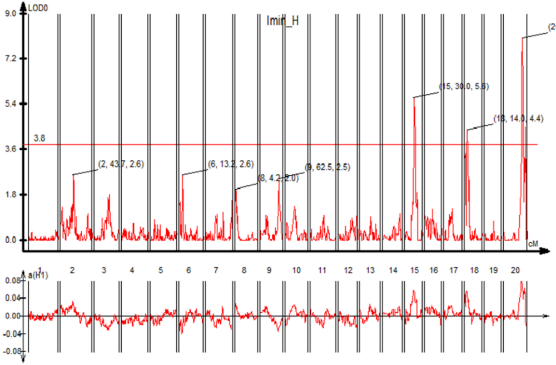

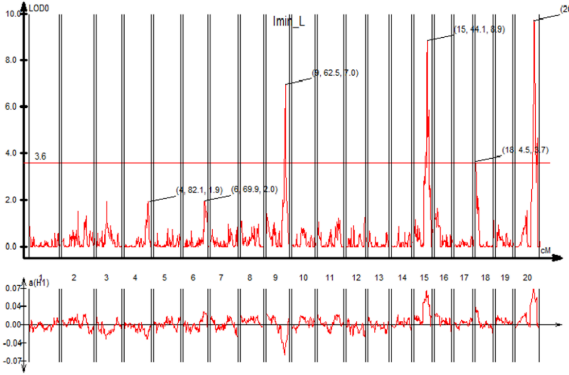

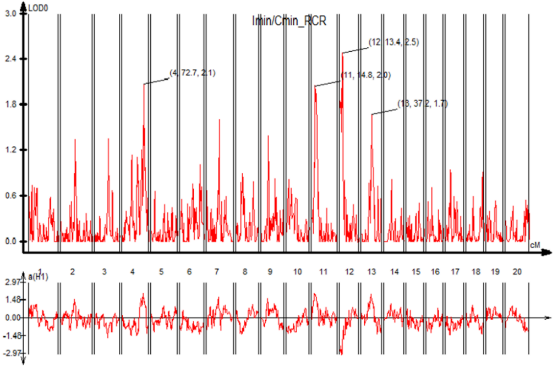


**K. BV/TV**

0.5% Ca (y^1/2^; BW, FL) 0.25% Ca (y^1/2^; BW, FL) RCR (y^1/3^+25); BW, FL)


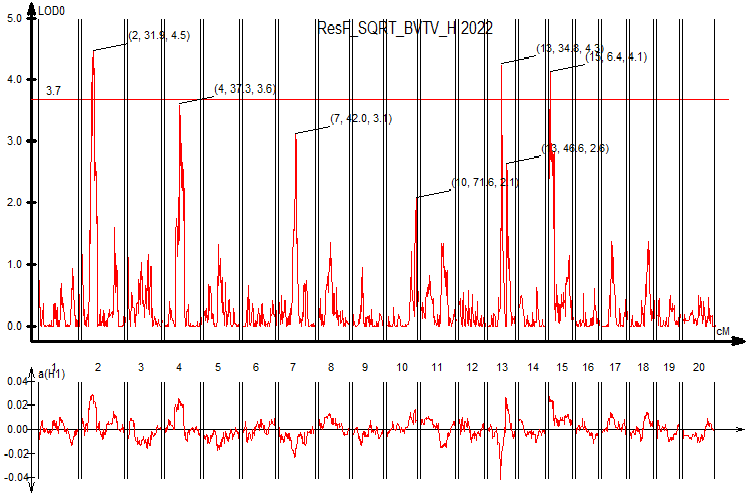

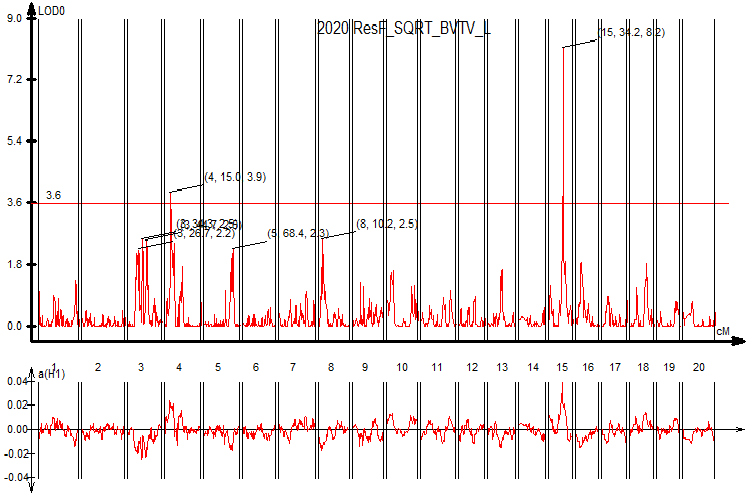

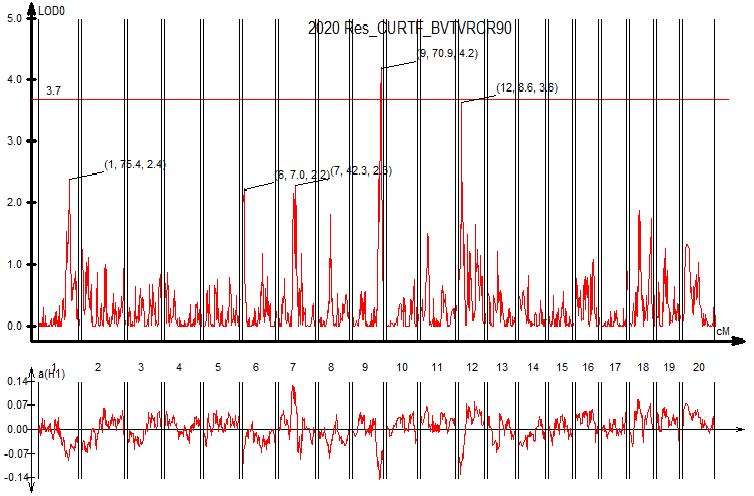


**L. Tb.Th**

0.5% Ca (Log; BW, FL) 0.25% Ca (Log; BW, FL) RCR (y^1/2^+35; BW, FL)


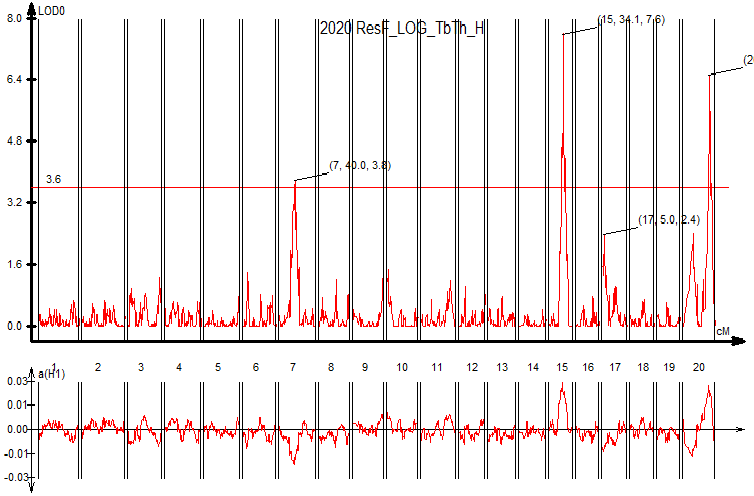

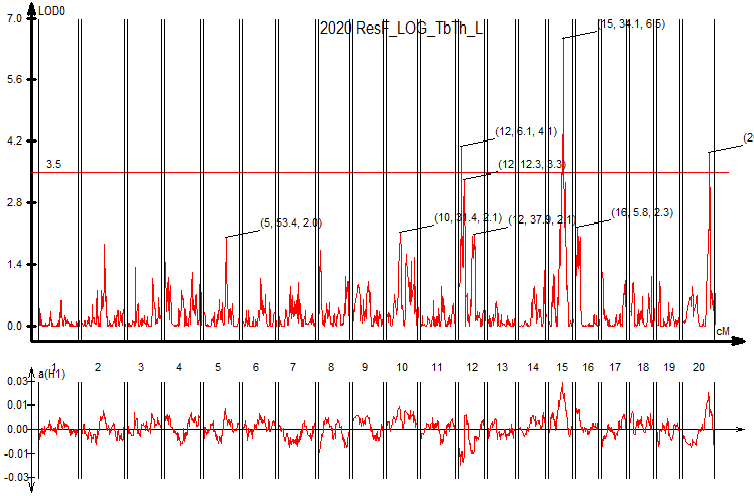

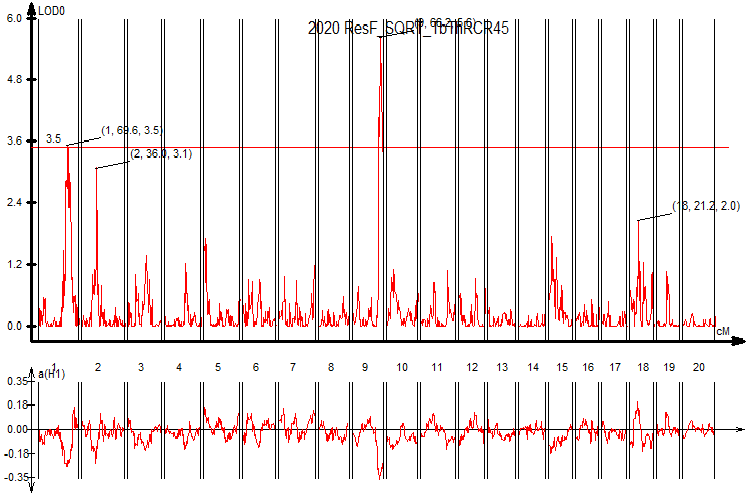


**M. Tb.N**

0.5% Ca (none; BW) 0.25% Ca (none; BW) RCR (none; FL)


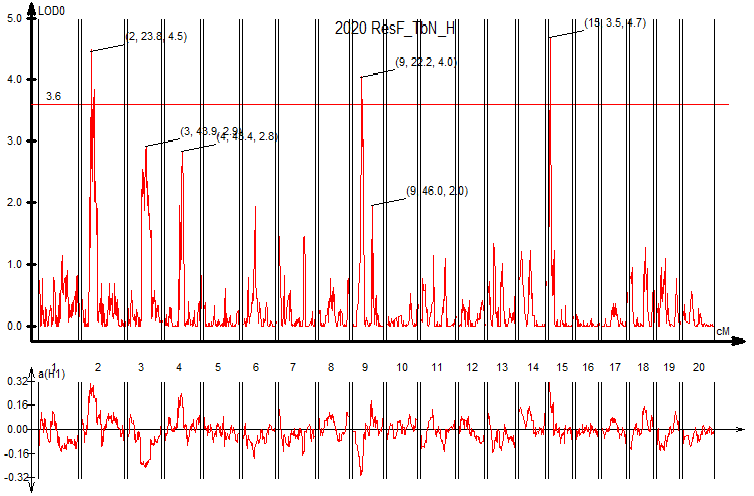

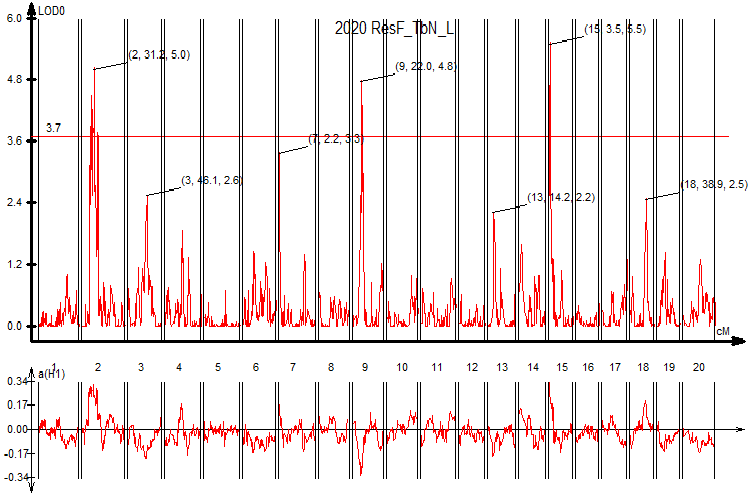

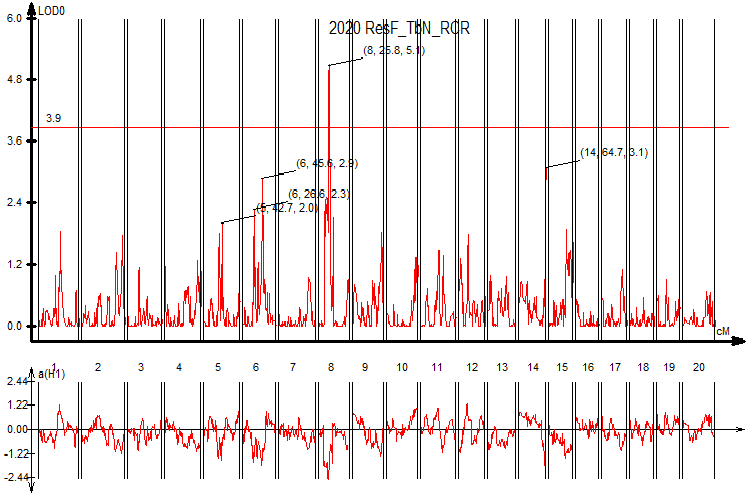


**N. Tb.Sp**

0.5% Ca (Log; BW) 0.25% Ca (Log; BW) RCR (none; FL)


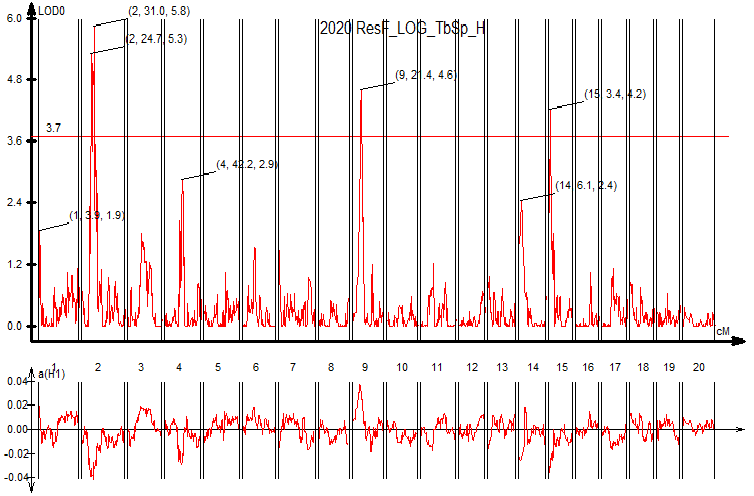

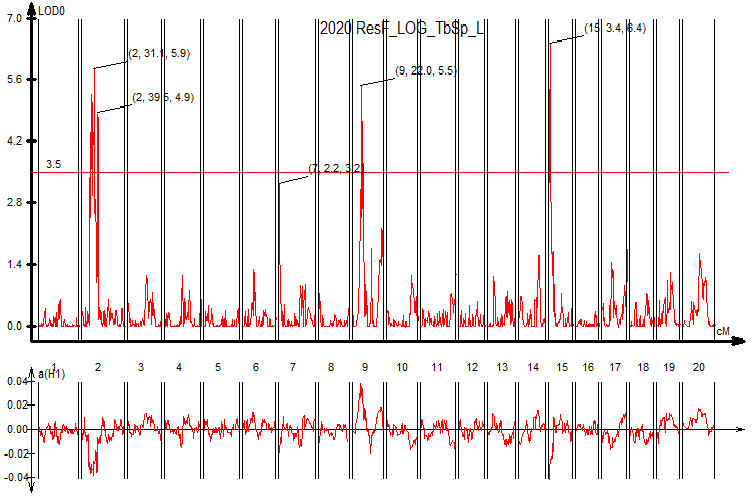

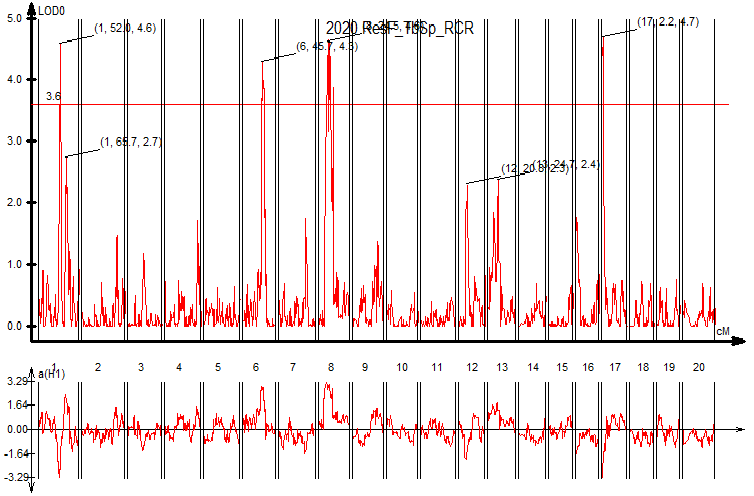


**O. SMI**

0.5% Ca (none; BW, FL) 0.25% Ca (none; BW, FL) RCR (y^1/2^+100; BW, FL)


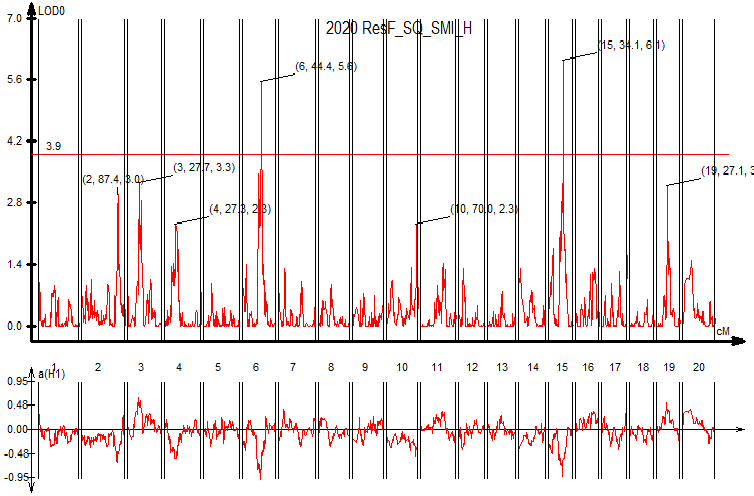

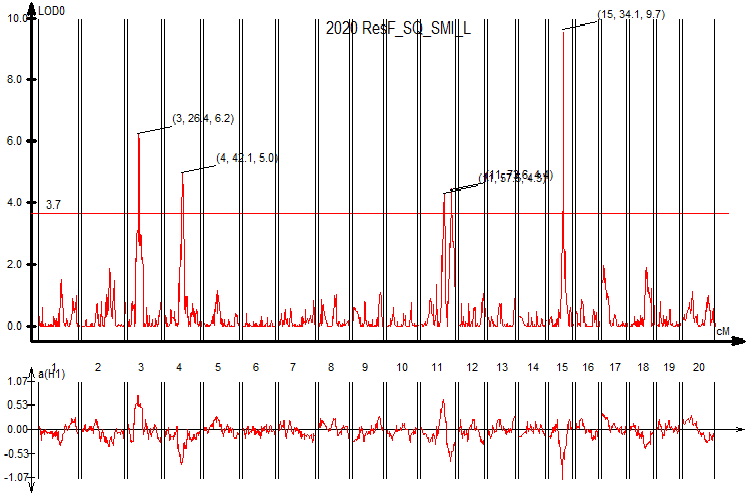

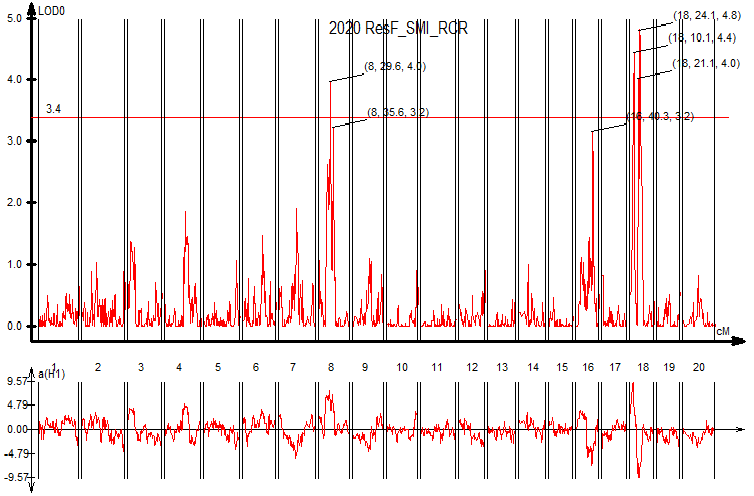


**P. Conn.D**

0.5% Ca (y^1/2^; none) 0.25% Ca (y^1/2^; none) RCR (none; FL)


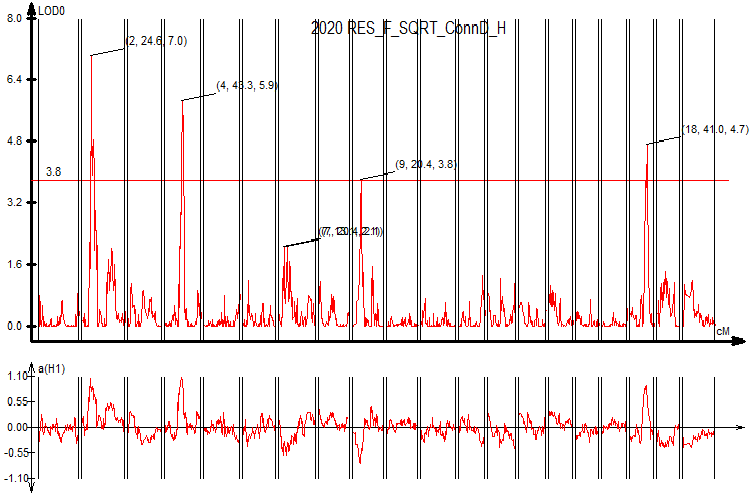

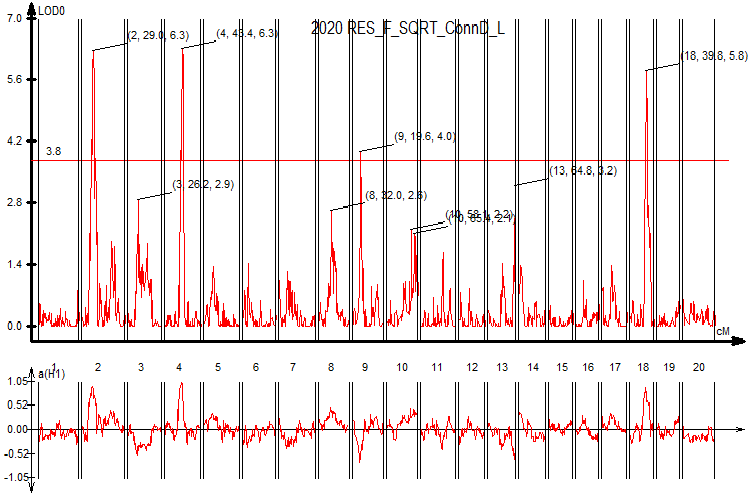

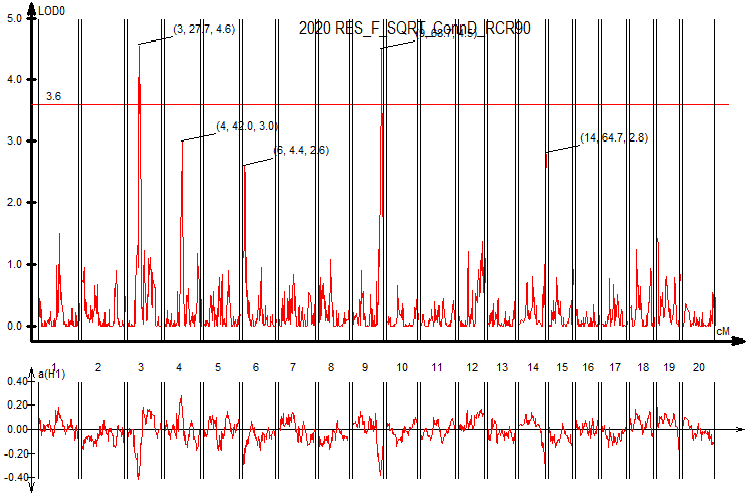


**Q. Tb.TMD**

0.5% Ca (none; none) 0.25% Ca (none, none) RCR (none, none)


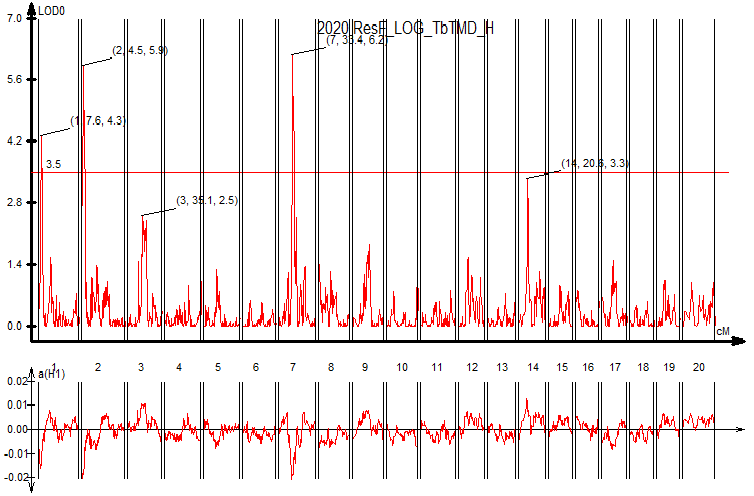

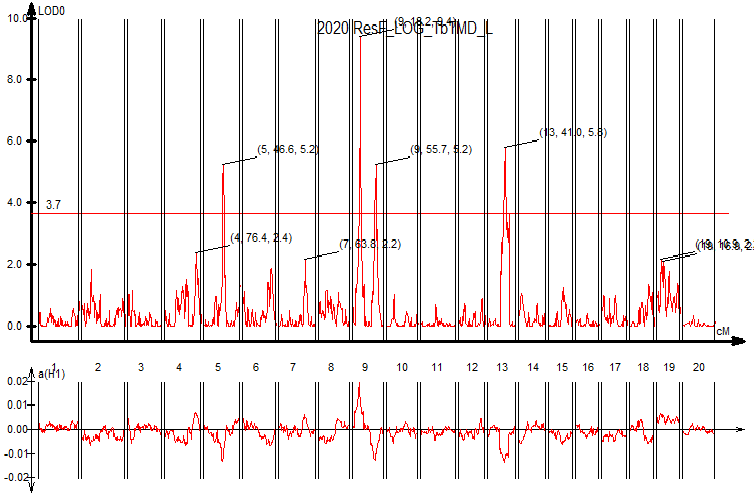

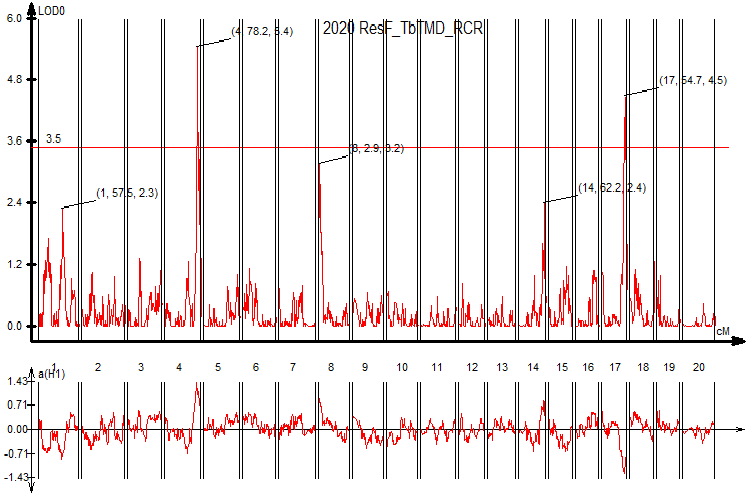


**Supplemental Figure S2.** QTL plots for femur BMD, BMC, microCT phenotypes, and for basal body weight and femur length. Values in parentheses are the transformations and the covariate corrections used for the phenotype.


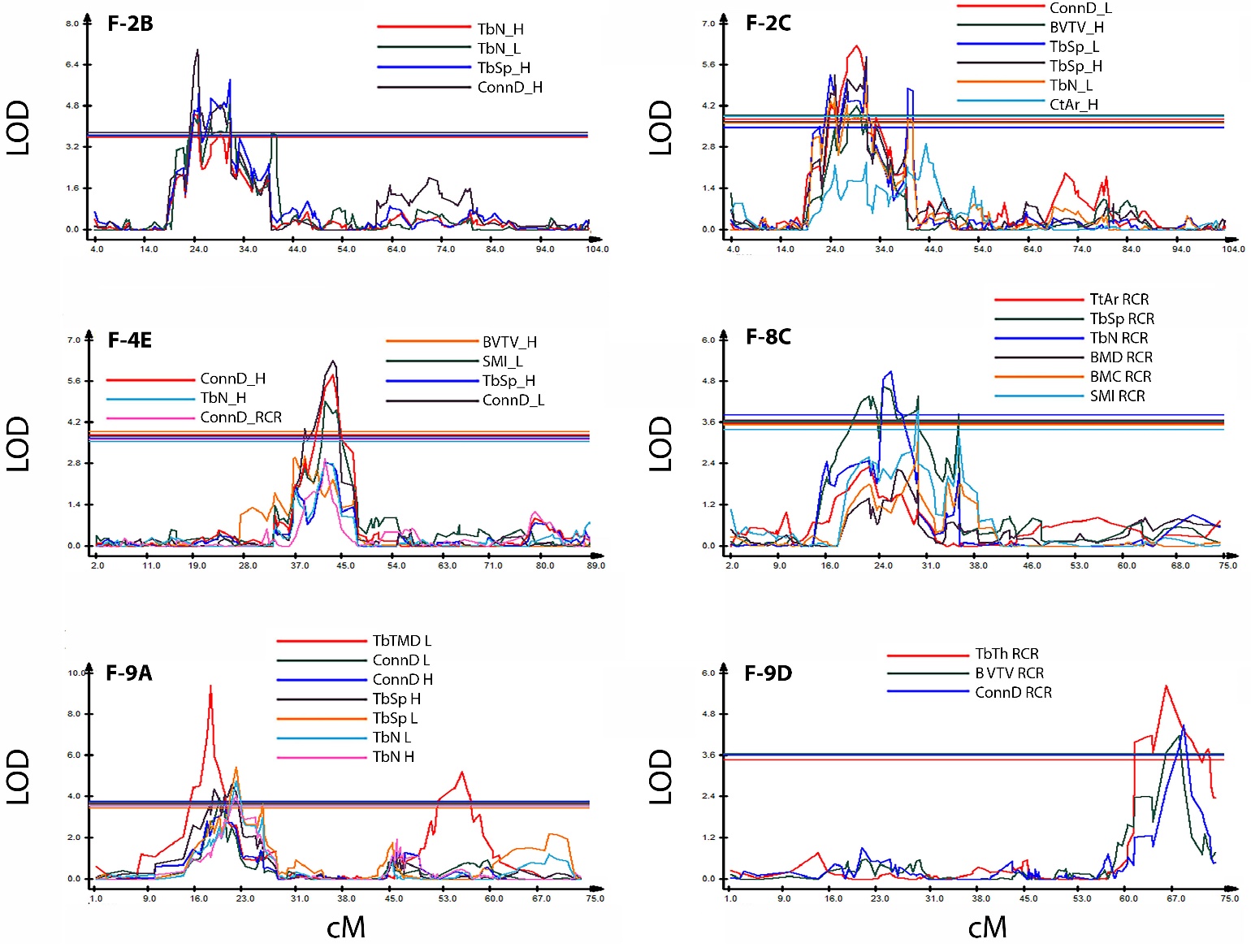


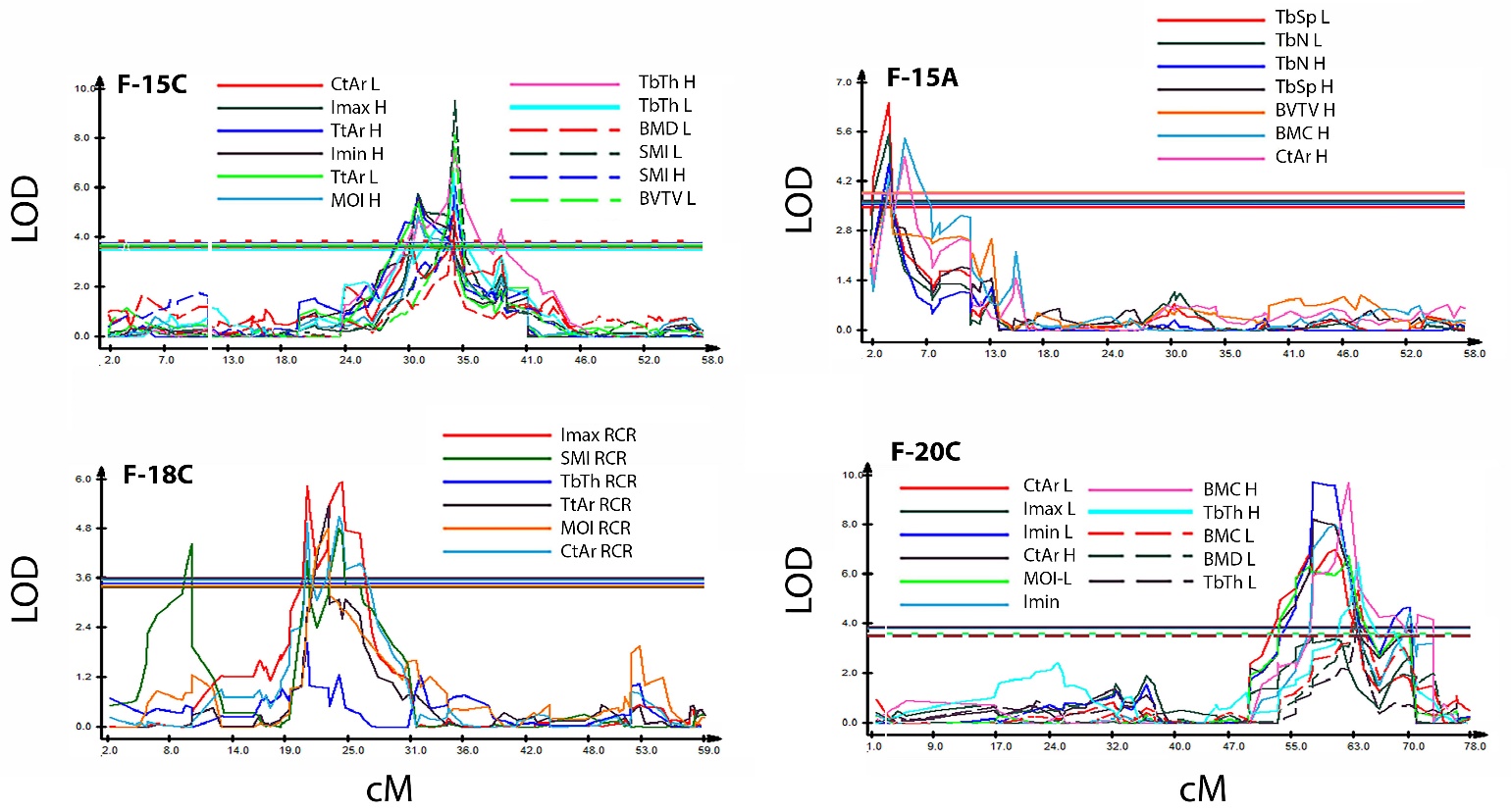
**Supplemental Figure S3.** QTL plots for high priority loci characterized by overlapping phenotype loci suggest pleiotropy.
