# Supplementary material for: Diet X Gene Interactions Control Femoral Bone Adaptation to Low Dietary Calcium
Source: JBMR Plus. 2022 Aug 19;6(9):e10668. doi: 10.1002/jbm4.10668 (PMC9465001; doi:10.1002/jbm4.10668)
Supplement: Supplementary file 2 — Table S1. BXD Recombinant Inbred Mouse Lines and Number of Mice Used for Analysis Table S2. Raw Line Means for Femoral Trabecular and Cortical Bone Traits and Their Responses to Ca Restriction (RCR) in 51 BXD RI Strains and Their Parental Strains Table S3. Transformation and Covariate Correction for Each Femoral Bone Trait Table S4. Body Size‐Corrected and Transformed Line Means for Femoral Trabecular and Cortical Bone Traits and Their Responses to Ca Restriction (RCR) in 51 BXD RI Strains and Their Parental Strains Table S5. QTLs Influencing Femoral Bone Traits in Male BXD RI Mice Table S6. Pearson Correlation Coefficients of Body Size‐corrected Line Means of Femoral Bone Traits in Each Diet Group and in RCR. Table S7. Heritability Estimates (h2) for Femoral Bone Traits for Each Dietary Environment and Under Calcium Restriction Table S8. Summary of Polymorphisms in High‐priority Loci of Distal Femur Table S9. Candidate Genes with Predicted Deleterious Amino Acid Change Table S10. Cis eQTL under the High Priority Loci of Femur BXD QTL Table S11. Evidence Supporting Potential Function of Select Candidate Genes. [file JBM4-6-e10668-s002.pdf]

Supplemental Table S1. BXD Recombinant Inbred Mouse Lines and Number of Mice Used for Analysis

Serial Number	BXD Line	JAX ID #	JAX 6-digit order #	Final # of mice included for anlaysis				Reasons for Exclusion
				# mice per line on the 0.5% Ca diet	# mice per line on the 0.25% Ca diet	Total # of mice per line	Exclusions	
1	BXD1	BXD1/TyJ	000036	8	8	16		
2	BXD2	BXD2/TyJ	000075	8	8	16		
3	BXD5	BXD5/TyJ	000037	8	8	16		
4	BXD6	BXD6/TyJ	000007	7	8	15	1	1 mouse with maloclussion (sacrificed before harvest)
5	BXD8	BXD8/TyJ	000084	6	7	13	3	2 mice died before harvest (dehydration); issue with water bottle. Both femurs of 1 mouse were broken during preparation.
6	BXD9	BXD9/TyJ	000105	8	8	16		
7	BXD11	BXD11/TyJ	000012	7	7	14	2	1 mouse died before harvest; 1 mouse sacrificed before harvest (dehydrated)
8	BXD12	BXD12/TyJ	000045	8	6	14	2	2 mice with encephalomegalia (sacrificed before harvest)
9	BXD13	BXD13/TyJ	000040	8	8	16		
10	BXD14	BXD14/TyJ	000329	7	8	15	1	1 mouse died before harvest
11	BXD15	BXD15/TyJ	000095	8	8	16		
12	BXD16	BXD16/TyJ	000013	8	8	16		
13	BXD18	BXD18/TyJ	000015	8	8	16		
14	BXD19	BXD19/TyJ	000010	8	8	16		
15	BXD20	BXD20/TyJ	000330	8	8	16		
16	BXD21	BXD21/TyJ	000077	8	7	15	1	Both femurs of 1 mouse were broken during preparation.
17	BXD22	BXD22/TyJ	000043	8	8	16		
18	BXD24	BXD24/TyJ-Cep290^{rd16}/J	000031	8	8	16		
19	BXD27	BXD27/TyJ	000041	8	8	16		
20	BXD28	BXD28/TyJ	000047	8	8	16		
21	BXD29	BXD29/TyJ	000029	8	8	16		
22	BXD31	BXD31/TyJ	000083	8	7	15	1	1 mouse died before harvest
23	BXD32	BXD32/TyJ	000078	8	8	16		
24	BXD33	BXD33/TyJ	003222	8	8	16		
25	BXD34	BXD34/TyJ	003223	9	7	16		
26	BXD36	BXD36/TyJ	003225	4	4	8		Only half of the mice were shipped from Jackson Labs, the rest of the order was cancelled
27	BXD38	BXD38/TyJ	003227	7	5	12	4	2 mice found dead before harvest; 2 mice were very weak, lost weight, died before harvest
28	BXD39	BXD39/TyJ	003228	8	8	16		
29	BXD40	BXD40/TyJ	003229	8	8	16		
30	BXD42	BXD42/TyJ	003230	7	8	15	1	Both femurs of 1 mouse were broken during preparation.
31	BXD44	BXD44/RwwJ	007094	8	8	16		
32	BXD48a (BXD96*)	BXD48a/RwwJ	007139	8	8	16		
33	BXD48	BXD48/RwwJ	007097	7	8	15	1	mouse had swollen prepuce area and infection (sacrificed before harvest)
34	BXD49	BXD49/RwwJ	007098	7	8	15	1	Both femurs of 1 mouse were broken during preparation.
35	BXD50	BXD50/RwwJ	007099	8	8	16		
36	BXD51	BXD51/RwwJ	007100	8	8	16		
37	BXD55	BXD55/RwwJ	007103	8	8	16		
38	BXD56	BXD56/RwwJ	007104	7	9	16		
39	BXD62	BXD62/RwwJ	007107	8	8	16		
40	BXD65a (BXD97*)	BXD65a/RwwJ	007140	8	7	15	1	Both femurs of 1 mouse were broken during preparation.
41	BXD68	BXD68/RwwJ	007113	8	8	16		
42	BXD69	BXD69/RwwJ	007114	8	8	16		
43	BXD73	BXD73/RwwJ	007117	8	8	16		
44	BXD75	BXD75/RwwJ	007119	8	8	16		
45	BXD84	BXD84/RwwJ	007127	7	8	15	1	Both femurs of 1 mouse were broken during preparation.
46	BXD86	BXD86/RwwJ	007129	8	8	16		
47	BXD87	BXD87/RwwJ	007130	8	8	16		
48	BXD89	BXD89/RwwJ	007132	8	9	17		
49	BXD90	BXD90/RwwJ	007133	8	8	16		
50	BXD98	BXD98/RwwJ	007141	7	8	15	1	Both femurs of 1 mouse were broken during preparation.
51	BXD100	BXD100/RwwJ	007143	8	7	15	1	mouse had scabs underneath fur towards posterior part of body, difficulty walking (sacrificed before harvest)

Total	**393**	**394**	**787**	**22**
Average	7.71	7.73	15.43	
SD	0.73	0.83	1.36	
Max	9.00	9.00	17.00	
Min	4.00	4.00	8.00	
Both Ave	7.72			
SD	0.78			
Median	8			
Max	9			
Min	4			

* Original BXD line number; This is used in figures

Supplemental Table S2. Raw Line Means for Femoral Trabecular and Cortical Bone Traits and Their Responses to Ca Restriction (

BXD line	bxd_line	BW Basal	BW LowCa	FL Basal	FL LowCa	BMD Basal	BMD LowCa	BMD RCR	BMC Basal	BMC LowCa	BMC RCR
1	1	23.70	24.10	15.30	15.37	0.0586	0.0588	0.4269	0.0227	0.0239	5.2226
2	2	25.92	26.33	15.02	14.94	0.0571	0.0555	-2.7920	0.0206	0.0206	0.3128
5	5	25.93	27.31	14.66	14.65	0.0546	0.0550	0.6674	0.0194	0.0207	6.4937
6	6	24.03	23.43	15.03	14.96	0.0613	0.0575	-6.1742	0.0239	0.0216	-9.5659
8	8	19.99	20.44	14.71	14.59	0.0609	0.0579	-4.9547	0.0229	0.0210	-8.5506
9	9	30.98	32.02	14.55	14.94	0.0538	0.0548	1.8355	0.0189	0.0203	7.5597
11	11	24.44	24.91	14.63	14.68	0.0652	0.0652	-0.0438	0.0248	0.0242	-2.4235
12	12	25.14	24.40	15.09	14.70	0.0683	0.0601	-11.9994	0.0267	0.0231	-13.6841
13	13	29.72	27.89	15.13	15.08	0.0544	0.0536	-1.5883	0.0202	0.0197	-2.2059
14	14	30.24	29.49	15.42	15.31	0.0628	0.0594	-5.5240	0.0251	0.0240	-4.4419
15	15	27.91	27.64	15.52	15.66	0.0643	0.0627	-2.5138	0.0267	0.0259	-2.9589
16	16	28.37	27.83	15.38	15.35	0.0651	0.0613	-5.8530	0.0270	0.0244	-9.4951
18	18	27.83	29.98	15.15	15.25	0.0609	0.0601	-1.2942	0.0229	0.0237	3.6026
19	19	24.53	23.98	15.66	15.64	0.0563	0.0524	-7.0160	0.0223	0.0212	-4.8794
20	20	25.75	24.65	15.37	14.85	0.0606	0.0565	-6.7437	0.0246	0.0210	-14.7074
21	21	27.12	27.50	15.45	15.58	0.0565	0.0564	-0.2654	0.0219	0.0216	-1.1422
22	22	23.10	22.65	14.33	14.27	0.0551	0.0541	-1.9501	0.0182	0.0182	-0.0687
24	24	25.47	26.28	14.63	14.61	0.0562	0.0551	-2.0245	0.0198	0.0188	-5.1136
27	27	24.46	25.03	14.50	14.59	0.0547	0.0516	-5.7789	0.0197	0.0188	-4.7589
28	28	30.53	29.05	14.80	14.63	0.0594	0.0542	-8.7907	0.0232	0.0216	-7.1121
29	29	22.33	22.05	15.01	14.81	0.0568	0.0520	-8.2945	0.0201	0.0182	-9.4085
31	31	27.92	27.79	14.85	14.92	0.0607	0.0605	-0.3060	0.0243	0.0242	-0.4115
32	32	23.15	22.23	14.45	14.36	0.0611	0.0565	-7.5153	0.0206	0.0188	-8.8345
33	33	23.64	23.35	14.75	14.60	0.0547	0.0512	-6.3528	0.0202	0.0185	-8.3591
34	34	23.63	23.98	15.09	15.09	0.0582	0.0534	-8.2177	0.0218	0.0195	-10.2650
36	36	25.93	27.40	15.49	15.55	0.0619	0.0589	-4.7927	0.0238	0.0236	-0.9804
38	38	24.53	24.13	15.61	15.27	0.0610	0.0533	-12.5983	0.0241	0.0191	-20.8407
39	39	29.13	31.06	14.67	14.74	0.0618	0.0583	-5.6634	0.0236	0.0223	-5.6584
40	40	21.98	22.00	15.29	15.20	0.0563	0.0562	-0.2664	0.0220	0.0216	-1.7634
42	42	22.49	22.01	15.33	15.33	0.0556	0.0550	-1.1816	0.0219	0.0215	-2.0847
44	44	31.04	31.29	15.62	15.69	0.0626	0.0620	-1.0383	0.0244	0.0246	0.9240
48	48	24.30	26.48	15.17	15.25	0.0588	0.0627	6.6586	0.0233	0.0259	11.0804
49	49	27.73	27.26	16.00	15.62	0.0635	0.0562	-11.4736	0.0276	0.0225	-18.6047
50	50	23.43	24.19	15.45	15.25	0.0608	0.0591	-2.7149	0.0225	0.0219	-2.9967
51	51	24.78	25.70	15.39	15.43	0.0618	0.0603	-2.4734	0.0237	0.0234	-1.3616
55	55	23.50	23.77	15.96	15.57	0.0599	0.0559	-6.6973	0.0247	0.0219	-11.0548
56	56	24.90	24.84	15.52	15.65	0.0557	0.0545	-2.0643	0.0222	0.0218	-2.0280
62	62	26.20	27.09	15.26	15.21	0.0612	0.0595	-2.8210	0.0239	0.0228	-4.2228
68	68	23.58	23.49	15.34	14.86	0.0561	0.0526	-6.1507	0.0219	0.0192	-12.4245
69	69	23.89	24.79	14.24	14.16	0.0553	0.0555	0.3615	0.0195	0.0185	-5.0129
73	73	25.15	25.21	15.41	15.42	0.0617	0.0624	1.0733	0.0239	0.0247	3.0303
75	75	24.87	23.23	15.20	15.29	0.0600	0.0552	-7.9524	0.0240	0.0204	-14.9583
84	84	24.28	23.36	14.83	15.03	0.0487	0.0471	-3.3111	0.0172	0.0168	-2.7858
86	86	29.89	29.54	15.13	15.10	0.0572	0.0559	-2.2727	0.0213	0.0200	-5.9377
87	87	19.04	20.28	14.07	14.18	0.0550	0.0570	3.7054	0.0179	0.0188	5.3147
89	89	23.33	23.41	15.07	15.04	0.0526	0.0507	-3.7019	0.0196	0.0181	-7.9426
90	90	25.95	26.50	15.84	16.02	0.0597	0.0586	-1.9263	0.0244	0.0234	-3.9939
96	96	25.48	26.00	15.60	15.45	0.0617	0.0567	-8.0900	0.0241	0.0225	-6.8876
97	97	29.58	28.78	15.54	15.79	0.0541	0.0532	-1.5723	0.0205	0.0217	5.4169
98	98	23.44	23.05	15.04	14.43	0.0551	0.0529	-4.0674	0.0200	0.0188	-6.2545
100	100	25.44	29.53	15.71	16.19	0.0624	0.0650	4.2522	0.0264	0.0301	13.9613

Abbreviation: BMD = bone mineral density (g/cm^2); BMC = bone mineral content (g); BV/TV = bone volume fraction; Tb.N = trabecular number (mm-1); Tb
SMI = structure model index; Tb.TMD = Trabecular tissue mineral density (mg of hydroxyappatite/cm^3); Ct.Ar = cortical bone area (mm^2); Tt.Ar = total cross-
J = polar moment of inertia (mm^4); *Imax* = maximum moment of inertia (mm^4); *Imin* = minimum moment of inertia (mm^4); RCR = the response to Ca restrict

(RCR) in 51 BXD RI Strains and Their Parental Strains

BV/TV Basal	BV/TV LowCa	BV/TV RCR	Tb.N Basal	Tb.N LowCa	Tb.N RCR	Tb.Th Basal	Tb.Th LowCa	Tb.Th RCR	Tb.Sp Basal
0.1222	0.1173	-4.0078	3.1818	3.0805	-3.1858	0.0736	0.0698	-5.1418	0.3191
0.2520	0.2077	-17.6074	5.2196	4.6406	-11.0912	0.0694	0.0673	-3.0074	0.1821
0.2557	0.2243	-12.2677	5.5789	4.9671	-10.9648	0.0667	0.0638	-4.4051	0.1692
0.3112	0.2035	-34.6050	6.0858	5.1313	-15.6832	0.0729	0.0650	-10.7853	0.1483
0.3083	0.2459	-20.2360	6.3792	5.9643	-6.5030	0.0682	0.0617	-9.6169	0.1421
0.1870	0.1560	-16.5619	4.7724	4.5681	-4.2815	0.0637	0.0614	-3.5918	0.2114
0.3606	0.3296	-8.6076	6.3689	6.3812	0.1939	0.0757	0.0727	-3.9811	0.1391
0.3549	0.2505	-29.4071	4.9689	4.7444	-4.5174	0.0995	0.0785	-21.0819	0.1793
0.1197	0.1035	-13.4890	4.4241	4.5431	2.6912	0.0565	0.0528	-6.5372	0.2280
0.2714	0.2090	-22.9680	5.2572	4.9664	-5.5332	0.0774	0.0705	-8.8649	0.1742
0.2042	0.1683	-17.5878	5.0584	4.7166	-6.7576	0.0697	0.0670	-3.8386	0.1868
0.2914	0.2257	-22.5344	5.3285	5.2486	-1.4997	0.0724	0.0645	-10.8444	0.1716
0.1794	0.1476	-17.7651	5.0830	4.7566	-6.4223	0.0609	0.0579	-5.0667	0.1928
0.1646	0.1162	-29.4252	4.4670	4.0362	-9.6439	0.0626	0.0572	-8.6210	0.2226
0.2549	0.1984	-22.1792	5.0886	4.5158	-11.2562	0.0753	0.0760	0.8532	0.1892
0.1414	0.1968	39.2420	4.5515	4.6631	2.4522	0.0604	0.0707	16.9936	0.2146
0.1795	0.1383	-22.9793	4.5333	4.4733	-1.3241	0.0663	0.0616	-7.1536	0.2179
0.1185	0.0899	-24.0828	3.8586	3.5302	-8.5112	0.0581	0.0608	4.7632	0.2590
0.1673	0.1499	-10.3915	4.4929	4.5585	1.4601	0.0652	0.0582	-10.7875	0.2264
0.2545	0.1674	-34.2337	6.1246	5.2232	-14.7181	0.0638	0.0581	-8.9485	0.1524
0.2130	0.1767	-17.0589	5.0688	4.9282	-2.7754	0.0669	0.0614	-8.2390	0.1918
0.1919	0.2383	24.2047	4.3519	4.0739	-6.3885	0.0720	0.0883	22.5713	0.2263
0.2737	0.1908	-30.2918	5.4849	4.8415	-11.7313	0.0721	0.0662	-8.0797	0.1644
0.2171	0.1804	-16.8913	5.9469	5.4729	-7.9701	0.0573	0.0555	-3.0786	0.1603
0.1570	0.1399	-10.9043	3.8423	3.6289	-5.5538	0.0679	0.0624	-8.0971	0.2614
0.1881	0.1627	-13.5150	5.1333	4.5366	-11.6242	0.0623	0.0614	-1.4446	0.1914
0.3174	0.2103	-33.7306	6.6850	6.0474	-9.5379	0.0658	0.0546	-17.0460	0.1372
0.3487	0.2806	-19.5311	6.3922	5.8741	-8.1063	0.0731	0.0677	-7.3909	0.1442
0.1249	0.1359	8.7661	4.5727	4.4293	-3.1360	0.0549	0.0581	5.9695	0.2171
0.1374	0.1485	8.0873	3.4678	3.4402	-0.7948	0.0677	0.0636	-6.0523	0.2961
0.2353	0.2329	-1.0221	5.3930	5.1674	-4.1837	0.0670	0.0647	-3.4108	0.1749
0.1904	0.2248	18.0869	4.9788	4.5635	-8.3419	0.0616	0.0696	13.0217	0.1930
0.3180	0.2104	-33.8395	5.7094	5.0299	-11.9008	0.0793	0.0677	-14.6907	0.1600
0.1990	0.2416	21.4081	5.4198	5.2967	-2.2706	0.0616	0.0698	13.3463	0.1783
0.1953	0.1834	-6.0984	5.1766	5.2469	1.3575	0.0612	0.0611	-0.1429	0.1829
0.2334	0.1547	-33.7067	4.8852	4.5284	-7.3037	0.0721	0.0650	-9.7798	0.1956
0.1759	0.1401	-20.3654	4.7390	4.6652	-1.5579	0.0613	0.0569	-7.1778	0.2057
0.2216	0.1969	-11.1406	4.8128	4.5026	-6.4455	0.0710	0.0688	-3.0645	0.1978
0.2008	0.1514	-24.6087	4.0806	3.6247	-11.1721	0.0754	0.0707	-6.3132	0.2364
0.2860	0.3020	5.5894	5.8396	5.9939	2.6427	0.0676	0.0676	-0.0211	0.1548
0.2904	0.3443	18.5388	5.6840	6.0681	6.7578	0.0772	0.0790	2.4465	0.1626
0.1370	0.1164	-15.0338	3.4490	3.4322	-0.4856	0.0725	0.0661	-8.8966	0.2985
0.1334	0.1191	-10.7097	4.2001	3.9178	-6.7221	0.0577	0.0563	-2.3805	0.2364
0.2660	0.2250	-15.4121	5.0507	4.8355	-4.2615	0.0750	0.0714	-4.7905	0.1866
0.1992	0.2423	21.6325	4.8444	4.6679	-3.6437	0.0659	0.0731	10.8126	0.2016
0.1291	0.0985	-23.6781	4.2321	3.6104	-14.6920	0.0604	0.0609	0.7960	0.2383
0.1951	0.1770	-9.2779	5.5049	5.3339	-3.1077	0.0602	0.0596	-0.9969	0.1740
0.2379	0.1397	-41.2581	4.7746	4.1387	-13.3190	0.0711	0.0583	-17.9916	0.1988
0.1302	0.1121	-13.9114	4.3677	4.0633	-6.9700	0.0569	0.0570	0.1977	0.2342
0.1830	0.1536	-16.0888	4.6700	4.1798	-10.4964	0.0631	0.0643	1.8920	0.2093
0.2768	0.2613	-5.5954	5.6237	5.3126	-5.5316	0.0700	0.0698	-0.1914	0.1700

.Th = trabecular thickness (mm); Tb.Sp = trabecular separation (mm); Conn.D = connectivity density (1/mm3);

-sectional area inside the periosteal envelope (mm^2); Ct.Ar/Tt.Ar = cortical area fraction;

ion.

Tb.Sp LowCa	Tb.Sp RCR	Conn.D Basal	Conn.D LowCa	Conn.D RCR	SMI Basal	SMI LowCa	SMI RCR	Tb.TMD Basal	Tb.TMD LowCa
0.3414	7.0002	38.4042	34.4276	-10.3547	2.7143	2.8047	3.3306	805.5780	807.1709
0.2068	13.5543	130.4448	102.3209	-21.5600	1.8818	2.0502	8.9516	761.6054	768.6971
0.1956	15.6112	155.5279	119.1818	-23.3695	1.9139	1.9784	3.3720	759.9769	753.9640
0.1881	26.8160	189.6922	121.7746	-35.8041	1.6425	2.3720	44.4180	757.0176	759.5056
0.1600	12.6037	206.8736	178.4510	-13.7391	1.6625	2.0795	25.0826	719.3990	704.2285
0.2131	0.8236	93.4895	81.8296	-12.4719	2.3158	2.5462	9.9501	747.2547	742.2916
0.1390	-0.0719	203.1136	201.4604	-0.8139	1.1754	1.3832	17.6793	759.1303	735.3180
0.1994	11.2056	82.7045	74.6848	-9.6968	1.0980	1.8706	70.3554	866.4006	793.7922
0.2118	-7.1393	61.0057	52.1473	-14.5206	2.9485	3.1004	5.1517	778.1909	774.1751
0.1895	8.7580	103.5533	97.0580	-6.2725	1.7172	2.1710	26.4299	798.5707	783.0689
0.2038	9.1291	97.3753	88.0112	-9.6165	2.5145	2.5749	2.4021	784.7021	789.9911
0.1808	5.3755	122.4462	116.8730	-4.5516	1.5281	2.0589	34.7322	789.3053	750.5056
0.2086	8.2145	102.9936	76.3764	-25.8436	2.5906	2.8229	8.9691	753.9514	752.3811
0.2436	9.4401	83.6322	55.8190	-33.2565	2.4808	2.7842	12.2317	742.9889	731.8624
0.2150	13.6212	121.8131	83.3946	-31.5389	1.8059	2.1825	20.8532	760.5454	815.8834
0.2092	-2.4793	75.7460	85.6439	13.0672	2.6081	2.4055	-7.7672	794.3929	795.6583
0.2214	1.6004	83.8183	71.0923	-15.1828	2.5396	2.5417	0.0827	792.9550	779.6591
0.2874	10.9642	53.3079	34.5436	-35.1999	2.9254	3.0691	4.9126	801.6098	798.7656
0.2075	-8.3556	85.9064	88.7012	3.2533	2.5139	2.6003	3.4349	771.3053	756.1230
0.1793	17.6506	175.7307	106.9894	-39.1174	2.0534	2.6686	29.9595	720.8516	747.3689
0.2001	4.3573	109.4806	92.9910	-15.0617	2.3182	2.4896	7.3901	749.0187	754.2496
0.2372	4.7992	70.4596	63.9092	-9.2966	2.2103	2.0702	-6.3415	803.2135	832.9623
0.2012	22.3297	133.6294	95.3397	-28.6536	1.8830	2.3844	26.6291	777.1556	783.6718
0.1763	9.9992	174.7707	139.8291	-19.9928	2.2733	2.4853	9.3258	724.2839	769.5081
0.2800	7.1265	62.9933	61.9859	-1.5991	2.4802	2.4485	-1.2760	799.3246	774.1225
0.2153	12.4869	117.2124	90.2652	-22.9901	2.5535	2.5281	-0.9957	749.8418	769.2713
0.1600	16.5962	230.7872	188.8255	-18.1820	1.4056	2.1976	56.3430	704.1634	722.0500
0.1606	11.3684	197.7352	171.8397	-13.0961	1.1855	1.6334	37.7801	720.9511	736.3129
0.2138	-1.5454	67.4457	71.0599	5.3587	2.9013	2.8302	-2.4494	738.5793	762.9781
0.3076	3.8796	61.8397	81.9854	32.5773	2.4477	2.2623	-7.5756	773.6919	755.4144
0.1834	4.8303	128.2786	115.0815	-10.2878	2.1048	2.0115	-4.4302	764.3293	785.2945
0.2132	10.4404	109.2577	87.2739	-20.1211	2.2353	1.9437	-13.0436	748.1007	771.9393
0.1936	20.9550	138.5503	102.8839	-25.7426	1.3797	2.1766	57.7557	771.2413	763.9341
0.1847	3.5960	128.9266	126.3075	-2.0314	2.5317	2.1223	-16.1690	764.1719	761.8925
0.1833	0.1884	114.6082	107.9055	-5.8483	2.3904	2.4698	3.3211	771.8976	781.3473
0.2147	9.7642	112.6613	78.7420	-30.1073	2.0314	2.4766	21.9126	803.6183	774.3999
0.2097	1.9537	87.5206	86.3575	-1.3290	2.4805	2.7869	12.3510	777.0409	750.8726
0.2169	9.6309	99.4393	86.1632	-13.3510	2.0973	2.2339	6.5094	784.6034	789.3573
0.2756	16.5824	77.9736	65.8484	-15.5504	2.1077	2.4078	14.2396	799.7050	784.8278
0.1560	0.7696	174.9690	189.2393	8.1559	1.6696	1.5172	-9.1290	736.4539	736.1579
0.1474	-9.3726	144.1644	163.9622	13.7328	1.3619	1.1774	-13.5498	762.8259	765.4983
0.2901	-2.8308	52.7466	35.5618	-32.5800	2.4894	2.7306	9.6918	817.1308	819.6504
0.2563	8.4033	71.7204	66.2528	-7.6236	2.6910	2.7173	0.9744	745.7558	764.8178
0.1984	6.3442	123.0041	110.9423	-9.8060	1.8451	2.1222	15.0180	779.3130	780.5940
0.2081	3.2462	100.4741	106.0535	5.5531	2.3326	1.7447	-25.2041	786.3101	801.7599
0.2815	18.1522	62.0152	38.7762	-37.4730	2.8333	2.8423	0.3165	774.0331	776.4598
0.1810	3.9793	127.5483	109.6652	-14.0206	2.4682	2.6255	6.3757	727.7949	758.1956
0.2391	20.2427	105.3619	66.9169	-36.4886	1.7990	2.3611	31.2500	767.2090	754.5720
0.2496	6.5809	80.7021	64.4108	-20.1869	2.8277	2.9294	3.5963	740.5379	761.1766
0.2369	13.1877	94.8832	73.5485	-22.4852	2.3805	2.5605	7.5602	754.5460	774.4459
0.1800	5.9141	162.0820	137.2640	-15.3120	1.7797	1.7641	-0.8741	733.7696	755.6920

Tb.TMD RCR	Ct.Ar Basal	Ct.Ar LowCa	Ct.Ar RCR	Tt.Ar Basal	Tt.Ar LowCa	Tt.Ar RCR	Ct.Ar/Tt.Ar Basal	Ct.Ar/Tt.Ar LowCa	Ct.Ar/Tt.Ar RCR
0.1977	1.0426	1.0355	-0.6851	2.0817	2.1007	0.9128	0.5041	0.4936	-2.0801
0.9312	0.8651	0.8845	2.2397	1.6385	1.6905	3.1725	0.5345	0.5104	-4.5138
-0.7912	0.8205	0.8229	0.2907	1.6946	1.7317	2.1889	0.4846	0.4746	-2.0635
0.3287	0.9676	0.9219	-4.7261	1.5931	1.5353	-3.6311	0.6079	0.5998	-1.3348
-2.1088	0.9521	0.9026	-5.1932	1.7455	1.7359	-0.5530	0.5453	0.5281	-3.1542
-0.6642	0.7552	0.7671	1.5672	1.3602	1.4226	4.5906	0.5543	0.5391	-2.7489
-3.1368	0.9298	0.9091	-2.2257	1.5972	1.5785	-1.1699	0.5814	0.5751	-1.0885
-8.3805	1.1247	1.0040	-10.7351	2.0411	1.9580	-4.0732	0.5507	0.5119	-7.0402
-0.5160	0.7999	0.7665	-4.1716	1.5040	1.4510	-3.5220	0.5325	0.5285	-0.7462
-1.9412	0.9742	0.9198	-5.5859	1.8990	1.7799	-6.2695	0.5256	0.5092	-3.1202
0.6740	1.0416	1.0106	-2.9776	1.7276	1.7241	-0.2019	0.6033	0.5955	-1.2867
-4.9157	1.1022	0.9956	-9.6775	1.9978	1.8977	-5.0124	0.5517	0.5243	-4.9641
-0.2083	1.0283	1.0247	-0.3567	1.7359	1.7200	-0.9159	0.6053	0.5960	-1.5364
-1.4975	0.9112	0.8602	-5.5961	1.9305	1.8985	-1.6588	0.4716	0.4532	-3.8967
7.2761	0.9202	0.8736	-5.0638	1.8053	1.7359	-3.8437	0.5102	0.5034	-1.3353
0.1593	0.9417	0.9956	5.7262	1.7192	1.8001	4.7075	0.5631	0.5528	-1.8288
-1.6768	0.8626	0.8448	-2.0573	1.5139	1.5411	1.8004	0.5613	0.5482	-2.3361
-0.3548	0.7731	0.7738	0.0821	1.1764	1.1894	1.1086	0.6576	0.6438	-2.1069
-1.9684	0.8143	0.7646	-6.0973	1.6716	1.6405	-1.8595	0.4869	0.4815	-1.1090
3.6786	1.0142	0.9370	-7.6149	1.9298	1.8963	-1.7367	0.5440	0.4950	-9.0063
0.6984	0.8443	0.8234	-2.4837	1.6184	1.6078	-0.6569	0.5284	0.5122	-3.0706
3.7037	1.0611	1.0376	-2.2150	1.9089	1.9093	0.0195	0.5555	0.5434	-2.1678
0.8385	0.9024	0.8182	-9.3266	1.4332	1.3124	-8.4304	0.6292	0.6154	-2.2024
6.2440	0.9160	0.8687	-5.1690	1.5670	1.5501	-1.0805	0.5845	0.5675	-2.8991
-3.1529	0.8382	0.7902	-5.7310	1.5510	1.5160	-2.2544	0.5352	0.5214	-2.5837
2.5911	0.9061	0.9493	4.7669	1.4768	1.5851	7.3304	0.6135	0.5985	-2.4369
2.5401	0.9049	0.8038	-11.1807	1.7369	1.6040	-7.6546	0.5214	0.5020	-3.7337
2.1308	0.9683	0.9277	-4.1977	1.8513	1.8234	-1.5048	0.5224	0.5082	-2.7252
3.3035	0.8427	0.8500	0.8655	1.6728	1.7317	3.5163	0.4946	0.4938	-0.1762
-2.3624	0.8905	0.8976	0.8057	1.8279	1.8466	1.0229	0.4884	0.4710	-3.5678
2.7430	0.9567	0.9647	0.8312	1.6223	1.6727	3.1047	0.5768	0.5768	0.0022
3.1866	0.9587	1.0098	5.3330	1.7987	1.9037	5.8383	0.5325	0.5314	-0.2009
-0.9475	1.1118	1.0177	-8.4697	2.1360	2.0766	-2.7797	0.5214	0.4900	-6.0246
-0.2983	0.8895	0.9039	1.6186	1.5650	1.6042	2.5049	0.5680	0.5634	-0.8033
1.2242	1.0069	1.0370	2.9860	1.7842	1.8699	4.8027	0.5645	0.5563	-1.4570
-3.6359	0.8900	0.8367	-5.9849	1.7261	1.6845	-2.4134	0.5151	0.4969	-3.5239
-3.3677	0.8462	0.8515	0.6238	1.6215	1.6276	0.3795	0.5221	0.5248	0.5216
0.6059	0.9968	0.9916	-0.5207	1.7807	1.7568	-1.3424	0.5601	0.5465	-2.4304
-1.8603	0.8840	0.8182	-7.4409	1.5604	1.4723	-5.6417	0.5592	0.5502	-1.6108
-0.0402	0.7995	0.8299	3.7927	1.4912	1.5322	2.7531	0.5327	0.5423	1.7997
0.3503	0.9154	0.9742	6.4238	1.7596	1.7047	-3.1171	0.5369	0.5709	6.3401
0.3084	1.0441	0.9616	-7.8997	1.8038	1.7524	-2.8462	0.5991	0.5702	-4.8097
2.5561	0.7432	0.7091	-4.5842	1.5744	1.5575	-1.0701	0.4724	0.4557	-3.5381
0.1644	0.8615	0.8304	-3.6081	1.3837	1.3943	0.7622	0.6066	0.5954	-1.8362
1.9648	0.8011	0.8681	8.3589	1.4548	1.5429	6.0599	0.5701	0.5678	-0.4009
0.3135	0.8361	0.7919	-5.2915	1.5280	1.5130	-0.9850	0.5514	0.5256	-4.6839
4.1771	1.0623	1.0215	-3.8425	2.0717	2.0419	-1.4389	0.5228	0.5002	-4.3205
-1.6471	1.0341	0.9850	-4.7487	2.0105	2.0317	1.0561	0.5158	0.4851	-5.9613
2.7870	0.8423	0.8337	-1.0185	1.6581	1.6790	1.2598	0.5080	0.4962	-2.3382
2.6373	0.9039	0.8652	-4.2905	1.6552	1.6328	-1.3586	0.5462	0.5300	-2.9726
2.9876	1.0950	1.1956	9.1858	2.0073	2.2002	9.6097	0.5443	0.5436	-0.1316

Ct.Th Basal	Ct.Th LowCa	Ct.Th RCR	*J* Basal	*J* LowCa	*J* RCR	*Imax* Basal	*Imax* LowCa	*Imax* RCR	*Imin* Basal
0.2373	0.2340	-1.3699	0.5338	0.5363	0.4794	0.3459	0.3400	-1.7058	0.2641
0.2195	0.2060	-6.1503	0.3446	0.3654	6.0352	0.2545	0.2636	3.5536	0.1788
0.2054	0.2019	-1.7042	0.3475	0.3611	3.8977	0.2464	0.2500	1.4728	0.1876
0.2582	0.2466	-4.4706	0.3670	0.3473	-5.3589	0.2701	0.2534	-6.1800	0.1846
0.2403	0.2276	-5.2878	0.3986	0.3892	-2.3574	0.2883	0.2772	-3.8561	0.2048
0.2173	0.2133	-1.8412	0.2494	0.2681	7.5042	0.2089	0.2172	3.9846	0.1379
0.2496	0.2426	-2.8048	0.3546	0.3390	-4.4089	0.2678	0.2554	-4.6071	0.1852
0.2565	0.2318	-9.6166	0.5479	0.4825	-11.9327	0.3769	0.3391	-10.0305	0.2547
0.2133	0.2109	-1.1386	0.2948	0.2731	-7.3897	0.2267	0.2138	-5.6771	0.1667
0.2356	0.2253	-4.3814	0.4600	0.4045	-12.0753	0.3140	0.2887	-8.0750	0.2265
0.2613	0.2524	-3.3971	0.4336	0.4311	-0.5898	0.3286	0.3128	-4.8133	0.2002
0.2604	0.2391	-8.1733	0.5298	0.4604	-13.0909	0.3647	0.3179	-12.8330	0.2525
0.2694	0.2663	-1.1797	0.4194	0.4097	-2.3147	0.2929	0.2950	0.7073	0.2178
0.2063	0.1957	-5.1082	0.4407	0.4158	-5.6442	0.2954	0.2811	-4.8322	0.2235
0.2190	0.2169	-0.9785	0.4048	0.3787	-6.4541	0.2803	0.2705	-3.5099	0.2147
0.2466	0.2441	-1.0064	0.4015	0.4358	8.5399	0.2876	0.3151	9.5531	0.1995
0.2288	0.2210	-3.3880	0.3154	0.3279	3.9756	0.2398	0.2374	-1.0181	0.1570
0.2521	0.2495	-1.0412	0.2033	0.2079	2.2742	0.1769	0.1767	-0.0784	0.1245
0.2023	0.1954	-3.3993	0.3458	0.3185	-7.8900	0.2556	0.2417	-5.4350	0.1788
0.2403	0.2094	-12.8289	0.4773	0.4527	-5.1516	0.3397	0.3253	-4.2331	0.2257
0.2127	0.2055	-3.3915	0.3483	0.3338	-4.1700	0.2715	0.2610	-3.8513	0.1687
0.2431	0.2412	-0.8055	0.5131	0.4896	-4.5871	0.3281	0.3172	-3.3271	0.2268
0.2629	0.2443	-7.0715	0.2949	0.2429	-17.6204	0.2432	0.2081	-14.4289	0.1579
0.2454	0.2320	-5.4508	0.3389	0.3130	-7.6151	0.2571	0.2535	-1.3886	0.1754
0.2246	0.2134	-4.9678	0.3130	0.2891	-7.6214	0.2440	0.2269	-6.9951	0.1717
0.2600	0.2583	-0.6731	0.3016	0.3503	16.1495	0.2437	0.2650	8.7410	0.1690
0.2287	0.2092	-8.5322	0.3796	0.3163	-16.6635	0.2822	0.2457	-12.9306	0.1994
0.2333	0.2228	-4.5016	0.4438	0.4279	-3.5729	0.3050	0.2877	-5.6619	0.2164
0.2123	0.2090	-1.5312	0.3435	0.3703	7.8015	0.2613	0.2674	2.3226	0.1808
0.2153	0.2096	-2.6543	0.4019	0.4053	0.8629	0.2817	0.2769	-1.6748	0.2122
0.2510	0.2523	0.4980	0.3589	0.3957	10.2398	0.2655	0.2845	7.1515	0.1929
0.2324	0.2345	0.8912	0.4240	0.4701	10.8699	0.3018	0.3204	6.1479	0.2081
0.2489	0.2266	-8.9402	0.5762	0.5257	-8.7564	0.3710	0.3431	-7.5361	0.2764
0.2389	0.2395	0.2616	0.3289	0.3433	4.3602	0.2540	0.2630	3.5161	0.1735
0.2564	0.2526	-1.4627	0.4128	0.4505	9.1284	0.2846	0.2995	5.2292	0.2297
0.2231	0.2106	-5.6022	0.3790	0.3496	-7.7625	0.2757	0.2557	-7.2549	0.1937
0.2203	0.2194	-0.4349	0.3389	0.3350	-1.1581	0.2555	0.2481	-2.9076	0.1751
0.2469	0.2405	-2.5823	0.4389	0.4222	-3.8085	0.3022	0.2916	-3.5161	0.2073
0.2320	0.2205	-4.9569	0.3259	0.2860	-12.2545	0.2436	0.2194	-9.9342	0.1735
0.2178	0.2238	2.7555	0.2854	0.3063	7.3413	0.2283	0.2384	4.4363	0.1626
0.2299	0.2456	6.8282	0.3913	0.4182	6.8837	0.2836	0.3065	8.0580	0.1915
0.2561	0.2410	-5.9053	0.4290	0.4185	-2.4600	0.3241	0.3061	-5.5420	0.2230
0.1869	0.1830	-2.0642	0.3000	0.2783	-7.2434	0.2277	0.2132	-6.3845	0.1481
0.2439	0.2378	-2.5115	0.2774	0.2765	-0.3027	0.2373	0.2335	-1.5800	0.1470
0.2316	0.2318	0.0771	0.2680	0.3226	20.3455	0.2193	0.2501	14.0364	0.1497
0.2267	0.2146	-5.3630	0.3095	0.2935	-5.1812	0.2436	0.2318	-4.8433	0.1651
0.2351	0.2250	-4.3135	0.5188	0.5174	-0.2874	0.3649	0.3558	-2.5072	0.2461
0.2365	0.2201	-6.9239	0.5098	0.4776	-6.3146	0.3230	0.3133	-3.0038	0.2455
0.2147	0.2124	-1.0895	0.3375	0.3404	0.8444	0.2560	0.2521	-1.5008	0.1909
0.2301	0.2201	-4.3455	0.3702	0.3546	-4.2236	0.2704	0.2651	-1.9318	0.1827
0.2529	0.2595	2.6199	0.5417	0.6453	19.1357	0.3511	0.4049	15.3201	0.2553

Imin LowCa	*Imin* RCR
0.2660	0.7170
0.1819	1.7370
0.1927	2.7094
0.1684	-8.7750
0.1947	-4.9466
0.1436	4.1120
0.1824	-1.5289
0.2302	-9.6341
0.1550	-7.0268
0.2020	-10.8194
0.2003	0.0394
0.2298	-9.0086
0.2130	-2.1951
0.2120	-5.1549
0.1961	-8.6564
0.2117	6.1489
0.1600	1.9277
0.1261	1.2589
0.1675	-6.3360
0.2069	-8.2959
0.1631	-3.3068
0.2298	1.3246
0.1401	-11.3253
0.1652	-5.8168
0.1652	-3.7582
0.1842	9.0086
0.1689	-15.3103
0.2074	-4.1278
0.1907	5.4584
0.2095	-1.2311
0.2050	6.2376
0.2290	10.0139
0.2551	-7.6969
0.1800	3.7299
0.2411	4.9559
0.1846	-4.6955
0.1813	3.5398
0.2082	0.4656
0.1550	-10.6543
0.1688	3.8440
0.1906	-0.4717
0.2089	-6.3308
0.1588	7.2071
0.1472	0.1576
0.1710	14.1795
0.1602	-2.9793
0.2443	-0.7366
0.2451	-0.1624
0.1903	-0.2759
0.1806	-1.1049
0.2847	11.5172

Supplemental Table S3. Transformation and Covariate Correction for Each Femoral Bone Trait

Phenotypes	Transformation	Correction for Body Size
F_BMD	Log(y)	BW, FL
F_BMD_RCR	N/A	BW, FL
F_BMC	Log(y)	BW, FL
F_BMC_RCR	N/A	BW, FL
F_BV/TV	SQRT(y)	BW, FL
F_BV/TV_RCR	CURT(y+90)	FL
F_Tb.N	N/A	BW
F_Tb.N_RCR	N/A	FL
F_Tb.Th	Log(y)	FL
F_Tb.Th_RCR	SQRT(y+45)	FL
F_Tb.Sp	Log(y)	BW
F_Tb.Sp_RCR	N/A	FL
F_Conn.D	SQRT(y)	N/A
F_Conn.D_RCR	N/A	FL
F_SMI	N/A	BW, FL
F_SMI_RCR	SQRT(y+100)	FL
F_Tb.TMD	N/A	N/A
F_Tb.TMD_RCR	N/A	N/A
F_Ct.Ar	N/A	BW, FL
F_Ct.Ar_RCR	N/A	BW, FL
F_Tt.Ar	N/A	BW, FL
F_Tt.Ar_RCR	N/A	BW, FL
F_Ct.Ar/Tt.Ar	N/A	BW, FL
F_Ct.Ar/Tt.Ar_RCR	N/A	BW, FL
F_Ct.Th	N/A	BW, FL
F_Ct.Th_RCR	N/A	BW, FL
F_J	Log(y)	BW, FL
F_J_RCR	SQRT(y+35)	BW, FL
F_I_{max}	Log(y)	BW, FL
F_I_{max}_RCR	SQRT(y+30)	BW, FL
F_I_{min}	Log(y)	BW, FL
F_I_{min}_RCR	N/A	BW, FL

Abbreviation: F_ = Femur; BMD = bone mineral density (g/cm^2); BMC = bone mineral content (g); BV/TV = bone volume fraction; Tb.N = trabecular number (mm-1); Tb.Th = trabecular thickness (mm); Tb.Sp = trabecular separation (mm); Conn.D = connectivity density (1/mm3); SMI = structure model index; Tb.TMD = Trabecular tissue mineral density (mg of hydroxyappatite/cm^3); Ct.Ar = cortical bone area (mm^2) Tt.Ar = total cross-sectional area inside the periosteal envelope (mm^2); Ct.At/Tt.Ar = cortical area fraction Ct.Th = average cortical thickness (mm); J = polar moment of inertia (mm^4); I_{max} = maximum moment of inertia (mm^4); I_{min} = minimum moment of inertia (mm^4)
RCR = the response to Ca restriction; SQRT = square root transformation; Log = log10 transformation; CURT = cube root transformation; For (y+XX), XX is the number added to all the data points to make data non-negative and appropriate for data transformation
BW = body weight; FL = femur length; _RES = Body size-corrected residuals; N/A = not applicable

Supplemental Table S4. Body Size-Corrected and Transformed Line Means for Femoral Trabecular and Cortical Bone Traits and The

BXD line	ResBMD Basal	ResBMD LowCa	ResBMD RCR *	ResBMC Basal	ResBMC LowCa	ResBMC RCR *	ResBV/TV Basal	ResBV/TV LowCa
1	0.0172	0.0160	3.8003	0.0284	0.0663	8.8092	-0.0993	-0.1195
2	-0.0031	-0.0323	0.6186	-0.0409	-0.0413	4.2562	0.0563	0.0134
5	-0.0286	-0.0325	4.5425	-0.0462	-0.0061	11.7674	0.0671	0.0293
6	0.0762	0.0189	-1.4719	0.1184	0.0374	-2.4986	0.1205	0.0162
8	0.1030	0.0635	2.1354	0.1600	0.1084	4.5825	0.1255	0.0655
9	-0.0650	-0.0693	3.0140	-0.1152	-0.1045	6.1308	-0.0250	-0.0613
11	0.1566	0.1496	4.7569	0.2091	0.1747	5.0661	0.1654	0.1364
12	0.1682	0.0685	-7.0398	0.1984	0.1305	-5.8249	0.1476	0.0510
13	-0.0791	-0.0799	0.9482	-0.1279	-0.1166	-0.4487	-0.1069	-0.1269
14	0.0515	-0.0016	-4.4874	0.0499	0.0096	-6.5134	0.0647	0.0067
15	0.0823	0.0487	-1.5118	0.1227	0.0660	-5.3174	0.0025	-0.0394
16	0.0988	0.0438	-3.9803	0.1498	0.0575	-9.5188	0.0896	0.0232
18	0.0437	0.0152	-0.0669	0.0217	0.0220	2.0856	-0.0248	-0.0722
19	-0.0406	-0.1096	-4.2660	-0.0485	-0.0874	-2.9718	-0.0377	-0.1004
20	0.0400	0.0000	-2.2698	0.0811	0.0118	-8.1197	0.0577	0.0040
21	-0.0410	-0.0535	1.1587	-0.0599	-0.0963	-2.4005	-0.0744	-0.0160
22	0.0077	-0.0071	4.8372	-0.0344	-0.0256	12.4878	-0.0117	-0.0590
24	0.0026	-0.0208	2.3838	-0.0181	-0.0783	1.4741	-0.0952	-0.1403
27	-0.0131	-0.0822	-0.7966	0.0043	-0.0696	3.2285	-0.0275	-0.0524
28	0.0245	-0.0518	-5.5970	0.0602	0.0274	-3.4730	0.0503	-0.0363
29	0.0019	-0.0700	-2.8854	-0.0380	-0.1030	-0.5645	0.0288	-0.0153
31	0.0556	0.0481	2.6760	0.1273	0.1084	2.5264	-0.0086	0.0436
32	0.1078	0.0399	-0.6897	0.0761	0.0125	3.7797	0.0921	0.0093
33	-0.0233	-0.0776	-0.6966	-0.0083	-0.0618	1.2496	0.0319	-0.0070
34	0.0238	-0.0637	-4.0734	0.0292	-0.0870	-4.6301	-0.0449	-0.0680
36	0.0561	-0.0008	-3.0446	0.0318	0.0036	-1.4420	-0.0105	-0.0439
38	0.0392	-0.0744	-8.9784	0.0360	-0.1428	-16.5920	0.1199	0.0198
39	0.0751	0.0028	-3.5873	0.1136	0.0212	-4.7903	0.1420	0.0762
40	-0.0102	-0.0089	4.4238	0.0215	0.0137	5.1179	-0.0818	-0.0700
42	-0.0339	-0.0430	3.0670	-0.0032	-0.0227	3.6252	-0.0680	-0.0502
44	0.0347	0.0187	-1.4853	-0.0194	-0.0262	-4.9275	0.0300	0.0230
48	0.0271	0.0697	8.9884	0.0734	0.1262	12.1563	-0.0022	0.0171
49	0.0445	-0.0529	-9.8860	0.0797	-0.0542	-19.4455	0.1081	0.0050
50	0.0493	0.0290	0.9407	0.0045	-0.0040	1.3545	0.0065	0.0505
51	0.0627	0.0306	-0.1096	0.0505	0.0211	-0.2554	-0.0002	-0.0173
55	0.0088	-0.0414	-3.6885	0.0112	-0.0490	-8.4778	0.0408	-0.0476
56	-0.0475	-0.0742	0.2909	-0.0305	-0.0708	-1.0831	-0.0228	-0.0684
62	0.0535	0.0245	-0.0071	0.0645	0.0317	-1.8223	0.0260	-0.0061
68	-0.0251	-0.0735	-1.6789	-0.0060	-0.0923	-5.9461	0.0069	-0.0465
69	0.0143	0.0162	6.5335	0.0367	-0.0141	6.1288	0.1031	0.1157
73	0.0578	0.0669	3.8658	0.0507	0.0731	5.1843	0.0953	0.1363
75	0.0321	-0.0381	-3.7850	0.0632	-0.0883	-9.3491	-0.0719	-0.0989
84	-0.1489	-0.1846	1.2420	-0.1855	-0.2261	3.8750	-0.0702	-0.0904
86	-0.0267	-0.0468	-0.4798	-0.0691	-0.1251	-5.9909	0.0640	0.0226
87	0.0376	0.0636	11.7064	0.0192	0.0480	20.8605	0.0302	0.0699
89	-0.0766	-0.1147	0.7957	-0.0772	-0.1609	-1.4242	-0.0807	-0.1262
90	0.0013	-0.0287	-1.1906	0.0047	-0.0690	-7.1995	-0.0059	-0.0289
96	0.0488	-0.0312	-5.6985	0.0358	-0.0235	-5.7204	0.0432	-0.0703
97	-0.1013	-0.1255	-1.2080	-0.1673	-0.1423	1.4642	-0.0921	-0.1179
98	-0.0237	-0.0355	2.1598	-0.0385	-0.0143	4.8482	-0.0070	-0.0379
100	0.0468	0.0514	3.2840	0.0771	0.1237	6.5268	0.0738	0.0488

Abbreviation: BMD = bone mineral density (g/cm^2); BMC = bone mineral content (g); BV/TV = bone volume fraction; Tb.N = trabecular number (mm-1); Tb.Th
SMI = structure model index; Tb.TMD = Trabecular tissue mineral density (mg of hydroxyappatite/cm^3); Ct.Ar = cortical bone area (mm^2); Tt.Ar = total cross-sect
J = polar moment of inertia (mm^4); *Imax* = maximum moment of inertia (mm^4); *Imin* = minimum moment of inertia (mm^4); RCR = the response to Ca restriction.
Res = Body size-corrected residuals; * = No body size correction required
Line means were calculated from Body size-corrected residuals obtained from linear regression of each phenotype with body weight and/or femur length (See Suppl

ir Responses to Ca Restriction (RCR) in 51 BXD RI Strains and Their Parental Strains

ResBV/TV RCR	ResTb.N Basal	ResTb.N LowCa	ResTb.N RCR	ResTb.Th Basal	ResTb.Th LowCa	ResTb.Th RCR	ResTb.Sp Basal	ResTb.Sp LowCa
-0.2561	-1.6237	-1.7193	1.7542	0.0424	0.0134	-0.3827	0.1860	0.2108
-0.0106	0.3673	-0.2235	-4.8803	0.0237	0.0115	0.1001	-0.0456	0.0146
0.1296	0.7263	0.0870	-3.6222	0.0126	-0.0045	0.1323	-0.0737	-0.0075
-0.3586	1.2879	0.3506	-9.5380	0.0464	-0.0015	-0.4923	-0.1394	-0.0377
-0.0146	1.6974	1.2683	0.9690	0.0203	-0.0189	-0.2912	-0.1730	-0.1212
-0.0117	-0.2252	-0.4666	1.8340	-0.0073	-0.0265	0.0969	0.0306	0.0484
0.2083	1.5592	1.5769	7.2582	0.0685	0.0483	0.0840	-0.1663	-0.1661
-0.3331	0.1391	-0.0642	2.3862	0.1671	0.0748	-1.7545	-0.0559	-0.0156
0.0292	-0.5373	-0.3656	8.4859	-0.0667	-0.0946	-0.1886	0.0619	0.0320
-0.1602	0.2808	0.0115	-0.4105	0.0643	0.0282	-0.4317	-0.0471	-0.0118
-0.1580	0.1457	-0.1852	-2.6429	0.0198	-0.0013	-0.1654	-0.0235	0.0105
-0.2233	0.4058	0.3416	3.5061	0.0379	-0.0120	-0.6401	-0.0595	-0.0426
-0.1293	0.1758	-0.2125	-1.1244	-0.0344	-0.0599	-0.1667	-0.0123	0.0292
-0.3875	-0.3453	-0.7603	-5.4744	-0.0285	-0.0663	-0.4793	0.0391	0.0776
-0.1259	0.2411	-0.2881	-4.9403	0.0552	0.0670	0.4513	-0.0308	0.0227
0.6179	-0.3353	-0.2386	6.6895	-0.0417	0.0191	1.2539	0.0284	0.0188
-0.0083	-0.2380	-0.2850	6.8370	0.0139	-0.0143	0.0167	0.0214	0.0255
-0.0903	-0.9808	-1.3325	-1.3398	-0.0461	-0.0288	0.7503	0.1064	0.1526
0.1544	-0.3173	-0.2681	8.6899	0.0038	-0.0453	-0.4139	0.0386	0.0107
-0.2920	1.1398	0.2809	-7.6124	-0.0116	-0.0454	-0.2449	-0.1042	-0.0345
-0.0196	0.3208	0.1872	3.8117	0.0100	-0.0267	-0.2855	-0.0335	-0.0172
0.6782	-0.5579	-0.8323	-0.1269	0.0427	0.1307	1.8788	0.0545	0.0763
-0.1376	0.7123	0.0954	-3.8222	0.0502	0.0155	-0.0919	-0.0959	-0.0120
0.0861	1.1601	0.6945	-0.7695	-0.0533	-0.0644	0.2497	-0.1064	-0.0654
0.0218	-0.9443	-1.1677	0.2153	0.0146	-0.0236	-0.3487	0.1040	0.1358
-0.0373	0.2809	-0.3582	-7.2064	-0.0269	-0.0342	0.1194	-0.0221	0.0354
-0.4021	1.8726	1.2467	-4.3006	-0.0067	-0.0817	-1.1247	-0.1719	-0.1067
-0.0246	1.4477	0.8739	-1.3184	0.0494	0.0174	-0.1793	-0.1308	-0.0813
0.3254	-0.1663	-0.3103	2.3117	-0.0798	-0.0546	0.7225	0.0182	0.0133
0.3396	-1.2865	-1.2996	4.2804	0.0068	-0.0178	-0.2246	0.1531	0.1632
0.1262	0.3935	0.1607	-0.1786	0.0027	-0.0138	-0.0808	-0.0423	-0.0208
0.3818	0.1731	-0.3047	-3.0440	-0.0281	0.0181	1.1028	-0.0230	0.0218
-0.5341	0.7977	0.1393	-7.6692	0.0641	0.0029	-1.1119	-0.0935	-0.0158
0.5598	0.6391	0.4942	3.0383	-0.0310	0.0237	1.2000	-0.0616	-0.0454
0.0851	0.3571	0.4039	6.1624	-0.0347	-0.0351	0.2377	-0.0444	-0.0409
-0.4632	0.1026	-0.2621	-2.9370	0.0293	-0.0126	-0.6190	-0.0206	0.0203
-0.1870	-0.0840	-0.1560	2.5718	-0.0344	-0.0690	-0.3642	0.0065	0.0155
0.0081	-0.0474	-0.3833	-1.0271	0.0304	0.0170	0.0210	-0.0054	0.0362
-0.1357	-0.7043	-1.1612	-4.7269	0.0548	0.0357	-0.1003	0.0621	0.1291
0.5562	1.0456	1.1742	11.1216	0.0252	0.0268	0.5847	-0.1255	-0.1148
0.4308	0.8539	1.2163	11.1328	0.0621	0.0728	0.3535	-0.0996	-0.1365
-0.0713	-1.3731	-1.3426	4.7100	0.0397	-0.0038	-0.5199	0.1587	0.1475
0.1037	-0.5810	-0.8610	-0.7814	-0.0526	-0.0650	0.1727	0.0608	0.0958
-0.0133	0.0843	-0.1208	1.4710	0.0559	0.0363	-0.0459	-0.0187	0.0056
0.8157	0.2021	-0.0179	5.2056	0.0214	0.0617	1.3722	-0.0241	-0.0055
-0.2242	-0.5458	-1.1626	-8.7952	-0.0386	-0.0387	0.2672	0.0635	0.1359
-0.0640	0.6518	0.4650	-0.0411	-0.0487	-0.0550	0.0081	-0.0624	-0.0433
-0.6178	-0.0650	-0.7160	-8.5908	0.0256	-0.0563	-1.2834	-0.0064	0.0763
-0.1030	-0.5845	-0.8713	-3.2351	-0.0688	-0.0715	0.1475	0.0740	0.1013
0.1338	-0.1164	-0.5891	-2.8200	-0.0137	0.0021	0.6650	0.0104	0.0610
-0.0787	0.7852	0.3567	-2.9680	0.0156	0.0106	0.0077	-0.0797	-0.0403

= trabecular thickness (mm); Tb.Sp = trabecular separation (mm); Conn.D = connectivity density (1/mm3);

ional area inside the periosteal envelope (mm^2); Ct.Ar/Tt.Ar = cortical area fraction;

l.Table S3.2)

ResTb.Sp RCR	ResConn.D Basal *	ResConn.D LowCa *	ResConn.D RCR	ResSMI Basal	ResSMI LowCa	ResSMI RCR	ResTb.TMD Basal *
0.3125	6.0162	5.4462	-0.6922	2.0513	2.8443	-4.2581	6.6912
5.0068	11.3406	10.0858	-0.1629	-1.6916	-1.1945	-3.4189	6.6351
5.4077	12.4509	10.8820	-0.1210	-1.9406	-1.4488	-12.1726	6.6326
18.3647	13.7142	10.9919	-1.0953	-2.7765	0.0383	32.2949	6.6292
2.2108	14.3318	13.1028	0.3332	-2.6922	-1.2305	7.9676	6.5783
-7.5841	9.3697	8.8864	0.1972	0.1495	1.2776	-2.4341	6.6163
-9.8682	14.1781	14.1113	1.1100	-4.0347	-3.4689	2.4644	6.6315
1.6445	9.0335	8.4804	0.4698	-3.3690	-1.0660	55.3787	6.7639
-14.8875	7.4857	7.0108	-0.1490	3.4026	4.2586	-5.6523	6.6563
1.8031	10.1438	9.8300	0.5210	-1.9251	-0.4413	18.1542	6.6824
3.6492	9.8209	9.2213	-0.0207	1.1677	1.6113	-2.0811	6.6650
-1.4084	11.0354	10.7100	0.5097	-2.8197	-0.7499	26.8962	6.6705
1.0031	9.9649	8.4069	-1.0338	1.4666	2.8847	0.0339	6.6231
3.8236	9.0873	7.3624	-1.4070	0.8927	2.4386	7.5424	6.6105
4.9203	10.9750	8.9868	-0.8915	-1.8853	-0.7352	7.5208	6.6337
-8.1385	8.3952	8.8590	0.8336	1.9382	0.9366	-12.7117	6.6768
-9.8010	8.9279	8.0729	0.1322	0.7748	0.9485	-19.6255	6.6756
1.0111	7.1585	5.7186	-1.1185	2.9426	3.7690	-11.0561	6.6858
-18.3026	9.0127	9.2013	1.1641	0.6671	1.1900	-12.7694	6.6471
8.0701	13.1715	10.1934	-1.2556	-0.8068	1.6260	14.2224	6.5801
-4.7407	10.4235	9.4774	0.1217	-0.2402	0.6534	-6.3958	6.6186
-3.8223	8.2366	7.9709	0.5351	-0.4075	-0.7598	-18.9024	6.6871
11.2971	11.5318	9.7043	-0.3254	-2.1887	-0.1869	7.8690	6.6545
0.0034	13.1661	11.7301	0.0551	-0.5066	0.4342	-6.7685	6.5849
-0.7744	7.7946	7.7191	0.6779	0.8675	0.7259	-11.9838	6.6835
6.5635	10.7560	9.4855	-0.5646	1.2357	1.1557	-6.6194	6.6198
9.4735	15.0938	13.6202	-0.2081	-3.0739	-0.6413	47.6359	6.5564
1.9764	14.0577	13.0422	0.4143	-3.6406	-2.3658	23.2385	6.5803
-8.5057	8.0191	7.9877	0.4900	2.8892	2.5776	-11.9480	6.6046
-3.0058	7.7718	9.0268	2.3504	0.5421	-0.3423	-14.2632	6.6505
-0.4892	11.2791	10.6595	0.0465	-0.5897	-0.8165	-8.5012	6.6387
3.2290	10.4047	9.0326	-0.6215	-0.4462	-0.8732	-21.9788	6.6174
15.3041	11.7055	9.9868	-0.9206	-2.6132	-0.1868	52.8327	6.6468
-3.6315	11.2343	11.1655	0.7333	1.0415	-0.7678	-25.1455	6.6377
-6.3015	10.6405	10.1888	0.2697	0.4242	0.8572	-3.7042	6.6488
3.9156	10.5477	8.8104	-1.0785	-1.0122	0.9332	16.4812	6.6889
-3.5481	9.3386	9.2715	0.5983	0.8181	2.4770	7.7166	6.6547
2.2432	9.9325	9.1635	0.0585	-0.8477	-0.1375	-2.8793	6.6647
7.6921	8.7455	8.0226	0.1414	-0.8853	0.1827	0.9877	6.6839
-11.0968	13.1168	13.6911	1.8617	-2.9976	-3.5125	-29.4005	6.6010
-15.2334	11.9720	12.7858	1.4257	-3.0398	-3.0908	-20.6437	6.6367
-9.8926	6.9996	5.9135	-1.1020	0.7877	2.0414	1.1413	6.7046
0.2513	8.3534	8.0138	0.4423	1.6055	1.7970	-10.3793	6.6140
-1.5032	11.0681	10.4408	0.3631	-1.8223	-0.6801	4.4475	6.6579
-9.1623	9.9681	10.2606	1.8115	-0.7980	-3.0084	-47.5015	6.6671
10.0644	7.7090	6.1078	-1.4230	2.5598	2.8073	-10.8921	6.6511
0.0333	11.2099	10.3826	-0.3599	0.9830	1.8732	5.8362	6.5893
13.8650	10.2617	8.1542	-1.3947	-1.9621	0.2599	24.4583	6.6419
1.6569	8.8949	7.9852	-0.5584	2.8871	3.5208	0.5421	6.6068
2.4956	9.7031	8.5404	0.0351	0.0465	0.6960	-10.3243	6.6258
2.7041	12.6808	11.5753	-0.5657	-1.4692	-1.4285	0.4786	6.5980

ResTb.TMD LowCa *	ResTb.TMD RCR *	ResCt.Ar Basal	ResCt.Ar LowCa	ResCt.Ar RCR	ResTt.Ar Basal	ResTt.Ar LowCa	ResTt.Ar RCR	ResCt.Ar/Tt.Ar Basal
6.6927	0.1977	0.1242	0.1084	1.4630	0.1927	0.1906	0.0724	-0.0308
6.6445	0.9312	-0.0426	-0.0330	3.8955	-0.0199	0.0162	0.3177	-0.0080
6.6252	-0.7912	-0.0566	-0.0619	3.2593	0.0527	0.0676	0.2408	-0.0601
6.6324	0.3287	0.0705	0.0349	-0.6332	-0.0444	-0.0713	-0.1760	0.0700
6.5570	-2.1088	0.1093	0.0667	2.0495	0.1005	0.1007	0.3931	0.0105
6.6095	-0.6642	-0.1440	-0.1728	0.6726	-0.1725	-0.1749	0.1982	0.0003
6.5999	-3.1368	0.0656	0.0371	2.0492	0.0015	-0.0162	0.0842	0.0387
6.6765	-8.3805	0.2157	0.1334	-6.2384	0.1921	0.1994	-0.1653	0.0115
6.6502	-0.5160	-0.1468	-0.1594	-3.1645	-0.1390	-0.1575	-0.4067	-0.0146
6.6629	-1.9412	0.0035	-0.0364	-6.3928	0.0635	0.0122	-0.8690	-0.0183
6.6716	0.6740	0.0777	0.0347	-4.0563	-0.0324	-0.0495	-0.2369	0.0641
6.6204	-4.9157	0.1475	0.0465	-9.6516	0.1263	0.0814	-0.6071	0.0103
6.6226	-0.2083	0.1024	0.0705	-1.2298	0.0144	-0.0158	-0.3259	0.0625
6.5955	-1.4975	-0.0431	-0.0891	-4.3711	0.0749	0.0624	-0.1920	-0.0608
6.7033	7.2761	-0.0170	-0.0116	-1.2778	0.0332	0.0614	-0.1896	-0.0271
6.6783	0.1593	-0.0081	0.0249	4.8733	-0.0296	-0.0032	0.1858	0.0245
6.6580	-1.6768	0.0325	0.0229	5.0935	-0.0001	0.0156	0.5996	0.0177
6.6829	-0.3548	-0.0984	-0.1009	3.8119	-0.3065	-0.2974	0.2330	0.1133
6.6278	-1.9684	-0.0393	-0.0870	-0.6535	0.0658	0.0322	0.0419	-0.0571
6.6158	3.6786	0.0962	0.0289	-6.5999	0.1490	0.1454	-0.2314	-0.0067
6.6244	0.6984	-0.0420	-0.0416	2.9960	-0.0193	0.0000	0.2675	-0.0067
6.7249	3.7037	0.1554	0.1238	-0.6142	0.1389	0.1323	-0.0130	0.0094
6.6636	0.8385	0.0616	-0.0057	-2.1297	-0.0779	-0.1392	-0.3313	0.0868
6.6439	6.2440	0.0459	0.0231	0.8174	-0.0271	-0.0198	0.1903	0.0443
6.6515	-3.1529	-0.0614	-0.1032	-2.0152	-0.0762	-0.0914	-0.0800	-0.0012
6.6452	2.5911	-0.0424	-0.0145	4.2890	-0.1774	-0.1205	0.4635	0.0771
6.5799	2.5401	-0.0458	-0.1147	-8.6681	-0.0279	-0.0652	-0.7031	-0.0114
6.6016	2.1308	0.0703	0.0110	-3.7912	0.1284	0.0947	-0.3198	-0.0274
6.6371	3.3035	-0.0634	-0.0487	4.9049	-0.0158	0.0252	0.4606	-0.0376
6.6271	-2.3624	-0.0239	-0.0242	3.7948	0.0638	0.0751	0.2016	-0.0443
6.6653	2.7430	-0.0358	-0.0363	-2.4804	-0.1166	-0.0980	-0.1347	0.0335
6.6489	3.1866	0.0477	0.0781	6.5344	0.0597	0.0980	0.4393	-0.0045
6.6383	-0.9475	0.1073	0.0492	-9.1031	0.1219	0.1402	-0.4929	-0.0126
6.6353	-0.2983	-0.0399	-0.0129	4.1856	-0.1079	-0.0620	0.2937	0.0352
6.6605	1.2242	0.0743	0.0859	3.3881	0.0248	0.0568	0.3264	0.0290
6.6520	-3.6359	-0.0842	-0.1054	-4.3831	-0.0688	-0.0496	-0.2500	-0.0126
6.6209	-3.3677	-0.0990	-0.1045	1.2875	-0.0856	-0.0974	-0.0495	-0.0122
6.6704	0.6059	0.0664	0.0595	0.4729	0.0337	0.0330	-0.1530	0.0210
6.6647	-1.8603	-0.0366	-0.0610	-3.0682	-0.0971	-0.1006	-0.3293	0.0249
6.6011	-0.0402	-0.0055	0.0037	10.0884	0.0060	0.0125	0.5799	-0.0130
6.6404	0.3503	-0.0213	0.0369	7.7681	-0.0114	-0.0272	-0.4070	0.0009
6.7086	0.3084	0.1269	0.0476	-4.9086	0.0375	0.0031	-0.2677	0.0615
6.6386	2.5561	-0.1354	-0.1835	-0.7133	-0.0353	-0.0645	0.0608	-0.0663
6.6599	0.1644	-0.0814	-0.1080	-3.6575	-0.1995	-0.2051	-0.0736	0.0602
6.6862	1.9648	0.0315	0.0689	17.2421	-0.0243	0.0368	1.1250	0.0316
6.6532	0.3135	-0.0595	-0.1007	-1.3581	-0.0876	-0.0933	0.0933	0.0154
6.6298	4.1771	0.0828	0.0232	-5.5277	0.1161	0.0828	-0.4019	-0.0100
6.6257	-1.6471	0.0787	0.0394	-4.0024	0.1155	0.1437	0.0025	-0.0187
6.6338	2.7870	-0.1406	-0.1593	-3.1951	-0.0940	-0.0972	-0.2287	-0.0325
6.6518	2.6373	0.0104	0.0272	2.0436	-0.0021	0.0559	0.2682	0.0093
6.6276	2.9876	0.1298	0.1630	5.0610	0.0978	0.1258	0.3409	0.0110

ResCt.Ar/Tt.Ar LowCa	ResCt.Ar/Tt.Ar RCR	ResCt.Th Basal	ResCt.Th LowCa	ResCt.Th RCR	Res*J* Basal	Res*J* LowCa	Res*J* RCR
-0.0410	-2.0801	0.0075	0.0033	1.6921	0.3346	0.3163	0.0958
-0.0322	-4.5138	-0.0108	-0.0242	-2.8807	-0.0591	0.0015	0.6171
-0.0725	-2.0635	-0.0225	-0.0275	1.8601	0.0170	0.0270	0.3895
0.0621	-1.3348	0.0290	0.0195	-0.3473	0.0143	-0.0258	-0.1845
-0.0086	-3.1542	0.0188	0.0064	0.5248	0.2114	0.1986	0.8044
-0.0126	-2.7489	-0.0156	-0.0234	-0.3325	-0.3488	-0.3598	0.3103
0.0321	-1.0885	0.0236	0.0157	1.4420	0.0504	-0.0016	0.0293
-0.0300	-7.0402	0.0266	0.0054	-5.2556	0.3888	0.3540	-0.7004
-0.0151	-0.7462	-0.0229	-0.0230	1.0971	-0.2995	-0.3329	-0.7774
-0.0319	-3.1202	-0.0024	-0.0111	-2.8638	0.0969	-0.0114	-1.6741
0.0582	-1.2867	0.0253	0.0157	-2.0216	0.0434	0.0107	-0.3681
-0.0164	-4.9641	0.0238	0.0042	-6.2736	0.2641	0.1402	-1.3989
0.0508	-1.5364	0.0365	0.0297	0.3091	0.0857	0.0143	-0.5855
-0.0785	-3.8967	-0.0269	-0.0368	-2.5815	0.0638	0.0178	-0.4577
-0.0374	-1.3353	-0.0133	-0.0108	3.0159	0.0156	0.0796	-0.1639
0.0150	-1.8288	0.0120	0.0078	0.4570	-0.0213	0.0239	0.3230
0.0047	-2.3361	0.0063	-0.0005	2.4083	0.0445	0.0768	1.2905
0.0978	-2.1069	0.0249	0.0216	2.9265	-0.5061	-0.4962	0.4730
-0.0625	-1.1090	-0.0229	-0.0310	0.9986	0.0707	-0.0136	-0.0162
-0.0564	-9.0063	0.0062	-0.0237	-10.2944	0.2642	0.2311	-0.4777
-0.0249	-3.0706	-0.0136	-0.0190	1.4745	-0.0150	-0.0043	0.4102
-0.0011	-2.1678	0.0117	0.0093	2.0105	0.3251	0.2810	-0.3541
0.0737	-2.2024	0.0396	0.0229	-1.2563	-0.0676	-0.2055	-0.6422
0.0270	-2.8991	0.0194	0.0069	-0.7274	-0.0037	-0.0179	0.1429
-0.0163	-2.5837	-0.0037	-0.0144	-1.0474	-0.1521	-0.2192	-0.4047
0.0605	-2.4369	0.0265	0.0226	0.9933	-0.2883	-0.1721	1.0232
-0.0337	-3.7337	-0.0041	-0.0208	-5.2688	-0.0825	-0.1815	-1.4536
-0.0440	-2.7252	0.0017	-0.0115	-2.2907	0.2244	0.1410	-0.7413
-0.0394	-0.1762	-0.0155	-0.0181	2.5405	-0.0744	0.0001	0.9629
-0.0607	-3.5678	-0.0135	-0.0195	0.8409	0.0612	0.0648	0.2476
0.0340	0.0022	0.0108	0.0112	0.6635	-0.1937	-0.1192	0.1527
-0.0082	-0.2009	0.0029	0.0020	3.4686	0.1247	0.1878	0.8320
-0.0472	-6.0246	0.0098	-0.0093	-7.3618	0.2184	0.2132	-1.3021
0.0274	-0.8033	0.0084	0.0096	3.5559	-0.1774	-0.0953	0.6151
0.0197	-1.4570	0.0248	0.0198	0.9964	0.0485	0.1073	0.6716
-0.0351	-3.5239	-0.0109	-0.0210	-2.8464	-0.1417	-0.1418	-0.6402
-0.0085	0.5216	-0.0129	-0.0142	1.7780	-0.1725	-0.2088	-0.1249
0.0054	-2.4304	0.0146	0.0075	-0.1098	0.1269	0.1071	-0.3002
0.0113	-1.6108	0.0021	-0.0060	-0.6402	-0.1569	-0.1872	-0.6145
-0.0057	1.7997	-0.0050	0.0006	8.1139	-0.0158	0.0031	1.3197
0.0348	6.3401	-0.0022	0.0134	9.4652	-0.0196	0.0445	0.4061
0.0362	-4.8097	0.0257	0.0119	-2.3923	0.1169	0.0582	-0.1465
-0.0812	-3.5381	-0.0396	-0.0445	1.9357	-0.1490	-0.2546	-0.2571
0.0494	-1.8362	0.0083	0.0028	-0.5757	-0.3104	-0.3372	-0.1993
0.0278	-0.4009	0.0143	0.0135	6.7912	-0.0119	0.1090	2.8346
-0.0112	-4.6839	-0.0016	-0.0131	-1.4066	-0.1593	-0.2005	-0.0213
-0.0317	-4.3205	-0.0012	-0.0128	-3.3265	0.1726	0.1325	-0.4196
-0.0518	-5.9613	0.0027	-0.0132	-4.6046	0.2036	0.1864	-0.5382
-0.0417	-2.3382	-0.0237	-0.0264	-0.3377	-0.2554	-0.2734	-0.5455
-0.0126	-2.9726	0.0025	-0.0029	1.0071	0.0376	0.1251	0.5182
0.0086	-0.1316	0.0183	0.0163	2.0899	0.2206	0.2833	0.6381

Res*Imax* Basal	Res*Imax* LowCa	Res*Imax* RCR	Res*Imin* Basal	Res*Imin* LowCa	Res*Imin* RCR
0.2238	0.1917	0.0226	0.2994	0.2882	1.5428
-0.0574	-0.0198	0.4862	-0.0515	-0.0283	3.3591
-0.0414	-0.0455	0.2325	0.0560	0.0654	5.2233
0.0143	-0.0343	-0.1715	-0.0121	-0.0881	-5.1914
0.1630	0.1295	0.5294	0.1787	0.1454	2.8167
-0.2396	-0.2589	0.1037	-0.2691	-0.3009	1.6555
0.0542	-0.0009	-0.0638	0.0544	0.0279	2.5444
0.3271	0.2855	-0.6346	0.2863	0.2624	-5.3118
-0.2363	-0.2626	-0.5995	-0.1793	-0.2206	-7.3087
0.0530	-0.0134	-1.1642	0.0815	-0.0142	-13.5260
0.1078	0.0389	-0.6178	-0.0405	-0.0721	-3.8125
0.2260	0.0994	-1.4240	0.2104	0.1271	-10.7411
0.0575	0.0154	-0.2019	0.1030	0.0467	-4.9249
0.0091	-0.0313	-0.3024	0.0686	0.0225	-5.7029
-0.0153	0.0351	0.0399	0.0612	0.0788	-5.3409
-0.0044	0.0488	0.6063	-0.0327	-0.0035	3.0972
0.0083	0.0073	0.7227	-0.0271	-0.0137	9.8868
-0.3634	-0.3711	0.3039	-0.3459	-0.3376	4.7362
0.0362	-0.0195	0.0854	0.0483	-0.0423	-1.8475
0.2238	0.1944	-0.4178	0.1861	0.1174	-8.2018
0.0379	0.0338	0.3424	-0.0891	-0.0786	2.1070
0.1908	0.1546	-0.2044	0.1895	0.1962	1.8816
0.0042	-0.1127	-0.6025	-0.0567	-0.1477	-3.3606
0.0083	0.0194	0.4874	-0.0120	-0.0216	0.5005
-0.0920	-0.1544	-0.2948	-0.0935	-0.1208	-0.6815
-0.1668	-0.1061	0.6778	-0.1887	-0.1255	6.4717
-0.0326	-0.1140	-1.0737	-0.0432	-0.1426	-13.9443
0.1399	0.0497	-0.8732	0.1687	0.0929	-4.8154
-0.0361	-0.0063	0.6334	-0.0583	0.0005	8.7492
0.0270	0.0106	0.1726	0.0854	0.0728	1.3494
-0.1431	-0.0907	0.1402	-0.1136	-0.0707	0.1212
0.1019	0.1302	0.6058	0.0800	0.1438	9.8058
0.1559	0.1373	-1.0214	0.1912	0.1811	-10.4838
-0.1056	-0.0433	0.6236	-0.1428	-0.0764	5.1854
0.0072	0.0345	0.4982	0.1381	0.1628	3.5552
-0.0961	-0.1142	-0.5096	-0.1230	-0.1055	-4.7210
-0.1180	-0.1645	-0.1726	-0.1548	-0.1431	2.1092
0.0768	0.0532	-0.2331	0.0505	0.0513	0.0443
-0.1265	-0.1611	-0.4755	-0.1226	-0.1511	-6.5496
-0.0007	0.0066	1.0288	0.0410	0.0398	10.8676
-0.0051	0.0694	0.7213	-0.0576	-0.0671	-0.6548
0.1359	0.0785	-0.3789	0.1106	0.0368	-5.8227
-0.1298	-0.2164	-0.1992	-0.1976	-0.1615	10.4540
-0.1573	-0.1887	-0.2611	-0.2700	-0.2888	-1.4290
0.0113	0.0957	2.2760	0.0073	0.0885	24.3282
-0.0920	-0.1326	-0.0168	-0.1271	-0.1478	0.3294
0.1799	0.1303	-0.4187	0.1190	0.0817	-5.1825
0.1006	0.0950	-0.1714	0.1618	0.1845	-1.1098
-0.1753	-0.2091	-0.5457	-0.1227	-0.1493	-5.1008
0.0242	0.0952	0.5826	-0.0143	0.0853	5.6791
0.1502	0.2058	0.7218	0.1710	0.1818	3.9415

Supplemental Table S5. QTLs Influencing Femoral Bone Traits in Male BXD RI Mice

Chr	Trait	Diet	Loci ID	cM	mm10 Mb	Lower 1.5-LOD CI	Upper 1.5-LOD CI	LRS	LOD	Sig Threshold	Signif	Putative	Direction	r^2	r^2t	a0	S0	H/L of same Trait	Match to Previous QTL*	High Priority
1	TbTMD	H	1A	6.15	19.47	13.31	22.12	19.878	4.31	3.5	1	0	DBA	0.1654	0.653	-0.017	0.243			
1	CtTh	H	1B	28.43	55.86	43.09	59.61	12.372	2.69	3.8	0	1	DBA	0.1012	0.632	-0.006	0.458			
1	TbSp	RCR	1C	50.50	108.61	101.10	114.86	21.263	4.61	3.6	1	0	DBA	0.1463	0.718	-3.197	0.636			
1	TbTMD	RCR	1C	56.00	128.30	107.47	140.44	10.581	2.30	3.5	0	1	DBA	0.0874	0.623	-0.855	13.500			
1	TbSp	RCR	1D	64.14	151.46	136.26	158.81	12.688	2.75	3.6	0	1	B6	0.0820	0.718	2.467	1.229			
1	TbTh	RCR	1D	67.86	156.86	140.44	168.58	16.201	3.51	3.5	1	0	DBA	0.1425	0.619	-0.265	2.951			
1	BVTV	RCR	1D	72.99	165.65	149.88	168.58	10.972	2.38	3.7	0	1	DBA	0.0713	0.703	-0.083	0.275			
1	BMC	L	1D	77.99	170.96	160.12	170.99	10.086	2.19	3.6	0	1	DBA	0.0445	0.797	-0.020	0.146			
1	BMC	L	1E	90.31	187.09	184.25	187.21	23.893	5.19	3.6	1	0	B6	0.1356	0.774	0.033	1.317			
1	MOI	H	1F	91.51	187.51	176.63	195.27	10.786	2.34	3.7	0	1	B6	0.0738	0.687	0.053	2.130		25OHD H/L	
1	Imax	H	1F	92.82	188.40	183.88	195.27	10.112	2.20	3.7	0	1	B6	0.0731	0.667	0.039	0.844			
2	TbTMD	H	2A	0.87	1.56	0.02	4.99	27.302	5.92	3.5	1	0	DBA	0.2385	0.663	-0.021	0.131			
2	BMD	H	2A	2.46	4.25	1.56	4.99	34.856	7.58	3.5	1	0	B6	0.2988	0.709	0.033	0.908			
2	TbN	H	2B	20.21	29.03	26.56	30.24	20.717	4.49	3.6	1	0	B6	0.1408	0.719	0.308	1.497	1		1
2	ConnD	H	2B	20.96	29.78	26.56	29.90	32.335	7.01	3.8	1	0	B6	0.2186	0.755	1.057	0.963			
2	TbN	L	2B	21.00	29.83	29.83	31.01	20.824	4.52	3.7	1	0	B6	0.1597	0.683	0.299	4.939	1		
2	TbSp	H	2B	21.07	29.90	26.56	31.01	24.418	5.30	3.7	1	0	DBA	0.1822	0.704	-0.036	7.672			
2	ConnD	L	2C	25.48	43.20	33.04	46.53	28.999	6.29	3.8	1	0	B6	0.1929	0.748	0.949	0.194			1
2	BVTV	H	2C	25.48	43.20	33.04	49.21	19.443	4.22	3.9	1	0	B6	0.1404	0.697	0.026	1.090			
2	TbSp	L	2C	27.43	45.57	44.28	49.21	27.203	5.90	3.5	1	0	DBA	0.2240	0.685	-0.038	2.915	1		
2	TbSp	H	2C	27.43	45.57	33.04	46.53	26.998	5.86	3.7	1	0	DBA	0.1984	0.720	-0.038	11.872	1		
2	TbN	L	2C	27.43	45.57	26.56	49.66	23.306	5.06	3.7	1	0	B6	0.1760	0.700	0.317	1.735			
2	CtAr	H	2C	27.43	45.57	25.88	49.21	10.642	2.31	4	0	1	B6	0.0489	0.793	0.019	1.851			
2	BMD	RCR	2D	32.25	57.69	56.33	59.15	21.388	4.65	3.7	1	0	DBA	0.1966	0.623	-2.096	3.207			
2	TbTh	RCR	2D	32.25	57.69	56.33	61.13	14.279	3.10	3.5	0	1	DBA	0.1222	0.622	-0.240	3.060			
2	BMC	H	2E	35.73	61.96	60.79	62.41	30.109	6.55	3.6	1	0	B6	0.1621	0.800	0.034	2.298			
2	TbN	L	2E	35.88	62.41	60.79	63.74	17.432	3.78	3.7	1	0	B6	0.1379	0.662	0.286	0.010			
2	TbSp	L	2E	35.88	62.41	61.20	63.74	22.279	4.83	3.5	1	0	DBA	0.1907	0.652	-0.036	0.916			
2	CtAr	H	2E	39.59	68.75	61.13	72.42	13.561	2.95	4	0	1	B6	0.0599	0.804	0.022	1.721			
2	Imin	H	2E	40.10	69.30	64.91	72.42	12.047	2.62	3.8	0	1	B6	0.0565	0.788	0.036	0.635			
2	BMD	H	2F	43.90	73.91	72.42	74.99	10.157	2.21	3.5	0	1	DBA	0.0584	0.759	-0.015	3.163			
2	SMI	H	2G	83.83	162.90	161.41	164.37	13.882	3.01	3.9	0	1	DBA	0.0941	0.701	-0.632	3.966			
3	CtTh	RCR	3A	14.39	30.95	19.48	31.34	10.970	2.38	3.4	0	1	B6	0.0801	0.668	1.090	2.506			
3	Imax	H	3B	23.72	52.68	40.89	55.56	11.405	2.48	3.7	0	1	DBA	0.0667	0.734	-0.039	0.189		CaAbs RCR	
3	SMI	L	3B	25.13	53.57	53.16	55.56	28.997	6.29	3.7	1	0	B6	0.1648	0.785	0.781	1.446	1		
3	ConnD	L	3B	25.13	53.57	40.89	56.43	13.279	2.88	3.8	0	1	DBA	0.0578	0.806	-0.531	1.862			
3	BVTV	L	3B	25.53	54.40	34.08	56.43	10.292	2.23	3.6	0	1	DBA	0.0628	0.719	-0.020	0.607			
3	ConnD	RCR	3B	26.61	55.56	53.57	66.27	21.026	4.56	3.6	1	0	DBA	0.1707	0.666	-0.401	0.340			
3	SMI	H	3B	26.61	55.56	53.16	60.23	15.043	3.26	3.9	0	1	B6	0.0961	0.721	0.642	5.772	1		
3	BMC	L	3C	31.43	68.05	55.56	81.86	9.209	2.00	3.6	0	1	DBA	0.0409	0.793	-0.017	0.171		1,25D H	
3	BVTV	L	3C	33.24	73.62	72.35	82.89	11.737	2.55	3.6	0	1	DBA	0.1108	0.572	-0.024	1.815			
3	TbTMD	H	3C	34.02	76.06	68.05	102.64	11.585	2.51	3.5	0	1	B6	0.0884	0.653	0.012	0.246			
3	TbN	H	3D	42.84	98.67	68.05	123.88	13.502	2.93	3.6	0	1	DBA	0.0914	0.699	-0.243	5.143	1		
3	BVTV	L	3D	43.50	100.26	96.10	109.32	11.692	2.54	3.6	0	1	DBA	0.0754	0.715	-0.023	0.216			
3	TbN	L	3D	45.01	102.64	90.46	108.51	11.835	2.57	3.7	0	1	DBA	0.0623	0.762	-0.196	10.145	1		
4	MOI	H	4A	0.01	0.02	0.02	1.49	21.524	4.68	3.7	1	0	B6	0.1603	0.695	0.079	1.634			
4	Imax	H	4A	0.01	0.02	0.02	2.63	15.285	3.32	3.7	0	1	B6	0.1168	0.667	0.048	0.860	1		
4	Imax	L	4A	0.01	0.02	0.02	2.63	11.145	2.42	3.6	0	1	B6	0.0785	0.679	0.039	1.445	1		
4	BVTV	L	4B	13.20	30.27	29.71	32.45	17.935	3.89	3.6	1	0	B6	0.1212	0.713	0.025	0.373			
4	BMD	H	4C	24.22	45.66	29.71	55.58	14.498	3.15	3.5	0	1	B6	0.1276	0.628	0.022	0.806			
4	SMI	H	4C	25.40	46.77	32.35	58.11	10.641	2.31	3.9	0	1	DBA	0.0648	0.721	-0.547	5.842			
4	CATA	RCR	4C	29.00	54.21	41.36	65.24	13.165	2.86	3	0	1	DBA	0.1299	0.560	-1.093	0.040			

**4**	CATA	H	**4D**	33.84	63.12	41.36	92.00	9.391	2.04	3.7	0	1	DBA	0.0779	0.615	-0.012	1.880			
**4**	BVTV	H	**4E**	36.61	75.09	65.24	92.00	14.171	3.07	3.9	0	1	B6	0.0971	0.697	0.022	1.088			1
**4**	SMI	L	**4E**	40.13	84.84	83.76	97.12	22.815	4.95	3.7	1	0	DBA	0.1413	0.758	-0.753	0.513			
**4**	ConnD	RCR	**4E**	40.13	84.84	74.50	89.51	13.816	3.00	3.6	0	1	B6	0.1046	0.664	0.294	0.641			
**4**	TbSp	H	**4E**	40.13	84.84	81.54	92.00	13.170	2.86	3.7	0	1	DBA	0.0966	0.673	-0.027	7.863			
**4**	ConnD	L	**4E**	41.49	88.17	82.46	92.00	29.195	6.33	3.8	1	0	B6	0.2101	0.728	1.046	0.347	1		
**4**	ConnD	H	**4E**	41.49	88.17	82.46	92.00	26.986	5.85	3.8	1	0	B6	0.2231	0.680	1.097	1.565	1		
**4**	TbN	H	**4E**	41.49	88.17	81.54	92.00	13.031	2.83	3.6	0	1	B6	0.0913	0.687	0.239	3.527			
**4**	BMC	RCR	**4F**	48.50	104.70	100.96	117.65	11.285	2.45	3.6	0	1	DBA	0.0933	0.624	-2.298	0.438		CaAbs H	
**4**	CtTh	RCR	**4F**	48.72	104.84	92.00	105.08	11.471	2.49	3.4	0	1	DBA	0.1470	0.455	-1.407	15.386			
**4**	MOI	H	**4G**	61.47	127.30	122.88	137.51	13.241	2.88	3.7	0	1	DBA	0.0958	0.678	-0.062	1.969			
**4**	CtTh	H	**4G**	66.90	134.54	130.35	136.55	20.363	4.43	3.8	1	0	DBA	0.1769	0.640	-0.008	0.262			
**4**	Imax	H	**4G**	67.09	134.74	126.07	137.51	17.862	3.88	3.7	1	0	DBA	0.1311	0.688	-0.051	0.445			
**4**	Imin	RCR	**4H**	68.84	136.55	132.02	142.58	9.606	2.09	3	0	1	B6	0.0811	0.609	2.056	0.350			
**4**	TtAr	RCR	**4H**	69.94	137.51	134.74	149.68	12.318	2.68	3.6	0	1	B6	0.0708	0.741	0.103	0.652			
**4**	MOI	L	**4H**	74.55	141.58	136.55	143.19	12.467	2.71	3.7	0	1	DBA	0.0750	0.733	-0.060	1.841			
**4**	MOI	RCR	**4H**	74.55	141.58	136.19	149.68	12.200	2.65	3.4	0	1	B6	0.0824	0.696	0.233	1.589			
**4**	TbTMD	L	**4H**	74.55	141.58	136.55	149.68	10.925	2.37	3.7	0	1	B6	0.0699	0.708	0.009	0.995			
**4**	TbTMD	RCR	**4H**	76.30	142.88	141.58	148.15	25.198	5.47	3.5	1	0	B6	0.2411	0.623	1.430	13.445			
**4**	TtAr	H	**4I**	86.49	155.43	151.12	156.18	10.594	2.30	3.7	0	1	DBA	0.0740	0.680	-0.029	0.264			
5	BMC	H	5A	8.35	18.67	16.98	20.00	19.872	4.32	3.6	1	0	DBA	0.0956	0.800	-0.027	2.293			
5	TbN	RCR	5B	40.86	76.30	70.59	89.70	9.365	2.03	3.9	0	1	DBA	0.0598	0.703	-1.449	3.010			
5	TbTMD	L	5B	44.69	90.80	76.45	92.74	24.133	5.23	3.7	1	0	DBA	0.1740	0.713	-0.015	0.954			
5	TbTh	L	5C	51.36	106.44	87.77	112.21	9.261	2.01	3.5	0	1	B6	0.0569	0.716	0.012	0.261			
5	CATA	H	5C	51.79	107.07	103.17	113.61	23.890	5.19	3.7	1	0	DBA	0.2319	0.612	-0.020	2.634			
5	BMD	H	5C	54.14	111.60	106.91	112.34	23.311	5.07	3.5	1	0	DBA	0.1736	0.701	-0.028	0.513			
5	CtTh	H	5D	54.69	112.34	111.60	117.88	27.122	5.90	3.8	1	0	DBA	0.2604	0.629	-0.010	0.714	1		
5	CtTh	L	5D	54.69	112.34	111.60	117.88	21.323	4.64	3.9	1	0	DBA	0.2018	0.611	-0.009	0.467	1		
5	MOI	L	5D	55.58	113.61	111.60	123.43	8.996	1.96	3.7	0	1	B6	0.0433	0.775	0.044	1.020			
5	BVTV	L	5D	66.51	127.89	118.18	130.28	10.429	2.26	3.6	0	1	DBA	0.0549	0.768	-0.017	5.966			
5	CtAr	L	5E	77.86	139.01	134.04	151.62	9.404	2.04	3.8	0	1	B6	0.0444	0.781	0.018	7.486			
**6**	BVTV	RCR	**6A**	2.00	5.30	0.02	7.26	10.290	2.23	3.7	0	1	DBA	0.0675	0.699	-0.092	0.960			
**6**	ConnD	RCR	**6A**	3.00	7.26	0.02	13.02	12.046	2.61	3.6	0	1	DBA	0.0876	0.671	-0.284	0.453			
**6**	CtAr	L	**6B**	6.56	14.98	10.71	23.86	13.085	2.84	3.8	0	1	DBA	0.0608	0.797	-0.021	3.771			
**6**	BMC	L	**6B**	9.13	22.21	16.41	22.77	24.070	5.23	3.6	1	0	DBA	0.1504	0.752	-0.034	1.036			
**6**	BMD	L	**6B**	9.13	22.21	15.22	22.77	22.640	4.92	3.6	1	0	DBA	0.1622	0.711	-0.026	2.935			
**6**	CtAr	L	**6B**	9.13	22.21	10.71	23.86	12.637	2.75	3.8	0	1	DBA	0.0580	0.794	-0.021	3.323			
**6**	Imin	H	**6B**	9.81	22.78	16.41	23.86	11.813	2.57	3.8	0	1	DBA	0.0720	0.730	-0.041	1.054			
**6**	BMC	H	**6C**	13.59	32.52	16.41	59.10	10.478	2.28	3.6	0	1	DBA	0.0372	0.837	-0.018	10.034			
**6**	TbN	RCR	**6D**	24.98	51.64	47.77	59.10	10.422	2.26	3.9	0	1	DBA	0.0810	0.643	-1.629	1.756		25OHD H/L	
**6**	SMI	H	**6E**	42.98	94.22	92.97	94.78	25.699	5.57	3.9	1	0	DBA	0.1833	0.721	-0.952	5.843		CaAbs L	
**6**	TbSp	RCR	**6E**	44.15	95.10	94.22	109.79	19.806	4.30	3.6	1	0	B6	0.1349	0.716	2.981	1.120			
**6**	TbN	RCR	**6E**	44.15	95.10	93.68	101.73	13.243	2.87	3.9	0	1	DBA	0.1059	0.643	-1.793	1.759			
**6**	CATA	RCR	**6F**	64.55	133.98	127.38	141.74	11.956	2.60	3	0	1	DBA	0.0849	0.679	-0.705	1.232			
**6**	Imin	L	**6F**	65.24	134.40	125.93	142.83	8.938	1.94	3.6	0	1	B6	0.0352	0.816	0.027	2.419			
7	TbN	L	7A	0.34	0.56	0.02	2.97	15.670	3.40	3.7	0	1	B6	0.1083	0.699	0.280	1.682			
7	TbSp	L	7A	0.34	0.56	0.02	2.97	14.917	3.24	3.5	0	1	DBA	0.1070	0.686	-0.030	2.758			
7	BMD	RCR	7B	11.20	24.45	12.72	25.66	11.612	2.52	3.7	0	1	B6	0.0966	0.623	1.655	3.198			
7	ConnD	H	7B	11.20	24.45	11.45	25.66	9.463	2.05	3.8	0	1	DBA	0.0507	0.752	-0.580	3.143			
7	ConnD	H	7C	18.52	30.52	25.66	46.26	9.576	2.08	3.8	0	1	DBA	0.0512	0.752	-0.580	0.468			
7	CATA	H	7C	23.71	37.05	35.21	37.61	20.499	4.46	3.7	1	0	DBA	0.1904	0.615	-0.020	1.879	1		
7	CATA	L	7C	23.71	37.05	35.21	39.98	14.822	3.22	4	0	1	DBA	0.1453	0.569	-0.019	0.594	1		
7	CtAr	L	7D	29.87	46.26	37.05	57.91	12.656	2.75	3.8	0	1	DBA	0.0753	0.734	-0.024	7.944		1,25D L	
7	TbTMD	H	7D	31.58	49.77	48.68	65.32	28.379	6.16	3.5	1	0	DBA	0.2626	0.651	-0.022	0.234			
7	MOI	H	7D	31.58	49.77	40.48	66.87	9.902	2.15	3.7	0	1	DBA	0.0554	0.742	-0.049	6.011			
7	CtAr	H	7D	35.08	65.32	49.80	66.47	33.005	7.17	4	1	0	DBA	0.2335	0.744	-0.051	0.924			

7	CtAr	L	7D	35.68	66.35	58.02	67.29	15.451	3.36	3.8	0	1	DBA	0.0894	0.748	-0.029	0.959			
7	TbTh	H	7D	37.99	69.16	48.68	72.60	17.464	3.79	3.6	1	0	DBA	0.1414	0.686	-0.020	3.510			
7	BVTV	RCR	7D	38.04	69.35	48.68	75.92	10.524	2.28	3.7	0	1	B6	0.1193	0.480	0.120	2.005			
7	BVTV	H	7E	39.93	70.87	66.35	75.92	12.950	2.81	3.9	0	1	DBA	0.0679	0.765	-0.019	0.701			
7	CtTh	RCR	7E	41.07	71.76	70.87	74.18	23.141	5.03	3.4	1	0	B6	0.1911	0.668	1.753	2.478			
7	TbTMD	L	7F	61.83	116.49	110.42	125.88	10.041	2.18	3.7	0	1	B6	0.0525	0.761	0.008	2.400			
8	TtAr	L	8A	1.03	1.99	0.02	6.28	26.407	5.74	3.7	1	0	B6	0.1886	0.722	0.046	1.118	1		
8	TtAr	H	8A	1.03	1.99	0.02	8.06	15.255	3.32	3.7	0	1	B6	0.1115	0.680	0.035	0.244	1		
8	TbTMD	RCR	8A	1.03	1.99	0.02	11.98	14.615	3.17	3.5	0	1	B6	0.1252	0.623	1.013	13.449			
8	Imax	L	8B	4.89	10.89	0.02	14.34	16.242	3.53	3.6	0	1	B6	0.1256	0.673	0.050	0.760			
8	BVTV	L	8B	8.28	15.34	10.89	25.07	11.622	2.52	3.6	0	1	DBA	0.0586	0.771	-0.017	0.350			
8	TtAr	RCR	8C	20.55	33.51	26.34	48.51	10.784	2.34	3.6	0	1	DBA	0.0777	0.670	-0.113	3.915		CaAbs RCR	1
8	TbSp	RCR	8C	22.60	36.19	26.98	54.95	21.437	4.65	3.6	1	0	B6	0.1474	0.718	3.104	0.651			
8	TbN	RCR	8C	23.75	39.95	35.68	48.51	23.612	5.12	3.9	1	0	DBA	0.2103	0.643	-2.445	1.757			
8	BMD	RCR	8C	24.71	44.77	35.68	49.55	10.373	2.26	3.7	0	1	DBA	0.0825	0.634	-1.239	2.067			
8	SMI	RCR	8C	27.72	49.48	44.77	49.55	18.304	3.97	3.4	1	0	B6	0.1553	0.649	8.014	4.684			
8	BMC	RCR	8C	27.72	49.48	45.29	49.55	14.730	3.20	3.6	0	1	DBA	0.1610	0.534	-2.939	2.692			
8	ConnD	L	8D	30.15	57.65	49.48	68.65	12.169	2.64	3.8	0	1	B6	0.0536	0.802	0.505	0.537			
8	SMI	RCR	8D	33.75	68.71	62.48	77.07	14.861	3.22	3.4	0	1	B6	0.1282	0.621	7.132	9.033			
8	CATA	H	8D	36.45	77.07	68.71	84.15	17.617	3.83	3.7	1	0	DBA	0.1570	0.620	-0.017	2.996			
8	MOI	L	8E	42.29	87.59	84.15	92.11	17.681	3.84	3.7	1	0	B6	0.1115	0.732	0.065	1.871			
8	BMC	L	8E	46.69	94.73	84.94	95.76	10.103	2.20	3.6	0	1	B6	0.0445	0.797	0.025	2.838			
9	TbTMD	L	9A	17.08	31.83	31.04	32.31	43.485	9.43	3.7	1	0	B6	0.3935	0.708	0.024	0.982		1,25D H/L	1
9	ConnD	L	9A	18.37	34.44	29.82	38.27	18.228	3.95	3.8	1	0	DBA	0.1092	0.746	-0.680	0.169	1		
9	ConnD	H	9A	19.19	34.89	31.83	40.50	17.553	3.81	3.8	1	0	DBA	0.1232	0.701	-0.766	1.906	1		
9	TbSp	H	9A	20.19	35.37	29.82	40.50	21.271	4.61	3.7	1	0	B6	0.1560	0.699	0.035	11.493	1		
9	TbSp	L	9A	20.84	38.27	35.05	40.50	25.206	5.47	3.5	1	0	B6	0.2010	0.686	0.041	2.759	1		
9	TbN	L	9A	20.84	38.27	35.05	41.09	22.087	4.79	3.7	1	0	DBA	0.1630	0.699	-0.314	1.683	1		
9	TbN	H	9A	20.84	38.27	34.65	42.80	18.725	4.06	3.6	1	0	DBA	0.1336	0.699	-0.297	5.147			
9	TbN	H	9A	24.44	43.83	27.32	50.22	10.191	2.21	3.6	0	1	DBA	0.0790	0.644	-0.227	4.611	1		
9	TbTMD	L	9B	53.46	101.50	97.14	103.90	24.179	5.24	3.7	1	0	DBA	0.1771	0.708	-0.015	0.982		25OHD L	
9	MOI	L	9C	59.16	108.08	105.80	110.57	32.234	7.01	3.7	1	0	DBA	0.2362	0.732	-0.096	1.855			
9	Imin	L	9C	60.04	110.50	105.80	110.57	32.298	7.02	3.6	1	0	DBA	0.1599	0.822	-0.059	4.078	1		
9	Imin	H	9C	60.04	110.50	104.01	115.06	11.155	2.42	3.8	0	1	DBA	0.0567	0.788	-0.035	0.674	1		
9	TbTh	RCR	9D	64.92	114.67	112.62	116.61	26.021	5.64	3.5	1	0	DBA	0.2609	0.687	-0.351	1.563			1
9	BVTV	RCR	9D	66.78	115.06	112.62	116.61	19.379	4.20	3.7	1	0	DBA	0.1704	0.632	-0.131	1.147			
9	ConnD	RCR	9D	67.54	115.15	114.67	116.61	20.814	4.51	3.6	1	0	DBA	0.1696	0.664	-0.371	0.642			
9	BMC	H	9E	70.67	118.85	115.15	121.12	10.911	2.37	3.6	0	1	B6	0.0387	0.838	0.017	1.930			
9	BMD	RCR	9E	72.31	121.12	115.06	124.12	10.369	2.25	3.7	0	1	DBA	0.0842	0.707	-1.307	1.181			
10	MOI	RCR	10A	27.20	53.52	41.47	64.14	9.061	1.97	3.4	0	1	B6	0.0630	0.677	0.226	0.589			
10	TbTh	L	10A	30.44	60.40	45.95	65.86	9.740	2.11	3.5	0	1	B6	0.0685	0.727	0.013	3.769			
10	CtTh	L	10B	42.07	86.13	73.37	94.92	9.628	2.09	3.9	0	1	DBA	0.0818	0.610	-0.005	0.471			
10	TtAr	RCR	10C	54.14	103.58	98.28	109.84	11.074	2.41	3.6	0	1	B6	0.0644	0.734	0.105	0.061		1,25D RCR	
10	ConnD	L	10C	57.28	109.84	107.87	112.20	10.078	2.19	3.8	0	1	B6	0.0457	0.794	0.490	0.799		CaAbs H	
10	ConnD	L	10C	64.56	116.11	112.20	130.42	9.679	2.10	3.8	0	1	B6	0.0435	0.792	0.449	0.379			
10	SMI	H	10D	69.10	121.17	119.97	125.61	10.699	2.32	3.9	0	1	DBA	0.0530	0.774	-0.509	6.316			
10	CtAr	H	10D	71.78	123.76	121.07	130.42	10.930	2.38	4	0	1	DBA	0.0613	0.744	-0.023	0.925			
11	Imin	RCR	11A	10.53	18.16	8.48	31.70	9.498	2.06	3	0	1	B6	0.0795	0.612	2.100	0.547			
11	TtAr	RCR	11A	13.21	20.67	17.26	29.99	19.680	4.28	3.6	1	0	B6	0.1555	0.670	0.151	3.969			
11	BMD	RCR	11A	13.21	20.67	17.26	31.70	9.955	2.16	3.7	0	1	B6	0.0669	0.690	1.183	7.631			
11	MOI	RCR	11A	15.73	26.24	16.73	34.13	10.187	2.21	3.4	0	1	B6	0.0871	0.608	0.240	1.946			
11	CtAr	RCR	11B	37.38	59.28	58.29	59.33	16.264	3.54	3.6	0	1	B6	0.2173	0.433	2.523	10.811			
11	SMI	L	11C	54.48	89.48	83.10	91.30	20.004	4.34	3.7	1	0	B6	0.1119	0.802	0.682	1.083			
11	SMI	L	11D	70.47	107.86	105.04	109.60	20.572	4.46	3.7	1	0	DBA	0.1271	0.841	-0.684	0.216			
12	Imax	RCR	12A	2.01	4.07	0.02	10.57	20.081	4.37	3.4	1	0	DBA	0.1682	0.664	-0.288	2.528		CaAbs H/L	
12	CtAr	RCR	12A	2.01	4.07	0.02	12.34	13.256	2.88	3.6	0	1	DBA	0.1152	0.623	-1.830	0.706			

12	TbTh	L	12A	4.51	9.83	8.15	10.57	18.864	4.09	3.5	1	0	DBA	0.1561	0.662	-0.019	0.309			
12	BVTV	RCR	12A	5.43	10.57	8.15	16.74	16.764	3.64	3.7	0	1	DBA	0.1443	0.631	-0.123	1.132			
12	MOI	RCR	12A	6.39	13.30	4.07	26.54	17.290	3.76	3.4	1	0	DBA	0.1559	0.614	-0.328	2.207			
12	TtAr	RCR	12B	7.86	16.54	16.03	26.54	28.834	6.27	3.6	1	0	DBA	0.2441	0.680	-0.195	3.234			
12	Imin	RCR	12B	7.86	16.54	0.02	26.54	11.446	2.49	3	0	1	DBA	0.1518	0.397	-2.967	46.814			
12	Imax	L	12B	8.06	17.11	16.03	27.46	16.767	3.65	3.6	1	0	B6	0.1268	0.674	0.048	0.950	1		
12	MOI	H	12B	8.69	25.15	16.03	28.06	23.413	5.09	3.7	1	0	B6	0.1826	0.687	0.082	2.115			
12	Imax	H	12B	8.69	25.15	16.03	27.46	19.440	4.23	3.7	1	0	B6	0.1545	0.667	0.055	0.841	1		
12	TbTh	L	12C	10.62	28.06	27.46	32.93	15.334	3.33	3.5	0	1	DBA	0.1284	0.634	-0.017	0.467			
12	TtAr	RCR	12C	14.03	33.15	32.41	42.59	17.009	3.70	3.6	1	0	DBA	0.1600	0.596	-0.156	1.920			
12	TbSp	RCR	12C	19.22	42.39	33.83	44.34	10.590	2.30	3.6	0	1	DBA	0.0552	0.771	-1.984	1.197			
12	Imax	RCR	12D	31.03	73.66	43.92	73.97	10.559	2.30	3.4	0	1	B6	0.0643	0.724	0.183	2.335			
12	TbTh	L	12D	36.22	79.72	65.53	81.61	9.555	2.07	3.5	0	1	DBA	0.0583	0.717	-0.013	2.603			
12	TtAr	L	12E	42.23	88.03	83.44	97.78	10.019	2.18	3.7	0	1	DBA	0.0603	0.722	-0.028	1.121			
13	TbN	L	13A	12.54	27.59	25.33	37.63	10.252	2.22	3.7	0	1	DBA	0.0548	0.754	-0.175	1.176		25OHD L	
13	TbSp	RCR	13A	22.99	46.05	37.66	52.91	10.991	2.38	3.6	0	1	B6	0.0552	0.771	1.889	1.711			
13	BVTV	H	13B	31.20	58.81	55.91	64.61	17.089	3.71	3.9	0	1	DBA	0.1335	0.667	-0.034	0.190			
13	TbTMD	L	13C	39.17	72.57	68.56	81.16	26.697	5.79	3.7	1	0	DBA	0.2004	0.709	-0.015	1.955			
13	BVTV	H	13C	42.99	81.42	81.28	97.69	10.922	2.37	3.9	0	1	B6	0.0722	0.697	0.023	1.087			
13	ConnD	L	13D	62.97	110.87	110.08	119.24	14.711	3.19	3.8	0	1	DBA	0.0861	0.748	-0.623	0.181			
14	TbSp	H	14A	4.30	7.37	0.02	22.59	11.186	2.43	3.7	0	1	DBA	0.0766	0.688	-0.024	10.550			
14	TbTMD	H	14B	18.86	30.65	29.89	36.51	15.471	3.36	3.5	0	1	B6	0.1230	0.653	0.014	0.243			
14	BMC	H	14C	23.79	46.18	44.96	46.52	23.893	5.19	3.6	1	0	DBA	0.1154	0.809	-0.030	2.776			
14	TbTMD	RCR	14D	60.43	117.00	102.32	118.17	11.073	2.40	3.5	0	1	B6	0.0916	0.623	0.886	13.402			
14	TbN	RCR	14D	62.96	119.50	117.09	124.69	14.317	3.11	3.9	0	1	DBA	0.1159	0.642	-1.834	2.413			
14	ConnD	RCR	14D	62.96	119.50	117.09	124.69	12.946	2.81	3.6	0	1	DBA	0.1010	0.651	-0.283	0.633			
15	TbSp	L	15A	1.75	3.45	0.53	6.19	29.758	6.45	3.5	1	0	DBA	0.2502	0.685	-0.042	2.795	1		1
15	TbN	L	15A	1.75	3.45	0.53	6.19	25.590	5.55	3.7	1	0	B6	0.1971	0.699	0.337	1.682	1		
15	TbN	H	15A	1.75	3.45	0.53	6.80	21.646	4.70	3.7	1	0	B6	0.1598	0.699	0.319	5.148	1		
15	TbSp	H	15A	1.75	3.45	0.53	6.19	19.505	4.23	3.7	1	0	DBA	0.1460	0.687	-0.032	10.551	1		
15	BVTV	H	15A	1.75	3.45	0.53	12.11	18.342	3.98	3.9	1	0	B6	0.1314	0.697	0.025	1.085			
15	BMC	H	15A	3.25	6.80	6.19	11.79	24.992	5.43	3.6	1	0	B6	0.1951	0.760	0.037	1.832			
15	CtAr	H	15A	3.25	6.80	0.53	9.73	22.680	4.93	4	1	0	B6	0.1891	0.687	0.041	0.362			
15	TtAr	RCR	15B	13.56	32.33	30.19	59.80	9.732	2.12	3.6	0	1	B6	0.0574	0.727	0.097	2.769			
15	BMC	L	15B	14.72	37.08	32.44	39.93	38.427	8.35	3.6	1	0	B6	0.2790	0.752	0.045	1.038			
15	CtAr	L	15C	28.61	64.15	59.80	78.30	15.895	3.46	3.8	0	1	B6	0.1122	0.694	0.029	3.416		CaAbs RCR	1
15	Imax	H	15C	29.04	65.05	64.11	72.03	26.716	5.81	3.7	1	0	B6	0.2292	0.667	0.073	0.840			
15	TtAr	H	15C	29.04	65.05	59.80	69.65	26.410	5.74	3.7	1	0	B6	0.2169	0.681	0.050	0.245	1		
15	Imin	H	15C	29.04	65.05	63.77	72.03	26.015	5.66	3.8	1	0	B6	0.1787	0.732	0.064	1.025			
15	TtAr	L	15C	29.04	65.05	63.61	69.08	25.228	5.48	3.7	1	0	B6	0.1780	0.722	0.046	1.120	1		
15	MOI	H	15C	29.04	65.05	64.11	72.03	22.764	4.95	3.7	1	0	B6	0.1764	0.687	0.089	2.113			
15	TbTh	H	15C	32.40	71.95	69.65	72.53	35.089	7.61	3.6	1	0	B6	0.3268	0.670	0.027	3.397	1		
15	TbTh	L	15C	32.40	71.95	69.65	72.53	30.326	6.58	3.5	1	0	B6	0.2798	0.659	0.026	0.336	1		
15	CtAr	L	15C	32.40	71.95	69.65	72.53	23.347	5.08	3.8	1	0	B6	0.1544	0.736	0.034	9.651			
15	BMD	L	15C	32.40	71.95	69.08	72.03	18.081	3.93	3.6	1	0	B6	0.1248	0.711	0.025	2.946			
15	SMI	L	15C	32.49	72.03	69.65	72.53	44.049	9.56	3.7	1	0	DBA	0.2962	0.785	-1.069	1.452	1		
15	BVTV	L	15C	32.49	72.03	69.65	72.53	37.651	8.17	3.6	1	0	B6	0.3148	0.713	0.041	0.375			
15	SMI	H	15C	32.49	72.03	69.65	72.53	27.947	6.06	3.9	1	0	DBA	0.2044	0.721	-0.903	5.996	1		
15	MOI	L	15D	37.36	78.30	77.96	85.29	30.867	6.71	3.7	1	0	B6	0.2287	0.732	0.102	1.670		25OHD L	
15	Imin	L	15D	39.39	83.40	78.30	85.29	40.682	8.84	3.6	1	0	B6	0.2238	0.817	0.069	2.472			
15	Imin	L	15D	43.22	88.21	86.47	88.92	27.091	5.89	3.6	1	0	B6	0.1679	0.761	0.061	4.628			
16	TbTh	L	16A	3.70	8.28	0.02	17.18	10.357	2.25	3.5	0	1	B6	0.0627	0.722	0.012	2.178			
16	CATA	L	16B	34.17	56.96	31.83	58.60	12.301	2.67	4	0	1	DBA	0.1184	0.568	-0.015	0.877			
16	TtAr	RCR	16C	37.86	66.45	63.33	69.64	13.032	2.83	3.6	0	1	B6	0.1066	0.665	0.133	4.145			
16	BMC	RCR	16C	38.13	66.76	31.83	72.15	9.030	1.96	3.6	0	1	B6	0.0929	0.521	2.224	1.440			
16	SMI	RCR	16C	38.13	66.76	66.45	72.15	14.619	3.17	3.4	0	1	DBA	0.1164	0.650	-6.948	4.787			

**16**	MOI	RCR	**16C**	39.80	69.29	64.34	69.64	13.641	2.97	3.4	0	1	B6	0.1196	0.616	0.289	1.552			
**16**	BMD	RCR	**16D**	51.74	90.50	85.80	90.71	12.028	2.61	3.7	0	1	B6	0.0798	0.703	1.224	0.157			
17	TbSp	RCR	17A	0.27	0.42	0.02	2.80	21.641	4.69	3.6	1	0	DBA	0.1569	0.703	-3.201	0.576			
17	TbTh	H	17A	2.92	5.07	0.02	18.20	10.984	2.38	3.6	0	1	DBA	0.0638	0.735	-0.012	1.152			
17	CtAr	RCR	17A	6.40	9.26	0.42	28.92	10.068	2.19	3.6	0	1	DBA	0.0678	0.692	-1.390	0.269			
17	CATA	L	17B	12.65	25.27	18.20	28.84	16.771	3.65	4	0	1	DBA	0.1682	0.568	-0.018	0.884			
17	TbTMD	RCR	17C	52.75	83.37	78.80	84.01	20.642	4.48	3.5	1	0	DBA	0.1881	0.623	-1.251	13.454			
**18**	Imin	L	**18A**	0.01	0.02	0.02	4.78	16.656	3.62	3.6	1	0	B6	0.0709	0.817	0.040	2.470	1		
**18**	CtAr	L	**18A**	2.48	4.02	0.02	25.78	14.472	3.15	3.8	0	1	B6	0.0903	0.725	0.025	5.498			
**18**	Imin	H	**18A**	6.94	13.09	5.35	18.53	20.287	4.41	3.8	1	0	B6	0.1709	0.650	0.061	1.180	1		
**18**	CtAr	L	**18B**	6.94	13.09	0.02	18.53	16.622	3.61	3.8	0	1	B6	0.1017	0.736	0.027	9.712	1		
**18**	SMI	RCR	**18B**	7.85	14.03	7.23	14.15	20.524	4.45	3.4	1	0	B6	0.1936	0.668	9.574	3.108			
**18**	TtAr	H	**18B**	7.89	14.15	13.20	18.53	32.205	7.00	3.7	1	0	B6	0.2813	0.681	0.056	0.245	1		
**18**	TtAr	L	**18B**	7.89	14.15	13.20	25.70	29.922	6.50	3.7	1	0	B6	0.2217	0.722	0.049	1.119	1		
**18**	CtAr	H	**18B**	10.91	19.27	13.20	25.78	20.409	4.44	4	1	0	B6	0.1261	0.744	0.033	0.924	1		
**18**	Imax	RCR	**18C**	18.89	35.12	34.13	37.86	27.029	5.88	3.4	1	0	B6	0.2384	0.660	0.337	2.362			1
**18**	SMI	RCR	**18C**	18.89	35.12	29.98	37.91	18.635	4.04	3.4	1	0	DBA	0.1554	0.649	-8.492	4.786			
**18**	TbTh	RCR	**18C**	18.89	35.12	25.20	42.29	9.437	2.05	3.5	0	1	B6	0.0932	0.546	0.206	6.572			
**18**	TtAr	RCR	**18C**	20.87	38.88	35.45	38.94	24.705	5.37	3.6	1	0	B6	0.2098	0.669	0.179	3.961			
**18**	MOI	RCR	**18C**	20.87	38.88	35.45	38.94	22.288	4.85	3.4	1	0	B6	0.2142	0.613	0.387	2.343			
**18**	Imax	RCR	**18C**	21.90	41.76	38.84	45.52	27.207	5.91	3.4	1	0	B6	0.2392	0.661	0.348	1.562			
**18**	CtAr	RCR	**18C**	21.90	41.76	34.13	47.08	23.591	5.13	3.6	1	0	B6	0.2179	0.630	2.649	1.089			
**18**	SMI	RCR	**18C**	21.90	41.76	38.88	45.52	22.271	4.83	3.4	1	0	DBA	0.1791	0.673	-9.354	4.116			
**18**	CATA	RCR	**18D**	29.69	55.53	54.77	57.91	9.935	2.16	3	0	1	DBA	0.1223	0.432	-0.893	19.117			
**18**	TbN	L	**18E**	36.67	63.26	58.40	68.07	11.518	2.50	3.7	0	1	B6	0.0608	0.760	0.205	0.563			
**18**	ConnD	L	**18E**	37.68	64.87	59.03	65.63	26.687	5.79	3.8	1	0	B6	0.1737	0.748	0.928	0.179	1		
**18**	ConnD	H	**18E**	38.86	65.60	58.40	68.37	21.794	4.73	3.8	1	0	B6	0.1638	0.698	0.910	1.730	1		
**18**	CtAr	L	**18F**	49.81	73.73				2.78		0	1	DBA	0.0585	0.795	-0.022	3.898		1,25D H/L	
**18**	CtTh	L	**18F**	50.59	74.34	73.69	78.93	15.901	3.46	3.9	0	1	DBA	0.1427	0.611	-0.007	0.465		FGF23	
**18**	MOI	RCR	**18F**	50.59	74.34	73.69	80.44	9.093	1.98	3.4	0	1	DBA	0.0757	0.613	-0.229	2.219			
19	Imax	L	19A	2.95	3.18	1.13	8.74	12.760	2.77	3.6	0	1	B6	0.0721	0.746	0.038	0.222			
19	TbTMD	L	19B	7.72	11.12	6.43	23.96	9.924	2.15	3.7	0	1	B6	0.0516	0.760	0.008	0.535			
19	SMI	H	19C	23.96	29.37	28.90	30.11	14.666	3.18	3.9	0	1	B6	0.0700	0.791	0.541	4.201			
**20**	TtAr	L	**20A**	2.79	6.12	0.02	19.71	16.244	3.53	3.7	0	1	B6	0.1042	0.722	0.039	1.120			
**20**	Imax	L	**20B**	34.80	67.44	20.41	93.74	8.832	1.92	3.6	0	1	B6	0.0518	0.726	0.036	0.112			
**20**	CtAr	L	**20C**	55.71	134.05	128.34	138.23	31.672	6.89	3.8	1	0	B6	0.2304	0.733	0.041	9.619	1		1
**20**	Imax	L	**20C**	56.07	134.35	128.34	140.89	16.643	3.62	3.6	1	0	B6	0.1281	0.685	0.051	0.725			
**20**	Imin	L	**20C**	56.11	134.39	134.05	137.65	45.086	9.80	3.6	1	0	B6	0.2570	0.819	0.073	3.294	1		
**20**	CtAr	H	**20C**	56.11	134.39	134.05	138.23	38.007	8.26	4	1	0	B6	0.2786	0.748	0.049	0.963	1		
**20**	MOI	L	**20C**	56.11	134.39	131.61	140.89	28.346	6.16	3.7	1	0	B6	0.1963	0.736	0.084	1.813			
**20**	Imin	H	**20C**	58.89	137.16	134.35	140.89	37.030	8.05	3.8	1	0	B6	0.2789	0.739	0.084	0.804	1		
**20**	CtAr	L	**20C**	58.89	137.16	131.61	138.23	32.371	7.04	3.8	1	0	B6	0.2339	0.736	0.041	9.655			
**20**	BMC	H	**20C**	60.71	138.23	137.16	140.89	44.803	9.74	3.6	1	0	B6	0.2824	0.800	0.044	2.300	1		
**20**	TbTh	H	**20C**	61.96	140.89	138.23	141.90	30.014	6.51	3.6	1	0	B6	0.2641	0.671	0.025	3.348	1		
**20**	BMC	L	**20C**	61.96	140.89	138.23	141.90	24.798	5.39	3.6	1	0	B6	0.1555	0.752	0.035	1.035	1		
**20**	BMD	L	**20C**	61.96	140.89	137.16	141.90	20.707	4.50	3.6	1	0	B6	0.1450	0.711	0.025	2.934			
**20**	TbTh	L	**20C**	61.96	140.89	138.23	141.90	18.275	3.96	3.5	1	0	B6	0.2028	0.530	0.021	22.365	1		
**20**	Imin	L	**20C**	68.58	150.41	144.72	151.57	21.584	4.69	3.6	1	0	B6	0.1512	0.713	0.056	0.271			

* QTL Reported for Calcium absorption (CaAbs), serum 1,25$(OH)_2$ D (1,25D), 25OHD, or FGF in Fleet et al. (2016) Endocrinology 157:470 or Reyes Fernandez et al. (2016) J Bone Miner Res 31:994

Supplemental Table S6. Pearson Correlation Coefficients of Body Size-corrected Line Means of Femoral Bone Traits in Each Diet Group and in RCR.

	**BMD_Basal**	**BMD_LowCa**	**BMD_RCR**	**BMC_Basal**	**BMC_LowCa**	**BMC_RCR**	**BV/TV_Basal**	**BV/TV_LowCa**	**BV/TV_RCR**	**Tb.N_Basal**	**Tb.N_LowCa**	**Tb.N_RCR**	**Tb.Th_Basal**
**BMD_Basal**		0.8511	-0.1712	0.9341	0.7958	-0.1476	0.6945	0.6118	-0.0875	0.4856	0.4801	0.0247	0.6561
**BMD_LowCa**			0.3247	0.7952	0.9210	0.2550	0.5237	0.6726	0.2793	0.3810	0.4492	0.2361	0.4886
**BMD_RCR**				-0.1487	0.2774	0.9026	-0.2228	0.1843	0.7348	-0.1267	-0.0039	0.4385	-0.2326
**BMC_Basal**					0.8201	-0.1789	0.6525	0.5797	-0.0792	0.4406	0.4231	-0.0108	0.6364
**BMC_LowCa**						0.2911	0.4955	0.5964	0.1872	0.3508	0.3686	0.1004	0.4758
**BMC_RCR**							-0.1941	0.1181	0.6103	-0.1088	-0.0365	0.3414	-0.2017
**BV/TV_Basal**								0.8290	-0.1611	0.8306	0.7721	-0.0944	0.6727
**BV/TV_LowCa**									0.3906	0.7250	0.7908	0.2549	0.5331
**BV/TV_RCR**										-0.0509	0.1329	0.5990	-0.1916
**Tb.N_Basal**											0.9360	-0.1103	0.1773
**Tb.N_LowCa**												0.2317	0.1701
**Tb.N_RCR**													0.0198
**Tb.Th_Basal**													
**Tb.Th_LowCa**													
**Tb.Th_RCR**													
**Tb.Sp_Basal**													
**Tb.Sp_LowCa**													
**Tb.Sp_RCR**													
**Conn.D_Basal**													
**Conn.D_LowCa**													
**Conn.D_RCR**													
**SMI_Basal**													
**SMI_LowCa**													
**SMI_RCR**													
**Tb.TMD_Basal**													
**Tb.TMD_LowCa**													
**Tb.TMD_RCR**													
**Ct.Ar_Basal**													
**Ct.Ar_LowCa**													
**Ct.Ar_RCR**													
**Tt.Ar_Basal**													
**Tt.Ar_LowCa**													
**Tt.Ar_RCR**													
**Ct.Ar/Tt.Ar_Basal**													
**Ct.Ar/Tt.Ar_LowCa**													
**Ct.Ar/Tt.Ar_RCR**													
**Ct.Th_Basal**													
**Ct.Th_LowCa**													
**Ct.Th_RCR**													
***J*_Basal**													
***J*_LowCa**													
***J*_RCR**													
***Imax*_Basal**													
***Imax*_LowCa**													
***Imax*_RCR**													
***Imin*_Basal**													
***Imin*_LowCa**													
***Imin*_RCR**													

Abbreviation: BMD = bone mineral density (g/cm^2); BMC = bone mineral content (g); BV/TV = bone volume fraction; Tb.N = trabecular number (mm-1); Tb.Th = trabecular thickness (mm); Tb.Sp = trabecular separation (mm); Conn.D = connectivity density (1/mm3
SMI = structure model index; Tb.TMD = Trabecular tissue mineral density (mg of hydroxyappatite/cm^3); Ct.Ar = cortical bone area (mm^2); Tt.Ar = total cross-sectional area inside the periosteal envelope (mm^2); Ct.Ar/Tt.Ar = cortical area fraction;
J = polar moment of inertia (mm^4); Imax/Cmax = cortical moment of inertia around the shorter axis divided by the maximum radius perpendicular to maximum moment of inertia direction (mm3);
Imin/Cmin = cortical moment of inertia around the longer axis divided by the maximum radius perpendicular to minimum moment of inertia direction (mm3); RCR = the response to Ca restriction.
Body size (BS)-corrected line means were used for analysis.

Tb.Th_LowCa	Tb.Th_RCR	Tb.Sp_Basal	Tb.Sp_LowCa	Tb.Sp_RCR	Conn.D_Basal	Conn.D_LowCa	Conn.D_RCR	SMI_Basal	SMI_LowCa	SMI_RCR	Tb.TMD_Basal	Tb.TMD_LowCa	Tb.TMD_RCR
0.5013	-0.2224	-0.5311	-0.4940	0.1446	0.4492	0.4291	0.0617	-0.6324	-0.5404	0.3586	0.1845	0.0137	-0.2406
0.6261	0.1693	-0.4259	-0.4704	-0.1050	0.3435	0.4169	0.2399	-0.4774	-0.5925	-0.0460	0.1653	0.1089	-0.1079
0.2581	0.7184	0.1302	0.0005	-0.4814	-0.1351	0.0382	0.4266	0.1942	-0.2048	-0.8086	-0.0445	0.1052	0.1763
0.5085	-0.1945	-0.4762	-0.4351	0.1580	0.4175	0.3998	0.0693	-0.6241	-0.5184	0.3730	0.1443	-0.0006	-0.2002
0.5738	0.1038	-0.3879	-0.3974	-0.0112	0.3221	0.3510	0.1587	-0.4610	-0.5222	0.0237	0.1037	0.0483	-0.0898
0.1582	0.5690	0.1162	0.0296	-0.4014	-0.1201	-0.0042	0.3509	0.1837	-0.1550	-0.7449	-0.0704	0.0194	0.1144
0.4168	-0.3174	-0.8690	-0.7755	0.2822	0.8446	0.7970	0.1339	-0.9593	-0.7917	0.4897	-0.1875	-0.3596	-0.1515
0.6383	0.1504	-0.7584	-0.7999	-0.0991	0.7259	0.8511	0.4998	-0.8108	-0.9410	0.0257	-0.1720	-0.2088	-0.0024
0.4076	0.8275	0.0559	-0.1468	-0.6380	-0.0813	0.1975	0.7066	0.1322	-0.3662	-0.7943	-0.0197	0.1966	0.2489
0.0641	-0.1108	-0.9899	-0.9240	0.2266	0.9682	0.8957	0.0577	-0.6989	-0.5866	0.3304	-0.5911	-0.5746	0.1616
0.1079	-0.0465	-0.9332	-0.9913	-0.0948	0.8847	0.9330	0.2934	-0.6510	-0.6249	0.1930	-0.5060	-0.5549	0.0655
0.1647	0.2281	0.0967	-0.2391	-0.9336	-0.1629	0.1673	0.7319	0.0642	-0.1923	-0.4396	0.2067	0.0293	-0.2560
0.7158	-0.3979	-0.2518	-0.1934	0.1545	0.2168	0.2284	0.1497	-0.7470	-0.6052	0.4236	0.4738	0.1909	-0.4363
	0.3395	-0.1324	-0.1459	-0.0801	0.0780	0.1844	0.3352	-0.4893	-0.6674	-0.1261	0.4378	0.4806	-0.0550
		0.1253	0.0314	-0.3496	-0.1388	-0.0165	0.2887	0.3186	-0.1083	-0.7932	-0.1013	0.3264	0.5121
			0.9327	-0.2323	-0.9480	-0.8823	-0.0756	0.7401	0.6217	-0.3508	0.5033	0.5250	-0.0972
				0.1187	-0.8594	-0.9076	-0.2934	0.6515	0.6269	-0.1901	0.4572	0.5055	-0.0549
					0.2803	-0.0248	-0.6325	-0.2528	0.0416	0.5680	-0.1408	-0.0803	0.1054
						0.9163	0.0427	-0.7540	-0.6370	0.3232	-0.6210	-0.5886	0.1854
							0.4145	-0.7298	-0.7628	0.1370	-0.5426	-0.5787	0.0861
								-0.1829	-0.5174	-0.4056	0.0786	-0.0850	-0.2090
									0.8405	-0.4590	0.1111	0.2919	0.1808
										0.0200	0.0965	0.1599	0.0513
											0.0162	-0.2431	-0.2955
												0.7100	-0.5700
													0.1733

3);

Ct.Ar_Basal	Ct.Ar_LowCa	Ct.Ar_RCR	Tt.Ar_Basal	Tt.Ar_LowCa	Tt.Ar_RCR	Ct.Ar/Tt.Ar_Basal	Ct.Ar/Tt.Ar_LowCa	Ct.Ar/Tt.Ar_RCR	Ct.Th_Basal	Ct.Th_LowCa	Ct.Th_RCR	*J*_Basal	*J*_LowCa
0.7389	0.6974	-0.0859	0.3796	0.3547	-0.1260	0.4128	0.3602	-0.0285	0.7254	0.6332	-0.1432	0.5192	0.5074
0.6242	0.7405	0.2996	0.2742	0.3071	0.1644	0.4052	0.4588	0.3118	0.6568	0.7280	0.2533	0.4115	0.4934
-0.1359	0.1290	0.8523	-0.1532	-0.0562	0.7165	0.0302	0.2092	0.5981	-0.0540	0.2091	0.7967	-0.1410	0.0085
0.7950	0.7295	-0.1230	0.5203	0.4822	-0.1431	0.2778	0.2242	-0.0632	0.6416	0.5335	-0.1710	0.6441	0.6106
0.7040	0.8042	0.2382	0.4964	0.5352	0.1550	0.1927	0.2224	0.1846	0.5426	0.5674	0.1447	0.6044	0.6771
-0.0964	0.1362	0.8282	-0.0534	0.0432	0.7568	-0.0470	0.0894	0.4389	-0.0797	0.1141	0.6862	-0.0553	0.0767
0.4376	0.3737	-0.1335	0.3209	0.2840	-0.1825	0.0965	0.0618	-0.0341	0.3277	0.2234	-0.1820	0.3843	0.3500
0.3697	0.4648	0.2641	0.2671	0.2832	0.0916	0.0817	0.1602	0.3264	0.2774	0.3452	0.2388	0.3302	0.3882
-0.1115	0.1372	0.7342	-0.1195	-0.0284	0.5607	0.0235	0.1927	0.5621	-0.0486	0.2077	0.7175	-0.1061	0.0391
0.2475	0.2343	-0.0283	0.1641	0.1453	-0.0804	0.0988	0.0959	0.0616	0.2448	0.1751	-0.1135	0.2029	0.2039
0.2146	0.2387	0.0802	0.1525	0.1253	-0.0456	0.0654	0.1255	0.2463	0.2053	0.2107	0.0613	0.1836	0.1944
-0.0534	0.0514	0.4103	-0.0140	-0.0320	0.2269	-0.0641	0.1008	0.4994	-0.0687	0.1223	0.5319	-0.0238	0.0049
0.5188	0.4256	-0.2008	0.3900	0.3373	-0.2522	0.0927	0.0419	-0.1128	0.3246	0.2368	-0.1619	0.4670	0.4077
0.4083	0.5194	0.2560	0.2224	0.2632	0.1028	0.1952	0.2410	0.2369	0.3201	0.4230	0.2925	0.3213	0.3935
-0.1895	0.0703	0.6659	-0.2735	-0.1596	0.5411	0.1584	0.2898	0.4792	-0.0130	0.2340	0.6453	-0.2419	-0.0811
-0.2776	-0.2659	0.0306	-0.1730	-0.1563	0.0889	-0.1195	-0.1163	-0.0680	-0.2736	-0.2057	0.1099	-0.2213	-0.2229
-0.2215	-0.2515	-0.0810	-0.1569	-0.1361	0.0417	-0.0658	-0.1278	-0.2519	-0.2024	-0.2100	-0.0591	-0.1926	-0.2113
0.1918	0.0691	-0.4321	0.0954	0.0920	-0.2887	0.1242	-0.0457	-0.4938	0.2076	-0.0009	-0.5484	0.1270	0.0739
0.2281	0.2145	-0.0398	0.1613	0.1422	-0.0841	0.0786	0.0729	0.0481	0.2184	0.1484	-0.1217	0.1929	0.1919
0.1660	0.2075	0.1318	0.1432	0.1261	0.0027	0.0088	0.0771	0.2622	0.1489	0.1658	0.0926	0.1621	0.1786
-0.0902	0.0254	0.4599	0.0113	0.0212	0.2864	-0.1608	-0.0118	0.4418	-0.1285	0.0272	0.4786	-0.0055	0.0310
-0.4275	-0.3596	0.1309	-0.3761	-0.3313	0.2037	-0.0022	0.0251	0.0372	-0.2471	-0.1517	0.1610	-0.4219	-0.3756
-0.3156	-0.4097	-0.3005	-0.2724	-0.3069	-0.1649	0.0049	-0.0573	-0.2574	-0.1867	-0.2431	-0.2385	-0.3152	-0.3778
0.3286	0.0625	-0.7853	0.3182	0.2125	-0.6676	-0.0410	-0.2093	-0.5558	0.1697	-0.1070	-0.7680	0.3222	0.1648
0.1766	0.1473	-0.0759	-0.0206	-0.0258	-0.0884	0.2373	0.2000	-0.0778	0.2422	0.2407	-0.0159	0.0525	0.0291
0.1452	0.2218	0.0774	-0.0902	-0.0427	0.0296	0.3154	0.3114	0.0506	0.2400	0.3255	0.1523	-0.0089	0.0540
-0.0765	0.0514	0.1893	-0.0719	-0.0087	0.1555	0.0300	0.0751	0.1591	-0.0627	0.0358	0.1925	-0.0806	0.0255
	0.9156	-0.1608	0.7413	0.7091	-0.0947	0.2360	0.1258	-0.2834	0.6859	0.5259	-0.2898	0.8681	0.8314
		0.1810	0.6445	0.6959	0.1607	0.2666	0.2308	-0.0126	0.6732	0.6452	0.0180	0.7781	0.8567
			-0.2090	-0.0453	0.8531	0.0929	0.2606	0.6070	-0.0039	0.2714	0.8374	-0.1900	0.0340
				0.9612	-0.1509	-0.4680	-0.5396	-0.3392	0.0448	-0.1043	-0.3078	0.9703	0.9132
					0.0511	-0.4624	-0.5326	-0.3115	0.0353	-0.0771	-0.2176	0.9324	0.9590
						0.0691	0.1229	0.2327	0.0000	0.1451	0.5707	-0.1340	0.0868
							0.9527	0.0972	0.8416	0.8388	0.0461	-0.2584	-0.2296
								0.3912	0.7563	0.8656	0.2986	-0.3417	-0.2859
									-0.0519	0.3186	0.8619	-0.3216	-0.2052
										0.9141	-0.1201	0.2565	0.2598
											0.2617	0.1006	0.1756
												-0.3047	-0.1412
													0.9467

*J*_RCR	*Imax* _Basal	*Imax* _LowCa	*Imax* _RCR	*Imin* _Basal	*Imin* _LowCa	*Imin*_ RCR
-0.0775	0.5754	0.5470	-0.1078	0.5279	0.4729	-0.1685
0.2609	0.4452	0.5193	0.2284	0.4230	0.4643	0.1851
0.7785	-0.1754	-0.0160	0.7547	-0.1473	0.0134	0.8134
-0.1208	0.6880	0.6324	-0.1544	0.6422	0.5752	-0.1965
0.1990	0.6062	0.6729	0.1808	0.6060	0.6460	0.1371
0.7756	-0.1048	0.0402	0.7560	-0.0668	0.0768	0.8119
-0.1515	0.4133	0.3779	-0.1474	0.3606	0.2865	-0.2092
0.1839	0.3533	0.4297	0.2106	0.3010	0.3202	0.1372
0.6401	-0.1156	0.0603	0.6641	-0.1250	0.0262	0.6702
-0.0279	0.2401	0.2478	-0.0055	0.1918	0.1387	-0.1199
0.0483	0.2278	0.2508	0.0715	0.1763	0.1294	-0.0506
0.3287	-0.0096	0.0343	0.3174	-0.0158	-0.0033	0.3020
-0.2276	0.4857	0.4192	-0.2468	0.4309	0.3487	-0.2565
0.1717	0.3231	0.4101	0.1828	0.2681	0.3298	0.1706
0.5966	-0.2658	-0.0784	0.6223	-0.2665	-0.0886	0.6361
0.0380	-0.2606	-0.2691	0.0144	-0.2025	-0.1561	0.1171
-0.0543	-0.2377	-0.2702	-0.0775	-0.1739	-0.1354	0.0421
-0.3882	0.1250	0.0511	-0.3781	0.1258	0.0912	-0.3542
-0.0367	0.2183	0.2235	-0.0154	0.1950	0.1355	-0.1375
0.0788	0.1907	0.2176	0.1012	0.1622	0.1250	0.0013
0.3351	0.0013	0.0469	0.3332	-0.0285	0.0156	0.3846
0.1752	-0.4276	-0.3854	0.1620	-0.4024	-0.3246	0.2123
-0.2257	-0.3117	-0.3917	-0.2472	-0.2955	-0.3319	-0.2067
-0.7441	0.3488	0.1663	-0.7518	0.3347	0.1652	-0.7592
-0.1055	0.0585	0.0266	-0.1325	0.0364	0.0482	-0.0390
0.0729	-0.0093	0.0667	0.0824	-0.0326	0.0548	0.1126
0.2284	-0.0882	0.0450	0.2776	-0.0860	-0.0007	0.1804
-0.1049	0.8970	0.8523	-0.1184	0.8493	0.8013	-0.1543
0.1922	0.7873	0.8764	0.2109	0.7647	0.8182	0.1148
0.9246	-0.2204	0.0298	0.9413	-0.1859	0.0224	0.8936
-0.1996	0.9258	0.8689	-0.1861	0.9579	0.9095	-0.1942
-0.0099	0.8789	0.9112	0.0107	0.9266	0.9609	-0.0196
0.9413	-0.1529	0.0708	0.9026	-0.1330	0.0994	0.9323
0.1415	-0.1529	-0.1304	0.1136	-0.2676	-0.2742	0.0502
0.2494	-0.2503	-0.1925	0.2357	-0.3484	-0.3359	0.1563
0.4240	-0.3440	-0.2001	0.4710	-0.3138	-0.2327	0.3885
0.0407	0.3420	0.3362	0.0180	0.2678	0.2375	-0.0471
0.2503	0.1653	0.2456	0.2519	0.1196	0.1555	0.1594
0.6977	-0.3471	-0.1546	0.7308	-0.2987	-0.1388	0.7020
-0.1743	0.9768	0.9224	-0.1653	0.9659	0.9183	-0.1806
0.0727	0.9169	0.9782	0.0910	0.9224	0.9653	0.0214
	-0.1774	0.0804	0.9681	-0.1694	0.0526	0.9176
		0.9320	-0.1858	0.9293	0.8687	-0.2039
			0.1057	0.8847	0.9209	0.0047
				-0.1604	0.0708	0.8871
					0.9451	-0.2088
						0.0390

Supplemental Table S7. Heritability Estimates (h^2) for Femoral Bone Traits for Each Dietary Environment and Under Calcium Restriction

Phenotype	Basal	LowCa	RCR
BMD	0.44	0.51	0.31
BMC	0.40	0.46	0.37
BV/TV	0.60	0.54	0.29
Tb.N	0.72	0.72	0.23
Tb.Th	0.49	0.48	0.39
Tb.Sp	0.70	0.70	0.27
Conn.D	0.69	0.62	0.20
SMI	0.55	0.46	0.35
Tb.TMD	0.57	0.46	0.39
Ct.Ar	0.67	0.70	0.41
Tt.Ar	0.75	0.77	0.32
Ct.Ar/Tt.Ar	0.80	0.83	0.32
Ct.Th	0.72	0.72	0.4
J	0.71	0.74	0.36
Imax/Cmax	0.70	0.72	0.39
Imin/Cmin	0.71	0.72	0.36

Abbreviations: BV/TV = bone volume fraction; Tb.N = trabecular number (mm-1);
Tb.Th = trabecular thickness (mm); Tb.Sp = trabecular separation (mm);
Conn.D = connectivity density (1/mm3); SMI = structure model index;
Tb.TMD = Trabecular tissue mineral density (mg of hydroxyappatite/cm^3);
Ct.Th = cortical thickness (mm^2); Ct.Ar = cortical area (mm2), Tt.Ar = total area (mm2);
Ct.Ar/Tt.Ar = cortical area fraction (%); J = polar moment of inertia (mm4);
Imax/Cmax = cortical moment of inertia around the shorter axis divided by the maximum radius perpendicular to maximum moment of inertia direction (mm^3);
Imin/Cmin = cortical moment of inertia around the longer axis divided by the maximum radius perpendicular to minimum moment of inertia direction (mm^3);
RCR = the response to Ca restriction.
Body-size corrected values were used for the analysis

Supplemental Table S8. Summary of Polymorphisms in High-priority Loci of Distal Femur

Loci ID	Chr	[1.5-LOD CI][a] (Mb)	Peak width (Mb)	Total # Polymorphisms[b]	Protein-coding genes	Polymorphism					eQTL/ Cis- eQTL[d]
						Cn/ deleterious[c]	StopL/ StopG	SSD/ SSA	Cf	U5/U3	
F-2B	2	26.56-31.01	4.45	553	149	6/1	0/0	0/0	0	12/16	2/0
F-2C	2	33.04-49.66	16.62	27,810	248	20/2	0/0	0/1	0	11/42	50/2
F-4E	4	81.54-92.00	10.46	20,929	157	57/17	0/1	2/2	1	19/218	4/1
F-8C	8	26.98-49.55	22.57	140,824	342	223/19	0/0	3/1	1	155/756	42/3
F-9A	9	29.82-40.50	10.68	35,447	333	133/39	0/3	4/0	0	51/178	12/3
F-9D	9	112.62-124.12	11.5	16,016	274	58/15	1/0	3/1	2	118/348	121/8
F-15A	15	0.53-12.11	11.58	1,861	131	3/0	0/0	0/0	0	3/15	1/1
F-15C	15	59.80-72.53	12.73	18,111	135	7/1	0/0	0/0	0	0/51	8/0
F-18C	18	29.98-47.08	17.1	30,571	422	257/32	1/3	4/0	0	254/27	15/2
F-20C	X	51.90-69.44	17.54	2,343	262	6/3	0/0	0/0	0	9/9	7/0

Abbreviation: Chr = chromosome; Mp = megabase; Cn = Non-synonymous amino acid substitution; StopL = Polymorphisms cause a lost stop codon; StopG = Polymorphisms cause a premature stop codon; Cf = polymorphisms cause a frameshift; U5 = Polymorphisms in 5'UTR; U3 = Polymorphisms in 3'UTR; eQTL = expression quntitative trait loci.

[a] 1.5-LOD confidence intervals in megabase location (Build GRCm38/mm10)

[b] Polymorphisms between C57Bl/6J and DBA/2J strains

[c] Genes with polymorphisms scored as potentially deleterious nonsynonymous amino acid substitutions

[d] eQTL = Max LOD peak within region and LOD>3.5, cis eQTL = Max LOD is within 2 Mb of the affected gene and LOD>3.5

Supplemental Table S9. Candidate Genes with Predicted Deleterious Amino Acid Change

No.	GENE_NAME	Chr	QTL ID	bp38	TYPE	B6 allele	DBA allele	SNP ID	Amino Acid Ref	Amino Acid Alt	Length	Strand	Position	PROVEAN SCORE	PROVEAN Prediction (cutoff=-2.5)	PROVEAN #SEQ	PROVEAN #CLUSTER	SIFT SCORE	SIFT Prediction (cutoff=0.05)	SIFT MEDIAN_INFO	SIFT #SEQ	Lu et al. (2019)[a] Bone Ignorome[a]	Lu et al. (2019)[a] N Hits in Bone GO	Lu et al. (2019)[a] mRNA level in Farber	Lu et al. (2019)[a] Morris 2019 GWAS Hits	Lu et al. (2019)[a] Any Bone	BioGPS mRNA[b] Osteo-blast	BioGPS mRNA[b] Osteo-clast	Alias
1	Egfl7	2	2B	26589371	Single AA Change	C	T	rs50949904	S	L	49	1	45	-3.34	Deleterious	34	5	1	Tolerated	3.95	18	N	0	7.36	no	0	1x	1x	
2	Lrp1b	2	2C	41408958	Single AA Change	G	A	rs27143708	T	I	4630	-1	1002	-3.52	Deleterious	141	30	0.016	Damaging	2.91	69	N	0	7.40	no	0	1x	1x	
3	Lrp1b	2		41502142	Single AA Change	A	C	rs33596323	V	G	4630	-1	501	-4.49	Deleterious	141	30	0.063	Tolerated	2.91	68	N	0	7.40	no	0			
4	1700019E08Rik	2		45697028	SSA	G	A	rs50560661												SSA		N	0	7.27	no	0	NA	NA	
5	Gm5860	4	4E	82066042	SSD	T	C	rs28102779														#N/A	#N/A	#N/A	#N/A	#N/A	NA	NA	
6	Zdhhc21	4		82820444	Single AA Change	A	G	rs28090517	I	T	133		123	-3.99	Deleterious	81	30	0.002	Damaging	3.11	81	N	0	8.70	no	0	0.3X	1.5X	
7	Ccdc171	4		83799190	Cf	0	1	rs241427147														N	0	6.99	no	0	1X	0.8X	
8	Gm12414	4		84125995	SSA	C	T	rs259891202														#N/A	#N/A	#N/A	#N/A	#N/A	NA	NA	
9	LOC105247162	4		85398157	SSA	A	G	rs48391852														#N/A	#N/A	#N/A	#N/A	#N/A	NA	NA	
10	LOC105247163	4		86711823	SSD	G	T	rs49500046														#N/A	#N/A	#N/A	#N/A	#N/A	NA	NA	
11	Dennd4c	4		86825451	Single AA Change	A	C	rs28079697	N	H	1955		1226	-1.05	Neutral	168	30	0.046	Damaging	3.41	92	N	0	7.39	no	0	0.6X	3X	
12	Dennd4c	4		86825451	Single AA Change	A	C	rs28079697	N	H	1906		1177	-1.22	Neutral	168	30	0.045	Damaging	3.4	91	N	0	7.39	no	0			
13	Focad	4		88169948	Single AA Change	C	A	rs28082224	P	H	1798		145	-3	Deleterious	82	30	0	Damaging	3.11	42	N	0	9.86	no	0	1.5X	1X	
14	Focad	4		88169948	Single AA Change	C	A	rs28082224	P	H	1712		145	-3.01	Deleterious	86	30	0.001	Damaging	3.05	10	N	0	9.86	no	0			
15	Ifnb1	4		88522260	Single AA Change	A	G	rs28084067	I	T	182		172	-1.35	Neutral	183	30	0.004	Damaging	2.82	68	N	0	7.25	no	0	1X	1X	
16	Ifnab	4		88690833	Single AA Change	G	A	rs13477830	S	F	190		132	-2.61	Deleterious	577	30	0.035	Damaging	3.13	395	N	0	7.09	no	0	1X	1X	
17	Gm16686	4		88755298	Single AA Change	T	G	rs236049359	H	P	131		98	-10	Deleterious	1	1	NA	NA	NA	NA	#N/A	#N/A	#N/A	#N/A	#N/A	NA	NA	
18	Ifna7	4		88816262	Single AA Change	T	C	rs236000404	V	A	190		12	-2.69	Deleterious	578	30	0.063	Tolerated	3.08	321	N	0	7.26	no	0	NA	NA	
19	Ifna7	4		88816418	Single AA Change	A	C	rs48960566	E	A	190		64	-4.7	Deleterious	578	30	0.007	Damaging	3.08	399	N	0	7.26	no	0			
20	Ifna7	4		88816549	Single AA Change	T	G	rs230089376	F	V	190		108	-4.44	Deleterious	578	30	0.013	Damaging	3.08	399	N	0	7.26	no	0			
21	Ifna11	4		88820172	Single AA Change	C	A	rs46952217	Q	K	190		72	-3.57	Deleterious	580	30	0.02	Damaging	3.1	399	N	0	7.32	no	0	NA	NA	
22	Ifna11	4		88820373	Single AA Change	T	G	rs50246521	S	A	190		139	-2.26	Neutral	580	30	0.049	Damaging	3.08	383	N	0	7.32	no	0			
23	Ifna11	4		88820405	Single AA Change	G	C	rs47225128	R	S	190		149	-4.17	Deleterious	580	30	0.027	Damaging	3.1	399	N	0	7.32	no	0			
24	Ifna6	4		88827603	Nonsense	T	A	rs584563474	L	*	189		63	NA	NA	582	30	NA	NA	NA	NA	N	0	7.26	no	0	NA	NA	
25	Ifna6	4		88827603	StopG	T	A	rs584563474	L	*			63									N	0	7.26	no	0			
26	Ifna6	4		88827615	Single AA Change	A	G	rs48813584	D	G	189		67	-3.34	Deleterious	582	30	0.339	Tolerated	3.03	374	N	0	7.26	no	0			
27	Mtap	4		89176914	Single AA Change	C	T	rs28072968	R	C	283		268	-2.78	Deleterious	119	30	0.089	Tolerated	2.93	116	N	0	7.88	no	0	5X (5 d)	1.5X	
28	Rab11fip1	8	8C	27174563	Single AA Change	C	G	rs33471772	G	A	645	-1	8	-1.07	Neutral	105	30	0.013	Damaging	3.83	30	N	0	7.08	no	0	1x	1x	
29	Rab11fip1	8		27174563	Single AA Change	C	G	rs33471772	G	A	1166	-1	8	-1.07	Neutral	105	30	0.009	Damaging	3.52	36	N	0	7.08	no	0			
30	Tex24	8		27345047	Single AA Change	A	G	rs49931398	H	R	351	1	201	-3.67	Deleterious	3	3	NA	NA	NA	NA	#N/A	#N/A	#N/A	#N/A	#N/A	1x	1x	
31	Tti2	8		31151445	Single AA Change	A	G	rs33377565	D	G	512	1	199	-1.67	Neutral	66	30	0.036	Damaging	2.9	42	#N/A	#N/A	#N/A	#N/A	#N/A	< 1x	< 1x	
32	7420700N18Rik	8		31378965	SSD	G	T	rs46504408												SSD		#N/A	#N/A	#N/A	#N/A	#N/A	> 1x (d5)	1x	
33	LOC102641740	8		31624377	SSD	C	T	rs49993050												SSD		#N/A	#N/A	#N/A	#N/A	#N/A	NA	NA	
34	Nrg1	8		31818096	Single AA Change	A	T	rs32629018	S	T	700	-1	679	-1.2	Neutral	317	30	0.002	Damaging	3.41	115	N	0	7.05	no	0	1x	> 1x	
35	Nrg1	8		31915922	SSA	T	C	rs33179604												SSA		N	0	7.05	no	0	1x	1x	
36	Wrn	8		33241108	Single AA Change	T	G	rs46514474	L	F	1401	-1	1308	-1.14	Neutral	67	30	0.002	Damaging	2.94	53	N	0	10.39	no	0			
37	Wrn	8		33241108	Single AA Change	T	G	rs46514474	L	F	1401	-1	1308	-1.14	Neutral	67	30	0.002	Damaging	2.94	53	N	0	10.39	no	0			
38	Wrn	8		33255535	Single AA Change	T	C	rs48550002	E	G	1401	-1	1182	-3.64	Deleterious	67	30	0.111	Tolerated	2.88	59	N	0	10.39	no	0			
39	Wrn	8		33255535	Single AA Change	T	C	rs48550002	E	G	1401	-1	1182	-3.64	Deleterious	67	30	0.111	Tolerated	2.88	59	N	0	10.39	no	0			
40	Tex15	8		33546343	Single AA Change	C	G	rs30228602	A	G	148	1	99	-2.05	Neutral	74	30	0.02	Damaging	2.77	68	N	0	6.94	no	0	13x (d5)	1x	
41	Tex15	8		33546343	Single AA Change	C	G	rs30228602	A	G	1279	1	99	-1.66	Neutral	90	30	0.035	Damaging	3.16	82	N	0	6.94	no	0			
42	Tex15	8		33574189	Single AA Change	A	G	rs4227113	S	G	2785	1	1216	-2.26	Neutral	35	17	0.007	Damaging	3.39	27	N	0	6.94	no	0			
43	Tex15	8		33582192	Single AA Change	C	T	rs3703388	P	L	2785	1	2589	-2.99	Deleterious	35	17	0.89	Tolerated	3.39	26	N	0	6.94	no	0			
44	Rbpms	8		33795103	Single AA Change	G	T	rs13472591	P	T	191	-1	149	-0.64	Neutral	140	30	0.001	Damaging	3.61	47	N	0	9.93	no	0	2x	< 1x	
45	Dctn6	8		34098917	SSD	A	G	rs33524403												SSD		N	0	7.05	no	0	> 1x	1x	AU044699, WS-3, p27
46	Gm34705	8		35352244	SSA	C	T	rs30338405												SSA		#N/A	#N/A	#N/A	#N/A	#N/A	NA	NA	
47	Dlc1	8		36938452	Single AA Change	A	G	rs30480037	L	P	1543	-1	61	-1.04	Neutral	152	30	0.05	Damaging	3.98	27	Y	0	7.80	yes	1	(d5), 6x (d1	< 1x	
48	Msr1	8		39623964	Single AA Change	T	G	rs30713937	N	H	458	-1	202	-2	Neutral	120	30	0.036	Damaging	2.83	73	N	0	7.14	no	0	<1x	222x	CD204, SCARA1
49	Msr1	8		39623964	Single AA Change	T	G	rs30713937	N	H	354	-1	202	-1.22	Neutral	135	30	0.045	Damaging	2.8	69	N	0	7.14	no	0			
50	Zdhhc2	8		40472999	Single AA Change	G	T	rs39259851	S	I	366	1	345	-2.51	Deleterious	152	30	0.087	Tolerated	3.01	139	N	0	7.45	no	0	3x (d5)	1x	
51	Adam24	8		40680039	Single AA Change	C	A	rs33077682	A	E	761	1	182	-3.7	Deleterious	120	30	0.066	Tolerated	2.78	100	N	0	7.00	no	0	1x	1x	
52	Adam24	8		40680855	Single AA Change	G	A	rs13479720	R	H	761	1	454	-1.14	Neutral	120	30	0.05	Damaging	2.81	91	N	0	7.00	no	0			
53	Adam24	8		40681301	Single AA Change	G	A	rs33441119	A	T	761	1	603	-0.15	Neutral	120	30	0.048	Damaging	2.78	100	N	0	7.00	no	0			
54	Adam39	8		40825123	Cf	T	C	rs864312114				NA								Cf		N	0	7.40	no	0			Adam20
55	Slc7a2	8		40881684	SSD	G	A	rs33219431												SSD		N	0	7.11	no	0	[illegible]0x (d14,d21	1x	AI158848, Atrc2, Cat2, Tea
56	Mtus1	8		41083891	Single AA Change	C	T	rs48828775	G	R	759	-1	263	-5.18	Deleterious	54	14	0.044	Damaging	3.42	44	Y	0	7.30	no	1	1x	< 1x	
57	Mtus1	8		41083891	Single AA Change	C	T	rs48828775	G	R	1210	-1	263	-1.96	Neutral	119	30	0.037	Damaging	3.78	44	Y	0	7.30	no	1			
58	Mtus1	8		41083891	Single AA Change	C	T	rs48828775	G	R	1210	-1	263	-1.96	Neutral	119	30	0.037	Damaging	3.78	44	Y	0	7.30	no	1			
59	Mtus1	8		41084040	Single AA Change	T	C	rs51455351	Y	C	759	-1	213	-2.9	Deleterious	54	14	0.162	Tolerated	3.42	43	Y	0	7.30	no	1			
60	Mtus1	8		41084238	Single AA Change	C	G	rs51126891	C	S	759	-1	147	-5.15	Deleterious	54	14	0.131	Tolerated	3.42	42	Y	0	7.30	no	1			
61	Mtus1	8		41084260	Single AA Change	T	C	rs48575407	R	G	759	-1	140	-3.35	Deleterious	54	14	0.039	Damaging	3.42	42	Y	0	7.30	no	1			
62	Mtus1	8		41084260	Single AA Change	T	C	rs48575407	R	G	1210	-1	140	-1.14	Neutral	119	30	0.036	Damaging	3.78	42	Y	0	7.30	no	1			
63	Mtus1	8		41084260	Single AA Change	T	C	rs48575407	R	G	1210	-1	140	-1.14	Neutral	119	30	0.036	Damaging	3.78	42	Y	0	7.30	no	1			
64	Mtus1	8		41084580	Single AA Change	A	G	rs32726714	L	P	38	-1	33	-3.19	Deleterious	11	4	NA	NA	NA	NA	Y	0	7.30	no	1			
65	Gm16348	8		41219350	SSD	G	A	rs225216238												SSD		#N/A	#N/A	#N/A	#N/A	#N/A	NA	NA	
66	Prdm10	9	9A	31316154	Nonsense	G	A	rs237188889	W	*	1135	1	14	NA	NA	187	30	NA	NA	NA	NA	#N/A	#N/A	#N/A	#N/A	#N/A	< 1x	< 2x	
67	Srpr	9		35212457	Single AA Change	T	C	rs30437276	L	S	636	1	104	-0.79	Neutral	117	30	0.047	Damaging	3.01	90	N	0	10.04	no	0	2x	1x	
68	Gm5916	9		36120740	Single AA Change	C	T	rs46247482	A	T	102	-1	83	-2	Neutral	7	6	0.04	Damaging	3.73	7	#N/A	#N/A	#N/A	#N/A	#N/A	NA	NA	
69	Gm5916	9		36121054	Single AA Change	C	G	rs33698885	C	S	102	-1	28	-10	Deleterious	7	6	0	Damaging	3.73	7	#N/A	#N/A	#N/A	#N/A	#N/A	NA	NA	
70	Gm17689	9		36581303	Single AA Change	G	A	rs30187173	S	F	109	-1	102	-3.02	Deleterious	37	22	0.019	Damaging	3.05	13	#N/A	#N/A	#N/A	#N/A	#N/A	NA	NA	
71	Tbrg1	9		37649489	Single AA Change	G	T	rs49592959	P	T	406	-1	364	-1.63	Neutral	92	30	0.01	Damaging	3.67	42	N	0	10.24	no	0	4x	1.5x	
72	Olfr877	9		37855586	Single AA Change	C	A	rs250927516	A	D	311	1	256	-5.07	Deleterious	616	30	0	Damaging	3.37	399	N	0	7.05	no	0	NA	NA	
73	Olfr884	9		38047269	StopG	G	T	rs30277381	G	*			16									N	0	7.43	no	0			
74	Olfr887	9		38085007	Single AA Change	C	G	rs256459975	T	R	309	1	57	-5.05	Deleterious	641	30	0	Damaging	3.34	399	N	0	7.27	no	0			
75	Olfr893	9		38209416	Single AA Change	C	T	rs49614089	A	V	312	1	121	-3.58	Deleterious	572	30	0.006	Damaging	3.35	399	N	0	7.21	no	0	NA	NA	
76	Olfr893	9		38209572	Single AA Change	C	T	rs32537926	S	L	312	1	173	-3.26	Deleterious	572	30	0.048	Damaging	3.34	398	N	0	7.21	no	0			
77	Olfr143	9		38253430	Single AA Change	G	A	rs47694530	M	I	313	1	4	-3.15	Deleterious	572	30	0.025	Damaging	3.36	360	N	0	7.13	no	0	NA	NA	
78	Olfr898	9		38349350	Single AA Change	T	C	rs46935878	M	T	316	1	89	-3.98	Deleterious	572	30	0.07	Tolerated	3.31	399	N	0	7.67	no	0	NA	NA	
79	Olfr250	9		38367683	Single AA Change	A	T	rs49575689	T	S	331	1	46	-1.46	Neutral	570	30	0.049	Damaging	3.3	398	N	0	6.96	no	0	NA	NA	
80	Olfr250	9		38367804	Single AA Change	T	A	rs51519640	V	D	331	1	86	-5.48	Deleterious	570	30	0.019	Damaging	3.3	397	N	0	6.96	no	0	NA	NA	
81	Olfr250	9		38368255	Single AA Change	C	G	rs32549630	I	M	331	1	236	-0.24	Neutral	570	30	0.034	Damaging	3.3	398	N	0	6.96	no	0			
82	Olfr251	9		38378291	Single AA Change	A	C	rs47808718	K	Q	316	1	137	0.1	Neutral	572	30	0.025	Damaging	3.35	398	N	0	7.02	no	0	NA	NA	
83	Olfr251	9		38378401	Single AA Change	T	G	rs32554625	S	R	316	1	173	-1.74	Neutral	572	30	0.049	Damaging	3.35	397	N	0	7.02	no	0			

84	Olfr902	9		38449105	Single AA Change	A	G	rs49837149	T	A	309	1	78	-3.72	Deleterious	568	30	0.01	Damaging	3.38	397	N	0	7.13	no	0	NA	NA	
85	Olfr902	9		38449187	Single AA Change	G	T	rs50827544	C	F	309	1	105	-6.41	Deleterious	568	30	0.036	Damaging	3.38	399	N	0	7.13	no	0			
86	Olfr902	9		38449261	Single AA Change	C	T	rs47025576	L	F	309	1	130	-3.8	Deleterious	568	30	0.008	Damaging	3.38	399	N	0	7.13	no	0			
87	Olfr902	9		38449507	Single AA Change	A	G	rs32553262	T	A	309	1	212	-3.71	Deleterious	568	30	0.025	Damaging	3.38	399	N	0	7.13	no	0			
88	Olfr902	9		38449633	Single AA Change	G	A	rs46759916	G	R	309	1	254	-4.83	Deleterious	568	30	0.016	Damaging	3.38	399	N	0	7.13	no	0			
89	Olfr905	9		38473301	Single AA Change	C	A	rs29742813	L	I	310	1	185	-1.78	Neutral	567	30	0.036	Damaging	3.37	399	N	0	7.09	no	0	NA	NA	
90	Olfr906	9		38488962	Single AA Change	G	T	rs29686032	W	L	311	1	311	0.06	Neutral	563	30	0.021	Damaging	3.55	24	N	0	6.93	no	0	NA	NA	
91	Olfr910	9		38539266	Single AA Change	G	A	rs48264518	V	M	310	1	124	-2.54	Deleterious	598	30	0.03	Damaging	3.36	399	N	0	6.96	no	0	NA	NA	
92	Olfr915	9		38647307	StopG	C	T	rs50479609	W	*			72									N	0	7.16	no	0			
93	Olfr917	9		38665346	Single AA Change	G	T	rs30024215	T	N	309	-1	166	-3.31	Deleterious	586	30	0.019	Damaging	3.38	399	#N/A	#N/A	#N/A	#N/A	#N/A	NA	NA	
94	Olfr923	9		38828408	Single AA Change	G	A	rs13480148	G	D	317	1	239	-6.63	Deleterious	613	30	0.004	Damaging	3.34	398	N	0	7.07	no	0	NA	NA	
95	Olfr936	9		39046745	Single AA Change	C	T	rs29892095	D	N	267	-1	225	-3.81	Deleterious	400	30	0.103	Tolerated	3.45	398	#N/A	#N/A	#N/A	#N/A	#N/A	NA	NA	
96	Olfr938	9		39077959	Single AA Change	G	A	rs13480149	P	L	315	-1	262	-9.65	Deleterious	316	30	0.001	Damaging	3.33	399	N	0	7.06	no	0	NA	NA	
97	Olfr27	9		39144293	Single AA Change	C	G	rs251014158	S	R	311	1	64	-3.45	Deleterious	408	30	0.063	Tolerated	3.37	397	N	0	6.99	no	0	NA	NA	
98	Olfr27	9		39144543	Single AA Change	A	T	rs578863519	I	L	311	1	148	-1.89	Neutral	408	30	0.007	Damaging	3.37	397	N	0	6.99	no	0	NA	NA	
99	Olfr27	9		39144544	Single AA Change	T	C	rs582470086	I	T	311	1	148	-2.68	Deleterious	408	30	0.025	Damaging	3.37	397	N	0	6.99	no	0	NA	NA	
100	Olfr27	9		39144609	Single AA Change	A	C	rs46919168	N	H	311	1	170	-1.01	Neutral	408	30	0.007	Damaging	3.37	397	N	0	6.99	no	0			
101	Olfr27	9		39144912	Single AA Change	G	A	rs49125436	A	T	311	1	271	-1.73	Neutral	408	30	0.004	Damaging	3.38	396	N	0	6.99	no	0			
102	Olfr27	9		39144913	Single AA Change	C	T	rs48656703	A	V	311	1	271	-2.67	Deleterious	408	30	0.011	Damaging	3.38	396	N	0	6.99	no	0	NA	NA	
103	Olfr1537	9		39238362	Single AA Change	G	A	rs29687632	P	S	314	-1	24	-5.9	Deleterious	397	30	0.087	Tolerated	3.34	392	#N/A	#N/A	#N/A	#N/A	#N/A	NA	NA	
104	Olfr44	9		39485248	Single AA Change	T	C	rs30182798	N	D	314	-1	2	-0.25	Neutral	330	30	0.047	Damaging	3.53	100	N	0	7.17	no	0	NA	NA	
105	Olfr957	9		39511015	Single AA Change	G	A	rs30138551	S	F	311	-1	235	-3.69	Deleterious	409	30	0.124	Tolerated	3.37	398	N	0	7.01	no	0	NA	NA	
106	Olfr957	9		39511562	Single AA Change	A	G	rs30229886	S	P	311	-1	53	-3.74	Deleterious	409	30	0.062	Tolerated	3.37	397	N	0	7.01	no	0			
137	Ttc21a	9	9D	119956886	Single AA Change	C	T	rs13480451	P	L	1314	1	650	-7.11	Deleterious	125	30	0.003	Damaging	2.85	112	N	0	7.39	no	0	1X	1X	
138	Xirp1	9		120017222	Single AA Change	G	A	rs51241200	T	M	1132	-1	865	0.18	Neutral	88	30	0.042	Damaging	3.79	36	N	0	9.16	no	0	1X	1X	
139	Cx3cr1	9		120051864	SSA	C	T	rs29745148																			0.3X	8X	
140	Gm34159	9		120357612	SSD	A	C	rs46139020																			NA	NA	
141	Rpl14	9		120574285	Single AA Change	C	T	rs30384543	A	V	217	1	182	-0.43	Neutral	131	30	0.003	Damaging	3.54	68	N	0	7.50	no	0	1X	1X	
142	Ulk4	9		121257392	Single AA Change	G	A	rs46953710	P	L	139	-1	6	-1.95	Neutral	56	15	0.029	Damaging	3.47	48	N	0	7.45	no	0	1X	1X	
143	Gm35095	9		121702569	StopL	A	C	rs47015240	*	C			328														NA	NA	
144	Sec22c	9		121705212	SSD	A	G	rs47410558																			0.7X	0.5X	
145	Nktr	9		121742594	Single AA Change	T	C	rs52463622	C	R	1453	1	194	1.11	Neutral	127	30	0.021	Damaging	3.72	71	N	0	7.45	no	0	1X	1X	
146	Nktr	9		121750377	Single AA Change	A	T	rs30370786	Q	H	1453	1	1170	-0.57	Neutral	127	30	0	Damaging	3.88	33	N	0	7.45	no	0	NA	NA	
147	Nktr	9		121750573	Single AA Change	G	C	rs30274440	A	P	1453	1	1236	-0.36	Neutral	127	30	0.047	Damaging	3.89	33	N	0	7.45	no	0	NA	NA	
148	Snrk	9		122166891	Single AA Change	G	C	rs33499127	D	H	748	1	579	-0.5	Neutral	110	30	0.006	Damaging	3.83	40	N	0	7.39	no	0	0.5X	0.3X	
149	Snrk	9		122166891	Single AA Change	G	C	rs33499127	D	H	748	1	579	-0.5	Neutral	110	30	0.006	Damaging	3.83	40	N	0	7.39	no	0	NA	NA	
150	Topaz1	9		122749236	Single AA Change	C	T	rs30089094	R	C	1653	1	404	-1.17	Neutral	40	16	0.025	Damaging	3.5	32	#N/A	#N/A	#N/A	#N/A	#N/A	NA	NA	
151	Topaz1	9		122788868	Single AA Change	G	C	rs33520681	L	F	1653	1	1243	-2.04	Neutral	40	16	0.01	Damaging	3.31	34	#N/A	#N/A	#N/A	#N/A	#N/A	NA	NA	
152	Zkscan7	9		122888918	Single AA Change	A	G	rs33528847	D	G	718	1	126	-4.75	Deleterious	217	30	0.007	Damaging	2.86	47	#N/A	#N/A	#N/A	#N/A	#N/A	NA	NA	
153	Zkscan7	9		122896033	Single AA Change	G	A	rs49800985	G	E	718	1	689	-5.48	Deleterious	217	30	0.005	Damaging	2.85	60	#N/A	#N/A	#N/A	#N/A	#N/A	NA	NA	
154	Zkscan7	9		122896086	Single AA Change	G	A	rs51855007	V	I	718	1	707	-0.73	Neutral	217	30	0.023	Damaging	2.86	59	#N/A	#N/A	#N/A	#N/A	#N/A	NA	NA	
155	Ccr1	9		123964047	Single AA Change	A	G	rs8254392	F	L	355	-1	149	-4.3	Deleterious	512	30	0.03	Damaging	3.17	398	N	10	7.44	yes	1	0.2X	500X	
156	Ccr2	9		124102018	SSD	0	1	rs265541228																			0.2X	0.5X	
166	Fam135b	15	15C	71463885	Single AA Change	T	G	rs50815982	T	P	1403	-1	487	0.2	Neutral	157	30	0.004	Damaging	3.7	43	#N/A	#N/A	#N/A	#N/A	#N/A	1x	1x	
184	Bin1	18	18C	32430835	Single AA Change	C	T	rs30009182	T	I	588	1	451	-1.69	Neutral	177	30	0.015	Damaging	3.36	94	N	0	7.65	no	0	1x	1x	
185	Nrep	18		33463544	SSD	G	A	rs251302461												SSD		N	0	11.89	no	0	27x (d21)	1x	AI325076, D0H4S114, Harp, P311, PTZ17, SEZ17
186	LOC105246394	18		34182863	SSD	T	G	rs51108565												SSD		#N/A	#N/A	#N/A	#N/A	#N/A	NA	NA	
187	Gm10548	18		34221481	SSD	A	G	rs47085716												SSD		#N/A	#N/A	#N/A	#N/A	#N/A	NA	NA	
188	Apc	18		34315736	Single AA Change	G	T	rs51271694	R	L	2842	1	1895	-0.9	Neutral	105	30	0.018	Damaging	3.41	62	N	5	6.83	yes	1	2x	> 1x	
189	Pcdha2	18		36932476	Single AA Change	G	C	rs24884904	G	A	948	1	731	-2.5	Deleterious	357	30	0.273	Tolerated	3.14	380	N	0	7.19	no	0	1x	1x	
190	Pcdhb2	18		37295399	Single AA Change	T	A	rs29965662	L	M	799	1	142	-1.75	Neutral	404	30	0.004	Damaging	3.32	394	N	0	7.28	no	0	1.75x	1x	
191	Pcdhb2	18		37295969	Single AA Change	A	G	rs29578654	S	G	799	1	332	-1.87	Neutral	404	30	0.004	Damaging	3.32	396	N	0	7.28	no	0			
192	Pcdhb2	18		37296063	Single AA Change	C	T	rs30255535	P	L	799	1	363	-7.42	Deleterious	404	30	0.034	Damaging	3.32	397	N	0	7.28	no	0	2x	1x	
193	Pcdhb3	18		37301419	Single AA Change	C	T	rs13499243	S	F	784	1	146	-3.99	Deleterious	400	30	0.012	Damaging	3.34	396	N	0	7.54	no	0	7.5x	1x	
194	Pcdhb3	18		37302352	Single AA Change	C	T	rs13490257	T	I	784	1	457	-5.53	Deleterious	400	30	0.029	Damaging	3.34	399	N	0	7.54	no	0			
195	Pcdhb3	18		37302457	Single AA Change	C	T	rs51590120	P	L	784	1	492	-8.06	Deleterious	400	30	0.041	Damaging	3.34	398	N	0	7.54	no	0			
196	Pcdhb3	18		37302690	Single AA Change	G	A	rs50815504	A	T	784	1	570	-3.18	Deleterious	400	30	0.02	Damaging	3.34	395	N	0	7.54	no	0			
197	Pcdhb4	18		37308800	Single AA Change	C	T	rs29724294	L	F	784	1	388	-3.59	Deleterious	388	30	0.001	Damaging	3.37	394	N	0	7.29	no	0	1x	1x	
198	Pcdhb4	18		37309062	Single AA Change	C	T	rs30109710	T	I	784	1	475	-5.78	Deleterious	388	30	0.01	Damaging	3.37	395	N	0	7.29	no	0			
199	Pcdhb4	18		37309110	Single AA Change	C	T	rs29582436	P	L	784	1	491	-8.64	Deleterious	388	30	0.041	Damaging	3.37	394	N	0	7.29	no	0			
200	Pcdhb4	18		37309112	Single AA Change	C	T	rs29973731	P	S	784	1	492	-5	Deleterious	388	30	0.093	Tolerated	3.37	394	N	0	7.29	no	0			
201	Pcdhb4	18		37309263	Single AA Change	G	A	rs29543274	S	N	784	1	542	-2.93	Deleterious	388	30	0.011	Damaging	3.36	391	N	0	7.29	no	0			
202	Pcdhb4	18		37309817	Single AA Change	G	T	rs48799425	V	L	784	1	727	-2.46	Neutral	388	30	0.025	Damaging	3.37	386	N	0	7.29	no	0			
203	Pcdhb5	18		37320683	Single AA Change	G	A	rs13490341	E	K	792	1	39	-3.15	Deleterious	392	30	0.01	Damaging	3.33	386	N	0	6.91	no	0	5x (d14)	1x	
204	Pcdhb5	18		37321124	Single AA Change	G	A	rs261373744	E	K	792	1	186	-2.91	Deleterious	392	30	0.073	Tolerated	3.34	398	N	0	6.91	no	0			
205	Pcdhb5	18		37321227	Single AA Change	C	T	rs50182297	P	L	792	1	220	-8.81	Deleterious	392	30	0.005	Damaging	3.34	398	N	0	6.91	no	0			
206	Pcdhb5	18		37321688	Single AA Change	T	G	rs51763414	S	A	792	1	374	-2.61	Deleterious	392	30	0.037	Damaging	3.34	398	N	0	6.91	no	0			
207	Pcdhb5	18		37322196	Single AA Change	A	G	rs45916807	Q	R	792	1	543	-3.02	Deleterious	392	30	0.013	Damaging	3.34	397	N	0	6.91	no	0			
208	Pcdhb5	18		37322738	Single AA Change	G	A	rs48202300	G	S	792	1	724	-4.71	Deleterious	392	30	0.055	Tolerated	3.33	394	N	0	6.91	no	0			
209	Pcdhb6	18		37334643	Single AA Change	C	T	rs48561161	P	S	772	1	206	-2.71	Deleterious	388	30	0.202	Tolerated	3.37	395	N	0	7.06	no	0	1x	1x	
210	Pcdhb6	18		37334769	Single AA Change	T	G	rs45661470	S	A	772	1	248	-0.87	Neutral	388	30	0.024	Damaging	3.37	395	N	0	7.06	no	0			
211	Pcdhb7	18		37342392	Single AA Change	G	A	rs29828534	G	S	829	1	194	-5.85	Deleterious	388	30	0.001	Damaging	3.32	395	N	0	6.86	no	0	10x (d21)	1x	
212	Pcdhb7	18		37343607	Single AA Change	G	A	rs29672858	A	T	829	1	599	-2.32	Neutral	388	30	0.011	Damaging	3.32	391	N	0	6.86	no	0			
213	Pcdhb7	18		37343812	Single AA Change	C	T	rs29920152	P	L	829	1	667	-3.08	Deleterious	388	30	0.131	Tolerated	3.32	393	N	0	6.86	no	0			
214	Pcdhga9	18		37674553	Single AA Change	C	G	rs31769244	L	V	928	1	20	-1.99	Neutral	335	30	0.026	Damaging	3.04	261	N	0	7.25	no	0	8x	1x	
215	Pcdhga9	18		37674668	Single AA Change	A	T	rs47377560	E	V	928	1	58	-5.61	Deleterious	335	30	0.014	Damaging	3.01	393	N	0	7.25	no	0			
216	Pcdhga9	18		37675550	Single AA Change	C	T	rs31767868	S	L	928	1	352	-4.24	Deleterious	335	30	0.269	Tolerated	3.01	399	N	0	7.25	no	0			
217	Pcdhga9	18		37676071	Single AA Change	C	A	rs50204375	Q	K	928	1	526	-2.64	Deleterious	335	30	0.102	Tolerated	3.01	399	N	0	7.25	no	0			
218	Pcdhga9	18		37743855	Nonsense	A	T	rs47917604	K	*	930	1	539	NA	NA	240	30	NA	NA	NA	NA	N	0	7.25	no	0			
219	Pcdhgb6	18		37743855	StopG	A	T	rs47917604	K	*			539							StopG		N	0	7.42	no	0	8x (d21)	1x	
220	Pcdhgb8	18		37763496	StopG	A	T	rs46608188	K	*			540							StopG		N	0	7.23	no	0	8x	1x	
221	Pcdhgb8	18		37764110	StopG	A	C	rs49046833	Y	*			744							StopG		N	0	7.23	no	0			
222	Mcc	18		44574601	SSD	C	T	rs30032146												SSD		N	0	8.29	no	0	> 1x	1x	D18Ertd451e, E330037C19
223	Zfp36l3	X	20C	53775441	Single AA Change	T	C	rs13483759	M	V	725	-1	269	-0.2	Neutral	101	30	0.014	Damaging	4.32	3	N	0	7.05	no	0	NA	NA	
224	Zfp36l3	X		53775441	Single AA Change	T	C	rs13483759	M	V	725	-1	269	-0.2	Neutral	101	30	0.014	Damaging	4.32	3	N	0	7.05	no	0			

225	Vgll1	X	57101675	Single AA Change	T	G	rs48289336	I	M	307	1	126	0.76	Neutral	107	30	0.036	Damaging	3.44	27	N	0	6.93	no	0	1x	1x
226	Vgll1	X	57101675	Single AA Change	T	G	rs48289336	I	M	150	1	22	1.36	Neutral	29	11	0.04	Damaging	3.34	27	N	0	6.93	no	0		
227	Vgll1	X	57101932	Single AA Change	A	G	rs29060172	H	R	150	1	108	-2.85	Deleterious	29	11	0.284	Tolerated	3.49	11	N	0	6.93	no	0		

[a] Bone Ignorome and GO analysis were reported in Lu et al. (2019) JBMR Plus doi/10.1002/jbm4.10241

[b] Relative to the Mean across all tissues

Supplemental Table S10. *Cis* eQTL under the High Priority Loci of Femur BXD QTL

QTL	Locus	Traits	Diet	Parental influence	Correlation 1	Correlation 2	Correlation 3	Symbol	Gene Chr	Gene Location	Mean (log2)	Max LOD	Max LOD Location	Distance (Mb)	Additive Effect (DBA+)	Bone Ignorome	# Bone GO Terms	mRNA Level. Farber	Morris 2019 GWAS	Any bone	BioGPS (M= median)	Aliases
					**Mvb12b**	**Traf1**																
2C	LRS=(9 999 Chr2 33.04 49.66)	ConnD	L	B6	-0.156	0.228		**Mvb12b**	2	33.87378	8.65	3.91	33.60696	0.2668	0.3320	N	0	9.01	no	0	3xM; OC	2610528K11Rik; intron 3; Fam125b
		BVTV	H	B6	-0.324	0.246		**Traf1**	2	34.94763	7.973	4.67	33.60696	1.3407	-0.3210	N	0	7.63	no	0	3xM; OC	
width	16.62	TbSp	H,L	DBA	0.269/0.304	0.283/-0.224																
		TbN	L	B6	-0.251	0.313																
		CtAr	H	B6	-0.371	0.357																
					**Cdkn2b**																	
4E	LRS=(9 999 Chr4 81.54 92.00)	BV/TV_H	H	B6	0.219			**Cdkn2b**	4	89.3064	7.7919	6.09	88.04574	-1.2607	-0.1820	N	0	7.79	no	0	30-100xM; OB	
		TbN_H	H	B6	0.227																	
width	10.46	ConnD_L	L	B6	0.254																	
		ConnD_H	H	B6	0.26																	
		TbSp_H	H	DBA	-0.212																	
		SMI_L	L	DBA	-0.258																	
					**Eif4ebp1**	**Cyp4v3**	**Cnot7**															
8C	LRS=(9 999 Chr8 26.34 54.95)	TtAr	RCR	DBA	0.073	-0.107	-0.039	**Eif4ebp1**	8	27.27641	7.29	4.80	26.80498	0.4714	-0.2380	N	0	16.26	no	0	5xM OC and d5 OB	
		TbSp	RCR	B6	0.316	-0.424	-0.235	**Cyp4v3**	8	45.30592	9.313	3.33	45.56391	0.2580	0.1670	N	0	9.46	no	0	5xM OC +SI	CYP4v2 in human
width	28.61	TbN	RCR	DBA	-0.365	0.492	0.187	**Cnot7**	8	40.49317	11.488	3.54	39.9603	0.5329	0.328	N	0	6.87	no	0	1xM OC, OB	
		BMD	RCR	DBA	-0.083	0.079	0.23															
		SMI	RCR	B6	0.115	-0.129	-0.221															
		BMC	RCR	DBA	-0.115	0.003	0.191															
					**Ets1**	**Clmp**	**Kirrel3**															
9A	LRS=(9 999 Chr9 29.82 40.50)	TbTMD	L	B6	-0.523	0.417	0.338	**Kirrel3**	9	32.52116	9.706	10.61	31.15663	1.3645	-0.9590	N	0	10.24	no	0	1xM OC + OB	
		ConnD	H,L	DBA	0.562/0.49	-0.374/-0.35	-0.074/-0.111	**Ets1**	9	32.75284	7.379	4.39	35.00113	2.2483	0.0950	N	0	7.26	no	0	3xM; OB 5 d	
width	10.68	TbSp	H, L	B6	0.511/0.527	0.456/0.45	0.152/0.149	**Clmp**	9	40.72804	11.016	5.98	40.21308	0.5150	-0.7520	#N/A	#N/A	#N/A	#N/A	#N/A	40xM OB d21	9030425E11Rik
		TbN	H, L	DBA	-0.537/-0.533	-0.436/-0.426	-0.117/-0.153															
9D	LRS=(9 999 Chr9 112.62 124.12)				**C230053D17Rik**	**Ppp2r3a**																
		TbTh	RCR	DBA	0.127	-0.355		**C230053D17Rik**	9	121.32139	7.4619	7.67	121.01824	-0.303147	0.189	N	0	7.40	no	0	NA	
		BVTV	RCR	DBA	0.118	-0.163		**Ppp2r3a**	9	124.1512	7.4533	4.07	123.94265	-0.208556	-0.101	N	0	7.11	no	0	1.8xM; OC	
		ConnD	RCR	DBA	0.116	0.028																
		BMC	H	B6	-0.053	0.008																
		BMD	RCR	DBA	0.177	-0.338																
					**Rictor**																	
15A	LRS=(9 999 Chr15 0.53 12.11)	TbSp	H, L	DBA	-0.307/-0.38			**Rictor**	15	6.800083	10.049	3.13	7.16798	0.3679	-0.1870	#N/A	#N/A	#N/A	#N/A	#N/A	1xM; OB, OC	
		TbN	H, L	B6	0.318/0.405																	
width	11.58	BVTV	H	B6	0.321																	
		BMC	H	B6	0.391																	
		CtAr	H	B6	0.302																	
					**Camk4**	**Arhgap26**	**CD14**															
18C	LRS=(9 999 Chr18 29.98 47.08)	Imax	RCR	B6	-0.271	0.461	-0.131	**Camk4**	18	33.18821	7.385	4.04	34.24155	1.0533	0.0820	N	0	7.38	no	0	1xM; OB, OC	
		SMI	RCR	DBA	0.305	-0.081	0.258	**Arhgap26**	18	39.37217	7.305	2.96	39.04256	0.3296	-0.1500	N	0	7.38	yes	1	< 1xM; OB, OC	GRAF, Pp125
width	17.1	TbTh	RCR	B6	-0.342	0.267	-0.04	**CD14**	18	36.72537	8.268	5.15	34.2606	2.464768	0.243	N	0	8.39	no	0	3xM; OC	
		TtAr	RCR	B6	-0.203	0.512	-0.113	**Pcdhb4**	18	37.31041	7.328	4.04	35.81266	1.497744	-0.1	N	0	7.29	no	0	1xM; OB, OC	
		MOI	RCR	B6	-0.213	0.513	-0.133															
		CtAr	RCR	B6	-0.322	0.375	-0.121															

Supplemental Table S11. Evidence Supporting Potential Function of Select Candidate Genes

Loci ID	1.5-LOD Confidence Interval (Mb)	Gene	Type	chr	SNP ID (Mb)	Max LOD for eQTL	Max eQTL LOD Location (Mb)	Function (MGI, Genecards, PubMed, Coremine Medical Explorer, GO term)	Expression in bone cells in mice[a]
F-2B	26.56-31.01	Egfl7	Cn	2	rs50949904 (26.59)	NA	NA	Encodes epidermal growth factor-like domain-containing protein 7 which is a potent angiogenic factor. Egfl7 promotes growth and vascularization to the bone. Loss of function might impair bone mass.{1}	Moderately expressed in OC and OB
F-2C	33.04-49.66	Traf1	eQTL	2	34.95	4.67	33.61	There is some evidence suggesting potential role of Traf1 in bone resoprtion but direct support is limited. Traf1 has been shown to associate with the cytoplasmic tail of RANK.{2, 3} Traf1 is an adaptor protein for CD40 and increased expression of CD40 and TRAF1 proteins promotes activation of the NF-κB-dependent proinflammatory pathway.{4}	Moderately expressed in OC and OB
F-4E	81.54-92.00	Cdkn2b	eQTL	4	89.31	6.09	88.05	Encodes cyclin-dependent kinase inhibitor 2B known as one of G1/S checkpoint genes that controls cell cycle G1 progression. In addition, by inactivating CDK4/6, this protein results in increased phosphorylated Rb (pRB) which then positively influences osteoblastogenesis.{20} Overlapped with QTL of mouse's tibia width {21}	Highly expressed in differentiating OB (~6,500-7,600xM)
F-8C	26.98-49.55	Dlc1	Cn	8	rs30480037 (36.94)	NA	NA	Dlc1 was identified to be associated with osteoporosis in GWAS.{7} Dcl1 is over-expressed in the femoral head during bone overgrowth, thus may have a role in anti-apoptosis of OB.{8}	Enriched in pre-OB (12xM)
		Msr1	Cn	8	rs30713937 (39.62)	NA	NA	Existing evidence showed potential function of Msr1 in both OC and OB. Msr1-KO mice had reduced number of multinucleated OC and greater BMD.{5} Msr1 promotes bone formation (repair) by activating its receptors on pre-osteoblasts and upregulating the expression of pro-osteogenic factors (Col1, ALP, Osteocalcin, Runx2).{6} It is possible that Msr1 affects the coupling between OC and OB {6} but direct evidence is limited.	Highly expressed in OC (222xM) and Raw264.7 cells (167xM)
		Eif4ebp1	eQTL	8	27.28	4.8	26.8	Eif4ebp1 was identified to be associated with osteoporosis in GWAS.{7} Suppression of this gene by miR-138-5p reduces the proliferation and differentiaiton of OB.{9} However, no direct mechanistic connection exists.	Enriched in OC and pre-OB (5xM)
F-9A	29.82-40.50	Ets1	eQTL	9	32.75	4.39	35	Ets1 is a transcription factor that has two roles relevant to bone. First, it regulates the Cyp24a1 gene that controls degradation of 1,25(OH)2 D levels. Second, it regulated the *Runx2* promoter and regulates OB proliferation.{10} Knockdown of *Ets1* in pre-OB cells reduces the expression of osteogenic markers, ALP and BSP.{11} Suppression of *Ets1* expression by miRNA also reduced osteogenic gene expressions and OB differentiation.{12}	Enriched in early stage OD (3xM), differentiating OB (2xM), and highly enriched in bone (9xM)
		Clmp	eQTL	9	40.73	5.98	40.21	Encodes Coxsackie- and Adenovirus receptor-like membrane protein that affects tight junctions/cell-cell interactions. This gene is essential for intestine and adipocyte differentiation differentiation.{44}	Higly expressed in mature OB (40xM)
F-9D	112.62-124.12	Ppp2r3a	eQTL	9	124.15	4.07	123.94	Encodes serine/threonine-protein phosphatase 2A regulatory subunit B" subunit alpha. It is involved in the negative control of cell growth and division. It regulates β-catenin stability by dephosphorylating APC, thereby inhibiting Wnt signaling.{53}	Slightly higher expression in early stage OB and OC (3xM)
F15-A	0.53-12.11	Rictor	eQTL	15	6.8	3.13	7.17	Rictor is a core subunit of mTORC2 which plays a role in osteoblast differentiation.{35, 36} Rictor-deficient MSC showed impaired osteogenic potential.{37, 38, 39} Osteoblast-specific knowout mice had increased cortical porosity and marrow area in the femur and thinner trabeculae in the L5 vertebrae.{39} Recent study revealed that this gene is also essential for osteoclastogenesis.{45}	Enriched in early stage OB (4xM) and consitutively expressed in differentiating OB, mature OB, and OC
F-15C	59.80-72.53	Fam135b	Cn	15	rs50815982 (71.46)	NA	NA	Encodes a protein that promotes growth, migration, and invasion of cancer cells.{48} However, Fam135b has not been studied in the context of bone biology.	Slightly higher expression in mature OB (1.2xM)

F-18C	0.02-25.78	Apc	Cn	18	rs51271694 (34.32)	NA	NA	APC is a negative regulator of Wnt signaling pathway{26} In high density association study of 383 candidate genes: APC was associated with femoral neck and lumbar spine volumetric BMD.{27} A candidate gene study of APC copy number variation in a twin population showed the effect of genetic variation in this gene on BMD.{28} Mice carrying a heterozygous loss of function mutation in APC display significantly increased BMD of the distal femur.{29} In addition, mice with OB-specific deletion of APC displayed growth retardation, increased accumulation of bone matrix.{29} Collectively, APC plays important role in bone homeostasis.	Constitutively expressed in OB and OC
		Camk4	eQTL	18	33.19	4.04	34.24	Encodes a serine-threonine protein kinase that is activated through a calmodulin depended pathway when intracellular Ca increases. Camk4 activity is increased after RANKL stimulation, and siRNA mediated-Camk4 knockdown reduces RANKL-induced osteoclastogenesis. Consistent with an essential role for Camk4 in osteoclast biology, Camk4 knockout mice have fewer osteoclasts and increased bone volume.{52}	Enriched in mature OB (4xM)
F-20C	51.90-69.44	Vgll1	Cn	X	rs48289336, rs29060172 (57.10)	NA	NA	Encodes vestigial like family member 1, which modify gene transcription by competing with YAP transcription factors for binding to TEADs.{41} Yap1 drive osteoclastogenesis.{42} While research shows that Vgll4 can promote OB differentiation by antagonizing TEADs-mediated inhibition of Runx2 transcription,{43} no research has examined the role of *Vgll1* in bone cell biology.	Constitutively expressed in OB and OC

Abbreviations: Mb = mega-basepair (Build GRCm38/mm10), chr = chromosome, NA = Not applicable,
BMD = bone mineral density, OB = osteoblast, OC = osteoclast, xM = times the median expression in other cell types,
KO = knockout, ALP = alkaline phosphatase; BSP = bone sialoprotein, RANK = Receptor activator of nuclear factor κ B

Website: MGI: http://www.informatics.jax.org/
Uniprot Reactome human: https://www.uniprot.org/uniprot/Q9BYX4
Uniprot Reactome mouse: https://www.uniprot.org/uniprot/Q8R5F7

[a]Expression data from BioGPS: http://biogps.org/#goto=welcome

References:

1 Chim et al. J. Cell. Physiol. 2015;230: 82–94. doi: 10.1002/jcp.24684
2 Kim et al. FEBS Letters. 1999;443:297-302. doi: 10.1016/s0014-5793(98)01731-1
3 Wong et al. J Biol Chem. 1998;273(43):28355-28359. doi: 10.1074/jbc.273.43.28355
4 Cheng et al. 2018;47(6):455-460. doi: 10.1080/03009742.2018.1432684.
5 Takemura et al. Biochem Biophys Res Comm. 2010;391:1675-1680. doi: 10.1016/j.bbrc.2009.12.126
6 Zhao et al. Theranostics. 2020;10(1): 17-35. doi: 10.7150/thno.36930
7 Morris et al. Nat Genet. 2019 Feb;51(2):258-266. doi: 10.1038/s41588-018-0302-x
8 Liu et al. J Orthop Surg Res . 2017 Jan 17;12(1):8. doi: 10.1186/s13018-017-0510-6
9 Sun et al. Int. J. Mol. Sci. 2016;17(236):1-15. doi:10.3390/ijms17020236
10 Zhang et al. J Biol Chem. 2009;284(5):3125-35. doi: 10.1074/jbc.M807466200
11 Koyama T and Kamemura K. Exp Cell Res. 2015;338;194-202. doi: 10.1016/j.yexcr.2015.08.009
12 Fan et al. Biochem Biophys Res Commun. 2020 Apr 30;525(2):498-504. doi: 10.1016/j.bbrc.2020.02.126.
13 Negishi-Koga et al. Nature Medicine. 2011;17(11): 1473-1481. doi:10.1038/nm.2489
14 Noriyoshi et al., Blood, Vol 87, No 9 (May 1). 1996: pp 3704-3710
15 Andrade et al. Sci Transl Med. 2017 Jan 25;9(374):eaai9338. doi: 10.1126/scitranslmed.aai9338.
16 Kacena MA and Ciovacco W. Adv Exp Med Biol. 2010;658:31-41. doi: 10.1007/978-1-4419-1050-9_4.
17 Tang et al. Theranostics. 2020 Jan 12;10(5):2229-2242. doi: 10.7150/thno.40559.
18 Alvarez et al. Stem Cells Dev. 2018 May 15;27(10):671-682. doi: 10.1089/scd.2017.0178.
19 Bord et al. Bone. 2005 May;36(5):812-9. doi: 10.1016/j.bone.2004.12.006.
20 Flowers et al. Cancer Res. 2013 Apr 1;73(7):2150-8. doi: 10.1158/0008-5472.CAN-12-1745.
21 https://rgd.mcw.edu/rgdweb/report/qtl/main.html?id=27226724
22 JBMR Plus. 2019 Nov 11;3(12):e10241. doi: 10.1002/jbm4.10241
23 Sun et al. Bone. 2008 Mar;42(3):547-53. doi: 10.1016/j.bone.2007.11.004.
24 https://rgd.mcw.edu/rgdweb/report/gene/main.html?id=70962
25 Yang et al. Mol Med Rep. 2019 Feb; 19(2): 1065–1073. doi: 10.3892/mmr.2018.9752
26 McCord et al. Front. Cell. Neurosci. 2017;11:318. doi: 10.3389/fncel.2017.00318

27 Yeages et al. J Bone Miner Res 2009;24:2039-49
28 Chew et al. Bone 2012; 51:939–943
29 Holmen et al. J Biol Chem 2005;280(22): 21162-8.
30 Sørensen et al. J Bone Min Met 2007;25:36-45. doi: 10.1007/s00774-006-0725-9
31 Bethel et al. Osteoporos Int 2016;27:1755–1763. doi: 10.1007/s00198-015-3439-9
32 Beamer et al. J Bone Miner Res. 2001 Jul;16(7):1195-206. doi: 10.1359/jbmr.2001.16.7.1195.
33 Bouxsein et al. J Bone Miner Res. 2004 Apr;19(4):587-99. doi: 10.1359/JBMR.0301255.
34 Li et al. Funct Integr Genomics (2002) 1:367–374. doi: 10.1007/s10142-001-0045-z
35 Cheng J and Long F. Bone Res. 2018 Jan 30;6:1. doi: 10.1038/s41413-017-0004-5.
36 Cheng et al. J Bone Miner Res. 2015 Feb;30(2):369-78. doi: 10.1002/jbmr.2348.
37 Sen et al. J Bone Miner Res. 2014 Jan;29(1):78-89. doi: 10.1002/jbmr.2031.
38 Martin et al. Stem Cells. 2015 Apr;33(4):1359-65. doi: 10.1002/stem.1931.
39 Liu et al. Bone. 2016 Sep;90:50-8. doi: 10.1016/j.bone.2016.05.010.
40 Mamm Genome. 1999 Nov;10(11):1043-9. doi: 10.1007/s003359901159.
41 Landin-Malt et al. Gene. 2016;591(1):292-303 doi: 10.1016/j.gene.2016.07.028
42 Zhao et al. Bone. 2018 May;110:177-186. doi: 10.1016/j.bone.2018.01.035.
43 Suo et al. Sci Adv. 2020 Oct 23;6(43):eaba4147. doi: 10.1126/sciadv.aba4147.
44 Tashiro et al. Stem Cells 2009 Aug;27(8):1802-11. doi: 10.1002/stem.108.
45 Xu et al. J Bone Miner Res. 2021 Jun 22. doi: 10.1002/jbmr.4398.
46 Courel et al. Dev Dyn. 2008;237(5):1232-42. doi:10.1002/dvdy.21516.
47 Kikuchi et al. Stem Cell. 2007;25:2439–2447. doi: 10.1634/stemcells.2007-0207
48 Bi et al. Gene. 2021 Mar 10;772:145358. doi: 10.1016/j.gene.2020.145358.
49 Fontana et al. J Bone Miner Res. 2017; 32(6):1332-1342.
50 Di Benedetto et al. Journal of Cell Science. 2010; 123:2640-2648. doi:10.1242/jcs.067777
51 Revollo al. J Bone Miner Res. 2015;30(2):274-285. doi: 10.1002/jbmr.2323.
52 Sato et al. Nature Medicine. 2006;12(12):1410-1416.
53 Joni et al, Science. 1999;283(5410): 2089-2091 doi: 10.1126/science.283.5410.2089
